# Nonheme Fe^IV^=O Complexes Supported by Four Pentadentate Ligands: Reactivity toward H- and O- Atom Transfer Processes

**DOI:** 10.1021/acs.inorgchem.3c02526

**Published:** 2023-11-01

**Authors:** Yong Li, Reena Singh, Arup Sinha, George C. Lisensky, Matti Haukka, Justin Nilsson, Solomon Yiga, Serhiy Demeshko, Sophie Jana Gross, Sebastian Dechert, Ana Gonzalez, Giliandro Farias, Ola F. Wendt, Franc Meyer, Ebbe Nordlander

**Affiliations:** † Chemical Physics, Department of Chemistry, Lund University, Box 124, Lund SE-221 00, Sweden; ‡ Department of Chemistry, Beloit College, 700 College Street, Beloit, Wisconsin 53511, United States; § Department of Chemistry, University of Jyväskylä, P.O. Box-35, Jyväskylä FI-40014, Finland; ∥ Centre for Analysis and Synthesis, Department of Chemistry, Lund University, P.O. Box 124, Lund SE-22100, Sweden; ⊥ Georg-August Universität Göttingen, Institut für Anorganische Chemie, Tammanstrasse 4, Göttingen D-37077, Germany; # MAX IV Laboratory, Lund University, P.O. Box 118, Lund SE-221 00, Sweden; ¶ Department of Chemistry, Federal University of Santa Catarina, Florianópolis 88040900, Santa Catarina, Brazil

## Abstract

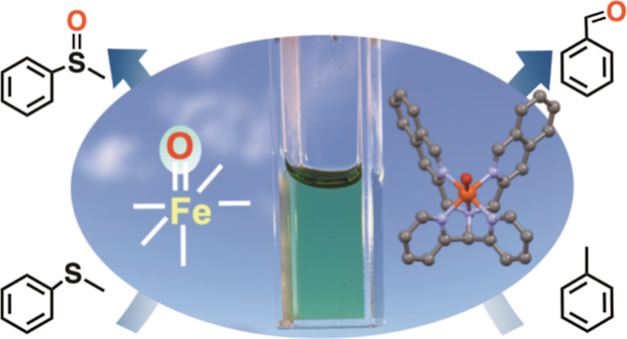

Four new pentadentate N5-donor ligands, [*N*-(1-methyl-2-imidazolyl)methyl-*N*-(2-pyridyl)-methyl-*N*-(bis-2-pyridylmethyl)-amine] (**L**^**1**^), [*N*-bis(1-methyl-2-imidazolyl)methyl-*N*-(bis-2-pyridylmethyl)amine] (**L**^**2**^), (*N*-(isoquinolin-3-ylmethyl)-1,1-di(pyridin-2-yl)-*N*-(pyridin-2-ylmethyl)methanamine (**L**^**3**^), and *N*,*N*-bis(isoquinolin-3-ylmethyl)-1,1-di(pyridin-2-yl)methanamine (**L**^**4**^), have been synthesized based on the N4Py ligand framework, where one or two pyridyl arms of the N4Py parent are replaced by (*N*-methyl)imidazolyl or *N*-(isoquinolin-3-ylmethyl) moieties. Using these four pentadentate ligands, the mononuclear complexes [Fe^II^(CH_3_CN)(**L**^**1**^)]^2+^ (**1a**), [Fe^II^(CH_3_CN)(**L**^**2**^)]^2+^ (**2a**), [Fe^II^(CH_3_CN)(**L**^**3**^)]^2+^ (**3a**), and [Fe^II^(CH_3_CN)(**L**^**4**^)]^2+^ (**4a**) have been synthesized and characterized. The half-wave potentials (*E*_1/2_) of the complexes become more positive in the order: **2a** < **1a** < **4a** ≤ **3a** ≤ [Fe(N4Py)(CH_3_CN)]^2+^. The order of redox potentials correlates well with the Fe–N_amine_ distances observed by crystallography, which are **2a** > **1a** ≥ **4a** > **3a** ≥ [Fe(N4Py)(CH_3_CN)]^2+^. The corresponding ferryl complexes [Fe^IV^(O)(**L**^**1**^)]^2+^ (**1b**), [Fe^IV^(O)(**L**^**2**^)]^2+^ (**2b**), [Fe^IV^(O)(**L**^**3**^)]^2+^ (**3b**), and [Fe^IV^(O)(**L**^**4**^)]^2+^ (**4b**) were prepared by the reaction of the ferrous complexes with isopropyl 2-iodoxybenzoate (IBX ester) in acetonitrile. The greenish complexes **3b** and **4b** were also isolated in the solid state by the reaction of the ferrous complexes in CH_3_CN with ceric ammonium nitrate in water. Mössbauer spectroscopy and magnetic measurements (using superconducting quantum interference device) show that the four complexes **1b, 2b, 3b**, and **4b** are low-spin (*S* = 1) Fe^IV^=O complexes. UV/vis spectra of the four Fe^IV^=O complexes in acetonitrile show typical long-wavelength absorptions of around 700 nm, which are expected for Fe^IV^=O complexes with N4Py-type ligands. The wavelengths of these absorptions decrease in the following order: 721 nm (**2b**) > 706 nm (**1b**) > 696 nm (**4b**) > 695 nm (**3b**) = 695 nm ([Fe^IV^(O) (N4Py)]^2+^), indicating that the replacement of the pyridyl arms with (*N*-methyl) imidazolyl moieties makes **L**^**1**^ and **L**^**2**^ exert weaker ligand fields than the parent N4Py ligand, while the ligand field strengths of **L**^**3**^ and **L**^**4**^ are similar to the N4Py parent despite the replacement of the pyridyl arms with *N*-(isoquinolin-3-ylmethyl) moieties. Consequently, complexes **1b** and **2b** tend to be less stable than the parent [Fe^IV^(O)(N4Py)]^2+^ complex: the half-life sequence at room temperature is 1.67 h (**2b**) < 16 h (**1b**) < 45 h (**4b**) < 63 h (**3b**) ≈ 60 h ([Fe^IV^(O)(N4Py)]^2+^). Compared to the parent complex, **1b** and **2b** exhibit enhanced reactivity in both the oxidation of thioanisole in the oxygen atom transfer (OAT) reaction and the oxygenation of C–H bonds of aromatic and aliphatic substrates, presumed to occur *via* an oxygen rebound process. Furthermore, the second-order rate constants for hydrogen atom transfer (HAT) reactions affected by the ferryl complexes can be directly related to the C–H bond dissociation energies of a range of substrates that have been studied. Using either IBX ester or H_2_O_2_ as an oxidant, all four new Fe^II^ complexes display good performance in catalytic reactions involving both HAT and OAT reactions.

## Abstract 2

Four Fe^IV^=O complexes of pentadentate ligands based on the N4Py ligand framework have been synthesized and characterized. Their abilities to act as reagents for oxygen atom and hydrogen atom transfer reactions have been investigated.

## Introduction

Oxidations of C–H bonds *via* hydrogen and/or oxygen atom transfer (HAT/OAT) are very important processes in biology. It has been conclusively demonstrated that the active intermediates in several nonheme iron enzymes involve high-valent Fe^IV^=O moieties. For example, mononuclear nonheme Fe^IV^=O cores have been detected in the catalytic cycles of α-ketoglutarate-dependent oxygenases (taurine dioxygenase^1^ and propyl-4-hydroxylase^2^), halogenases (cytochrome c_3_ halogenases^3,4^ with chlorine or bromine and SyrB2 halogenase^5,6^), and pterin-dependent hydroxylases (tyrosine^7^ and phenylalanine hydroxylases^8^). In order to gain mechanistic insights into their functions in biology, chemists have mounted a significant effort to mimic biological Fe^IV^=O entities. Thus, several Fe^IV^=O complexes have been synthesized to shed light on the electronic structures and spectroscopic properties of these high-valent iron–oxo intermediates.^9−11^

Unlike the Fe^IV^=O intermediates found in nonheme enzymes, which have been proven to be in the high-spin (*S* = 2) state, most of the synthesized complexes are found to be in the low-spin (*S* = 1) state, with only a few exceptions being in the high-spin state.^9,11,12^ Density functional theory (DFT) calculations indicate that high-spin *S* = 2 Fe^IV^=O complexes should be more reactive than the corresponding low-spin versions in hydrogen atom transfer,^13,14^ although it has been demonstrated that access to the *S* = 2 spin state is not required for HAT reactivity.^15^ For pseudo-octahedral complexes, a small energy gap between the d_x2-y2_ and d_*xy*_ orbitals in the equatorial plane may facilitate the formation of a high-spin complex; this might be achieved by introducing weak-field equatorial donor entities in pentadentate ligands.^9,16^ A number of studies have shown that weakening of the ligand field will also enhance the reactivities of the pseudo-octahedral low-spin Fe^IV^=O complexes (*vide infra*),^17^ which may be rationalized by so-called two-state reactivity, *i.e.*, spin-crossover to a high-spin state in the transition state of an HAT reaction.^18−20^ Furthermore, recent investigations have described a number of highly reactive ferryl complexes.^21,22^

Among synthesized Fe^IV^=O complexes, [Fe^IV^(O)(N4Py)]^2+^ has been shown to exhibit powerful oxidative reactivity toward both alkanes and arenes, enabling it to cleave strong C–H bonds while possessing considerable thermal stability that makes it possible to study the complex in detail.^23^ Considering this, many derivatives of the N4Py ligand have been synthesized^24^ to investigate the influence of ligand modifications on the steric and electronic properties of their iron complexes and (thus) the reactivities of the Fe^IV^=O complexes.^25^ It is apparent that both structural (steric) and electronic factors influence the reactivities of such ferryl–oxo complexes, but it remains difficult to predict such reactivities.

**Figure 1:**
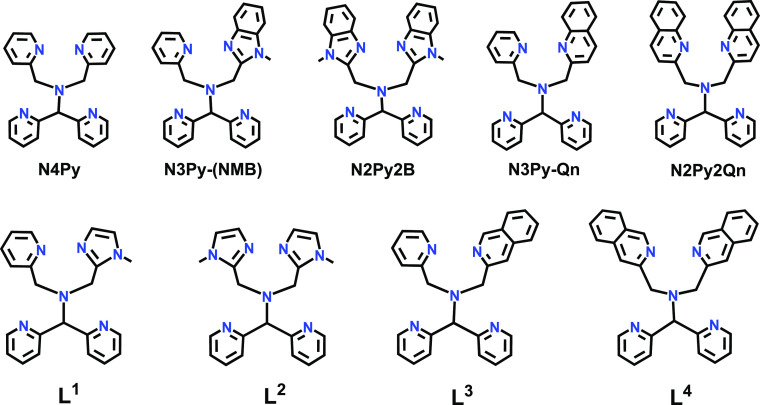
Structures of previously synthesized and new pentadentate ligands (**L**^1^–**L**^**4**^).

In a previous study, we explored the chemistry of the Fe^IV^=O complexes of (*N*-methyl)benzimidazolyl derivatives of N4Py, *viz*., N3Py-(NMB) and N2Py2B (Figure 1).^26^ The complexes [Fe^IV^(O)(L)]^2+^ (L = N3Py-(NMB), N2Py2B) were found to be considerably more reactive than the parent [Fe^IV^(O)(N4Py)]^2+^ complex, with each replacement of a pyridyl unit with an (*N*-methyl)benzimidazolyl unit leading to an increase in HAT rate by almost 1 order of magnitude for a range of benchmark substrates. At the same time, the half-lives of the ferryl–oxo complexes were found to decrease for each (*N*-methyl)benzimidazolyl unit introduced so that the half-life at room temperature of [Fe^IV^(O)(N2Py2B)]^2+^ was found to be only ca. 2.5 h, as compared to 60 h for the parent complex. The reactivities could also be correlated to a weakening of the equatorial field exerted by the pentadentate ligands, as manifested by the frequency of a long-wavelength absorption in the vicinity of 700 nm that is characteristic for this kind of complex (*vide infra*). Despite the relatively short half-life of [Fe^IV^(O)(N2Py2B)]^2+^ and related [Fe^IV^(O)(N2Py2Qn)]^2+^ (*cf*. Figure 1), Que and co-workers managed to crystallize and determine the solid-state structure of these two complexes.^25^ The crystal structures illustrated how the ligand modifications influence the Fe^IV^=O environment relative to that of the parent N4Py complex. For example, the steric bulk of the quinolyl donors causes a tilt of the Fe^IV^=O unit away from a linear N–Fe^IV^=O arrangement by 10°, and the (*N*-methyl)benzimidazolyl moieties in [Fe^IV^(O)(N2Py2B)]^2+^ also cause a relatively minor tilt of 3°. It was also concluded that longer Fe–N distances correlate linearly with log *k*_2_′ values for O- and H- atom transfer rates and that the electrophilicity of the Fe^IV^=O center can be increased by the weakening of the ligand field. The steric effect on ligand coordination, and thus effective ligand field, has been explored in several other studies on the Fe^IV^=O complexes of ligands based on the N4Py framework.^24,27,28^

We decided to prepare new pentadentate ligands based on the same framework, where the steric influence of the ligand moieties is expected to be minimal, and the effective ligand field therefore is expected to primarily be related to the electronic properties of the various N-donor entities. Based on the ligands N2Py2Qn and N2Py2B, four new ligands (**L**^**1**^–**L**^**4**^, Figure 1) were designed and synthesized. Because of the sizes and orientations of the imidazolyl and isoquinolinyl moieties in **L**^**1**^–**L**^**4**^, it was expected that their coordination to a metal ion will lead to relatively “open” structures, where the steric influence of the ligands is minor and similar to that of the N4Py ligand, and this was indeed found to be the case (*vide infra*). The mononuclear Fe^II^ complexes of these four new pentadentate ligands have been synthesized, and the corresponding Fe^IV^=O complexes have also been prepared and characterized. The reactivities of the new Fe^IV^=O units toward external substrates have been investigated and are discussed below.

## Results and Discussion

### Synthesis and Characterization of Ligands

**Scheme 1:**
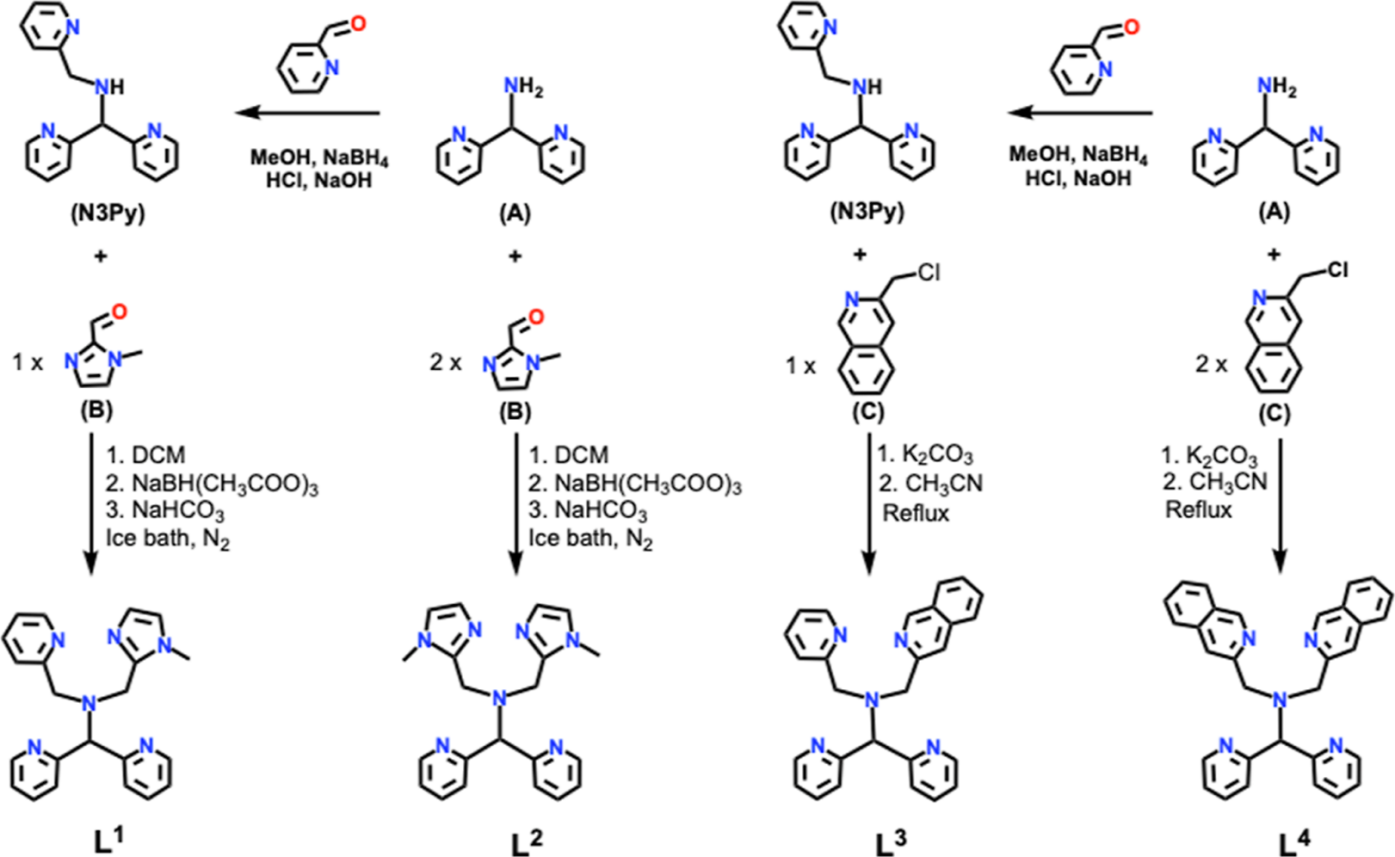
Schematic of the Synthetic Routes for Ligands **L**^**1**^–**L**^**4**^

The syntheses of the four ligands are summarized in Scheme 1. Syntheses of **L**^**1**^ and **L**^**2**^ were achieved by a previously reported method,^29^ involving reductive amination to achieve condensation of 1-methyl-1*H*-imidazole-2-carbaldehyde (B, Scheme 1) with the secondary amine of *N*-[di(2-pyridinyl)methyl]-*N*-(2-pyridinylmethyl)methylamine (N3Py) to form **L**^**1**^, or the primary amine of bis(2-pyridyl)methylamine (A, Scheme 1) to form **L**^**2**^. Similarly, ligands **L**^**3**^ and **L**^**4**^ were synthesized by reaction of N3Py or bis(2-pyridyl)methylamine with one or two equivalents of 3-(chloromethyl)isoquinoline (C, Scheme 1) in the presence of K_2_CO_3_ (NMR Figures S1–S7, Supporting Information).

### Synthesis and Characterization of Fe^II^ Complexes

**Figure 2:**
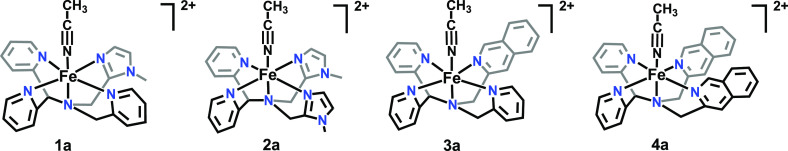
Schematic drawings of the cationic Fe^II^ complexes **1a**–**4a**.

The ferrous complexes [Fe^II^(CH_3_CN)(**L**^**1**^)](ClO_4_)_2_ (**1a**·(ClO_4_)_2_), [Fe^II^(CH_3_CN)(**L**^**2**^)](ClO_4_)_2_ (**2a**·(ClO_4_)_2_), [Fe^II^(CH_3_CN)(**L**^**3**^)](ClO_4_)_2_ (**3a**·(ClO_4_)_2_), and [Fe^II^(CH_3_CN)(**L**^**4**^)](ClO_4_)_2_ (**4a**·(ClO_4_)_2_) (Figure 2) were prepared by reaction of the relevant ligand with Fe(ClO_4_)_2_·*x*H_2_O in acetonitrile solvent in a glovebox. The triflate and tetrafluoroborate salts [Fe^II^(CH_3_CN)(**L**^**1**^)](OTf)_2_ (**1a**·(OTf)_2_), [Fe^II^(CH_3_CN)(**L**^**2**^)](OTf)_2_ (**2a**·(OTf)_2_), [Fe^II^(**L**^**3**^)(CH_3_CN)](OTf)_2_ (**3a**·(OTf)_2_), [Fe^II^(CH_3_CN)(**L**^**4**^)](OTf)_2_ (**4a**·(OTf)_2_), and [Fe^II^(CH_3_CN)(**L**^**4**^)](BF_4_)_2_ (**4a**·(BF_4_)_2_) were also prepared from the appropriate starting materials. The high-resolution mass spectra of **1a**–**4a** were all consistent with those of M-CH_3_CN ions. Both [Fe^II^(**L**^***n***^)X]^+^ and [Fe^II^(**L**^***n***^)]^2+^ (*n* = 1–4, X = ClO_4_^–^ or OTf^–^) peaks were observed (*cf*. the Experimental Section and Supporting Information). A ferric oxo-bridged dinuclear complex [Fe^III^_2_(**L**^**2**^)_2_(μ-O)](OTf)_2_ (**5**·(OTf)_2_) was obtained by the reaction of **L**^**2**^ with Fe(OTf)_2_·2MeCN under an ambient atmosphere and was identified *via* single-crystal X-ray diffraction (*cf*. the Experimental Section and Supporting Information).

#### NMR Spectroscopy

The proton nuclear magnetic resonance (^1^H NMR) spectrum of **1a**·(ClO_4_)_2_ in CD_3_CN indicates a low-spin state at room temperature, while the analogous spectrum in dimethyl sulfoxide-*d*6 (DMSO-*d*_6_) shows the presence of a mixture of high-spin and low-spin states for the same complex. The ^1^H NMR spectra of **2a**·(ClO_4_)_2_ show the presence of a high-spin complex in both CD_3_CN and DMSO-*d*_6_ at room temperature. The ^1^H NMR spectra furthermore indicate that **3a**·(ClO_4_)_2_ and **4a**·(ClO_4_)_2_ are low-spin in CD_3_CN solution at ambient temperature; however, the recordings of the corresponding spectra of these two complexes in DMSO-*d*_6_ show the presence of high-spin complexes (see Figures S8–S15, Supporting Information). We anticipate that the spectra recorded in DMSO-*d*_6_ contain DMSO coordinated *via* its oxygen atom to the iron ion. The σ-donor DMSO ligand exerts a weaker field than acetonitrile, which is a σ-donor and π-acceptor.

#### Mössbauer Spectroscopy and Magnetic Measurements

**Figure 3:**
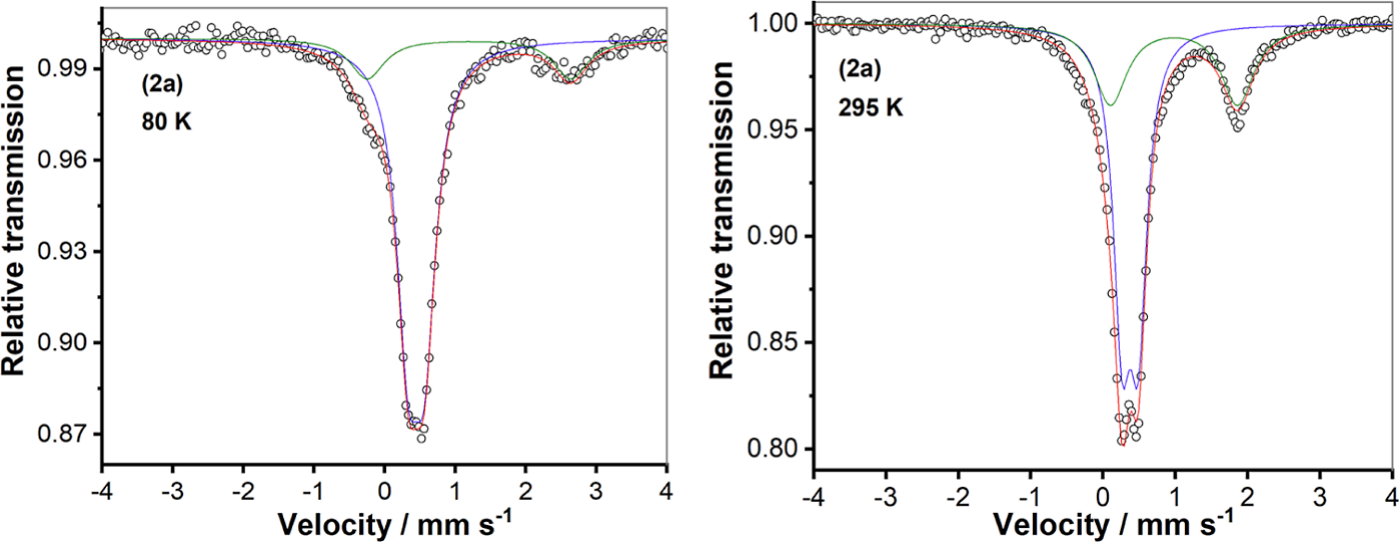
Zero-field Mössbauer spectra of the solid sample of complex **2a**·(OTf)_2_ at 80 K (left) and 295 K (right).

The ^57^Fe-enriched samples [^57^Fe^II^(CH_3_CN)(**L**^**X**^)] (OTf)_2_ (X = 1–4) were prepared from ^57^Fe(OTf)_2_. Zero-field Mössbauer spectra of solid samples of these ^57^Fe-enriched complexes were recorded at 80 K (Figure S16). The isomer shift (δ) and quadrupole splitting (Δ*E*_Q_) values are listed in Table 1. These values confirm the low-spin state of the Fe^II^ ions in **1a**, **3a**, and **4a**; however, a mixture of high- and low-spin states for **2a** is noted. As shown in Figure 3, the zero-field Mössbauer spectrum of **2a**·(OTf)_2_ at 80 K can be fitted to the presence of a low-spin Fe^II^ main species (82%) and a second species (18%) containing Fe^II^ in the high-spin state. Mössbauer measurements at different temperatures revealed that the fraction of Fe^II^ in the high-spin state increases from 18% at 80 K to 35% at 295 K. It may be concluded that an incomplete spin-crossover transition occurs in the solid sample.

**Table 1:** Mössbauer Parameters for the Triflate Salts of Solid **1a**–**4a**

complex	relative area (%)	δ (mm s^–^^1^)	Δ*E*_Q_ (mm s^–^^1^)
**1a** (80 K)	100	0.43	0.17
**2a** (80 K)	82	0.45	0.24
	18	1.19	2.88
**2a** (295 K)	65	0.37	0.23
	35	0.98	1.76
**3a** (80 K)	94	0.39	0.24
**4a** (80 K)	100	0.38	0.36

**Figure 4:**
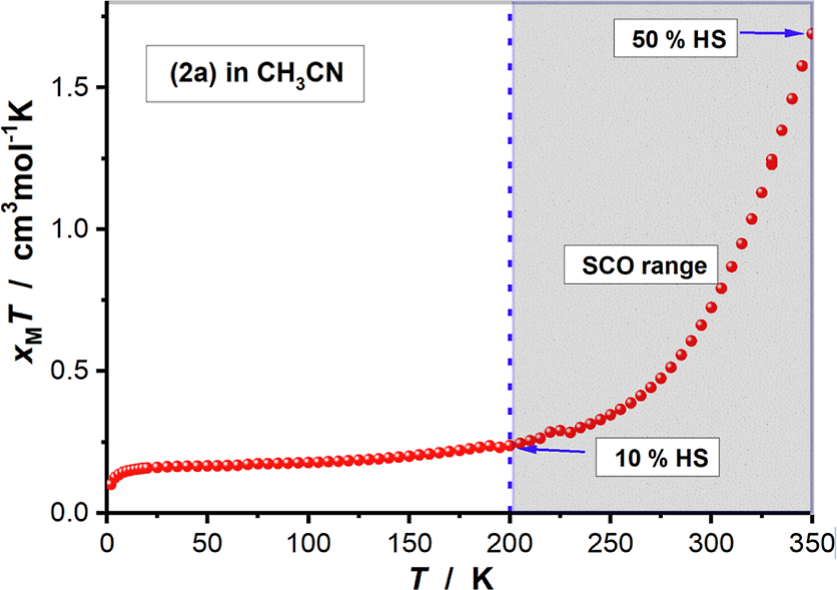
Plot of χ_M_*T* versus *T* for **2a**·(OTf)_2_ in acetonitrile (SCO = spin-crossover, HS = high-spin).

Magnetic measurements [using a superconducting quantum interference device (SQUID)] and the Mössbauer spectrum of **2a**·(OTf)_2_ in frozen acetonitrile were also recorded to investigate the spin-crossover properties in solution. As shown in Figure 4, **2a** is predominantly diamagnetic (*i.e.*, low-spin) at temperatures below 200 K in CH_3_CN. Above 200 K, the fraction of Fe^II^ in the high-spin state increases, which is attributed to the spin-crossover. At 350 K, about 50% of the sample is in the high-spin state. The Mössbauer spectrum at 80 K, with parameters for the main signal δ = 0.43 mm s^–1^ and Δ*E*_Q_ = 0.22 mm s^–1^ (Figure S17), confirms that **2a** is also predominantly in the low-spin state (92%) in frozen solution with only a small fraction in the high-spin Fe^II^ form (8%, δ = 1.20 mm s^–1^ and Δ*E*_Q_ = 3.12 mm s^–1^). The Mössbauer spectra in both solid state and frozen acetonitrile solution and the magnetic measurements are all in agreement, indicating that the spin-crossover takes place for **2a**·(OTf)_2_.

#### Absorption Spectroscopy

**Figure 5:**
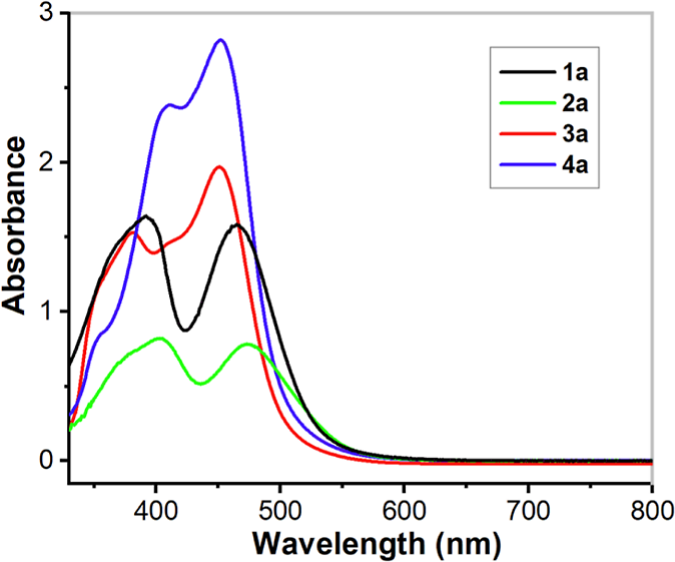
UV/vis spectra of complexes **1a**·(ClO_4_)_2_, **2a**·(ClO_4_)_2_, **3a**·(ClO_4_)_2_, and **4a**·(ClO_4_)_2_ (0.25 mM) in acetonitrile at 298 K. CT bands appear at λ_max_ = 393 nm (ε ≈ 6280 M^–1^ cm^–1^) and 466 nm (ε ≈ 6000 M^–1^ cm^–1^) for **1a**, at λ_max_ = 380 nm (ε ≈ 6080 M^–1^ cm^–1^) and 451 nm (ε ≈ 7880 M^–1^ cm^–1^) for **3a**, at λ_max_ = 410 nm (ε ≈ 9560 M^–1^ cm^–1^) and 452 nm (ε ≈ 11,280 M^–1^ cm^–1^) for **4a**, while complex **2a** shows weaker absorbance and the CT bands appear at λ_max_ = 404 nm (ε ≈ 3300 M^–1^ cm^–1^) and 475 nm (ε ≈ 3160 M^–1^ cm^–1^).

The UV/vis spectra of ligands **L**^**1**^–**L**^**4**^ in an acetonitrile solution show high-intensity bands in the UV region. Upon coordination to Fe^II^, two new charge transfer (CT) bands appear in the visible region for all of the complexes/ligands (Figure 5). These CT bands can be assigned as metal-to-ligand charge transfer (MLCT) bands arising from electron transfer from the Fe^II^ t_2g_ orbitals to the π* orbitals of the ligand. Complex **2a**·(ClO_4_)_2_ exhibits significantly weaker absorbance (smaller observed extinction coefficients) relative to those of the other three complexes. The absorbance values for **2a**·(ClO_4_)_2_ in acetonitrile increase when the temperature is lowered from 298 to 235 K (Figure S18). These observations can be rationalized by **2a**·(ClO_4_)_2_ undergoing a spin-crossover, as discussed above, *i.e*,*.* the complex exists as a mixture of high-spin and low-spin forms in solution and the population of the low-spin state increases as the temperature decreases, in agreement with the SQUID and Mössbauer results for **2a**·(OTf)_2_ in CH_3_CN (*vide supra*). Variable-temperature NMR spectroscopy of **2a**·(ClO_4_)_2_ (Figure S19) also confirmed the spin-crossover of the complex.

On the other hand, complexes **3a** and **4a**, which contain isoquinoline substituents in the framework of the pentadentate ligands, only show low-spin Fe^II^ ions. This observation is in contrast to that for the analogous quinoline complexes, [Fe^II^(CH_3_CN)(N3Py-Qn)]^2+^ and [Fe^II^(CH_3_CN)(N2Py2Qn)]^2+^, which are found to be in a mixture of high- and low-spin states.^28^ Furthermore, the absorption spectra of **3a** and **4a** differ from those of other Fe^II^ complexes of N4Py and its derivatives in that the longer-wavelength CT absorption (around 450 nm) exhibits a larger extinction coefficient than that of the shorter-wavelength CT band. This phenomenon is clearly attributable to the presence of the isoquinoline substituents in ligands **L**^**3**^ and **L**^**4**^ and suggests that the lowering of the π* orbitals on the isoquinoline substituents leads to enhanced mixing between the metal d(π) and ligand (π*) orbitals, therefore making it a better π-acceptor than pyridine or even quinoline. In order to elucidate how the isoquinoline substituents affect the absorptions, a time-dependent DFT (TD-DFT) analysis of **4a** was undertaken, and a full account of this analysis is given in the Supporting Information (Table S1).

**Figure 6:**
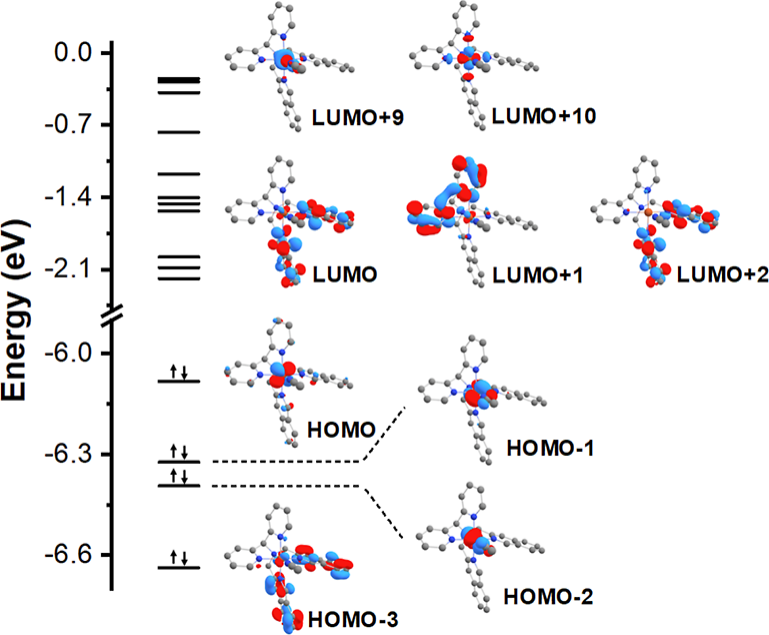
Calculated frontier orbitals for complex **4a** within the PBE0/Def2-TZVP(-f) level of theory.

Figure 6 shows the calculated frontier orbitals for **4a**. The highest occupied molecular orbital (HOMO), HOMO–1, and HOMO–2 are largely derived from the d_*xy*_, d_*xz*_, and d_*yz*_ orbitals, respectively, of the Fe^II^ center. At the same time, HOMO–3 is a π orbital spread over the isoquinoline moiety, with a small contribution of the d_*xz*_ orbital of the Fe^II^ metal. The lowest unoccupied molecular orbital (LUMO) and LUMO+2 orbitals are π* orbitals located over the isoquinoline moiety, and LUMO+1 is a π* orbital spread over the coordinated pyridines. The LUMO+9 and LUMO+10 are the empty d_*z*_^2^ and d_*x*_^2^_–y_^2^, respectively.

**Figure 7:**
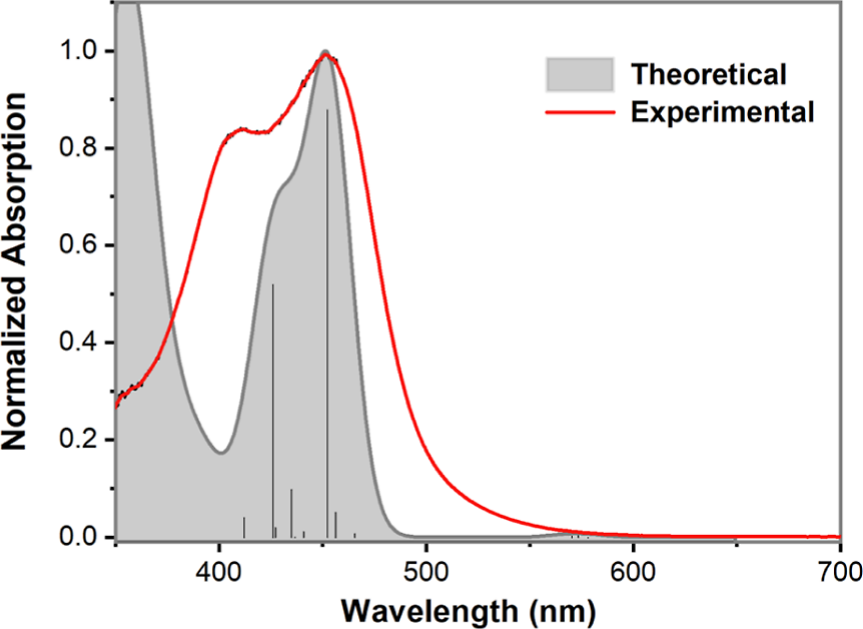
Experimental absorption spectrum of complex **4a** in CH_3_CN and the theoretical absorption spectrum convoluted with Gaussians of 30 nm width. The theoretical spectrum was shifted by 64 nm (0.45 eV) from 381 nm (3.25 eV) to 445 nm (2.80 eV) so that the maximum of the first absorption matches with the one observed experimentally.

Transitions involving mostly the frontier orbitals described above are located in the 400–500 nm region. The transitions with higher oscillator strength are MLCT transitions from the d_*xz*_ orbital of the Fe^II^ center to both the isoquinoline and pyridine moieties in the lower energy band (S_6_, *f* = 0.2326) and only to the isoquinoline in the higher energy band (S_11_, *f* = 0.1375). As the acceptor orbital is spread through an extensive π system, it increases the transition dipole moment (proportional to the oscillator strength), and the intensity of this transition is therefore shifted to higher values than usually found for Fe^II^ -pyridine systems. The remaining transitions in this region are also MLCT transitions but with a small contribution of *d–d* transitions involving the Fe^II^ center. Figure 7 shows a fit of the calculated transitions in the 400–500 nm region to the observed spectrum.

For comparison, the same TD-DFT analysis was also performed for the analogous quinoline-based complex [Fe^II^(N2Py2Qn)(CH_3_CN)]^2+^. The frontier orbitals are roughly equivalent to those found for complex **4a** (*cf*. Figure S20). For [Fe^II^(N2Py2Qn)(CH_3_CN)]^2+^, two prominent bands that are similar to those of **1a**/**2a** are observed. The first, at lower energy, is mainly related to MLCT from the Fe^II^ center to the quinoline moieties. In this case, both LUMO and LUMO+1 are centered on the quinoline moieties. Therefore, the transition is not spread through an extensive π system, and lower oscillator strength values are observed for these transitions than that for the isoquinoline complex. The second absorption band is mainly an MLCT transition from the Fe^II^ center to the pyridine moieties.

#### Crystal and Molecular Structures of Complexes **1a**·(OTf)_2_, **2a**·(ClO_4_)_2_, **3a**·(OTf)_2_, **4a**·(BF_4_)_2_, and **5**·(OTf)_2._

**Figure 8:**
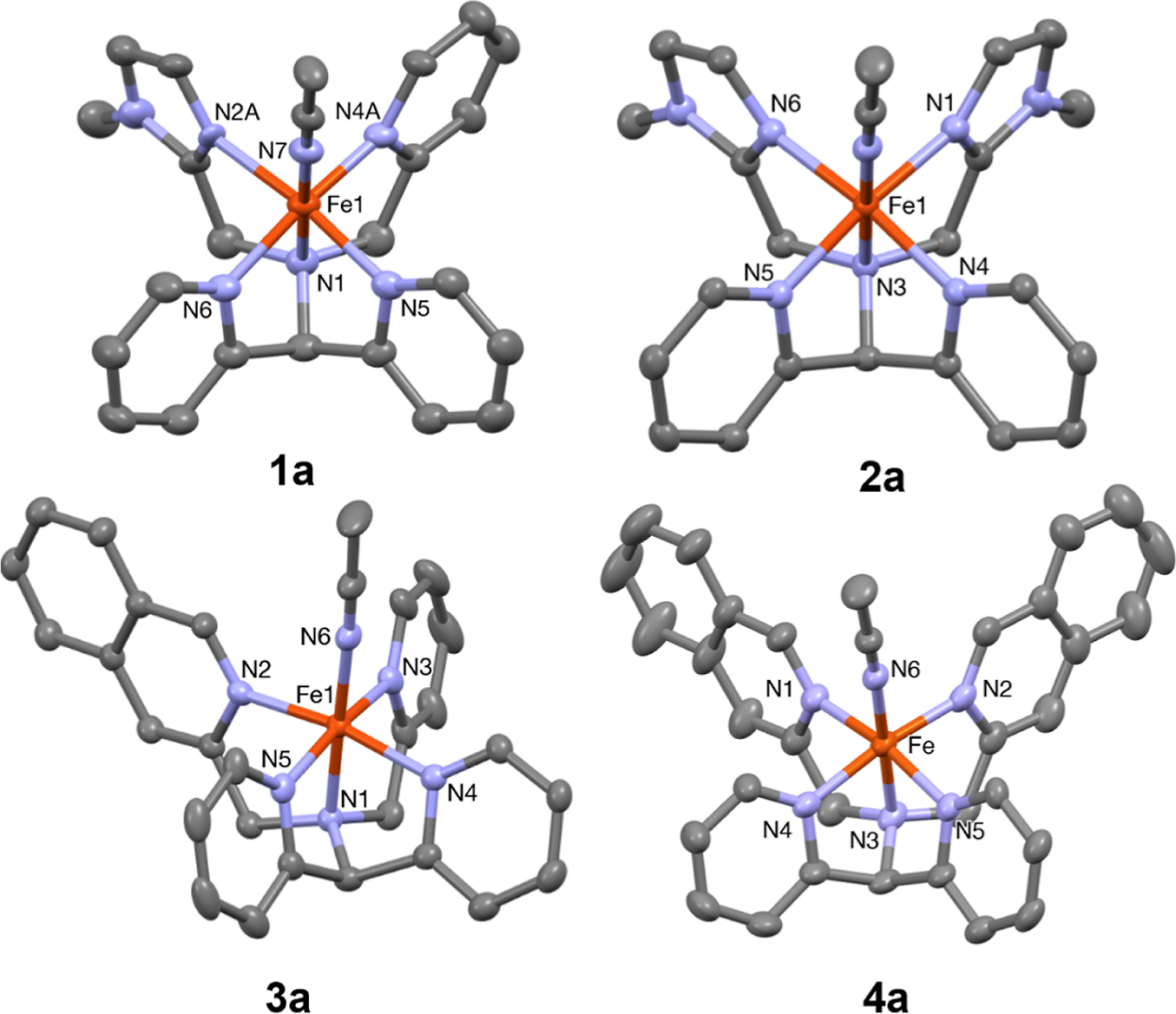
Mercury plots of the molecular structures of the cations of **1a**·(OTf)_2_, **2a**·(ClO_4_)_2_, **3a**·(OTf)_2_, and **4a**·(BF_4_)_2_ showing the atom labeling scheme. Thermal ellipsoids are plotted at 30% probability; hydrogen atoms have been omitted for clarity.

It was possible to grow single crystals suitable for X-ray diffraction of the perchlorate salt of **2a**, trifluoromethanesulfonate salts of **1a**, **3a**, and **5**, and tetrafluoroborate salt of **4a**, and their crystal structures were determined (Tables S2–S6 and S8). The molecular structures of complexes **1a**–**4a** are shown in Figure 8, and selected bond distances and bond angles are collated in Table 2. The crystal structures are similar to that of the “parent” complex [Fe^II^(N4Py)(CH_3_CN)](ClO_4_)_2_^30^ and show that the pentadentate ligands coordinate as envisaged, with the sixth coordination site at the iron ion being occupied by an acetonitrile molecule (solvent).

**Table 2:** Selected Bond Distances (Å) and Bond Angles (deg) for Fe^II^ Complexes **1a**–**4a**, [Fe^II^(N2Py2B)(CH_3_CN)](ClO_4_)_2_,^26^ [Fe^II^(N4Py)(CH_3_CN)](ClO_4_)_2_,^30^ and [Fe^II^(N2Py2Qn)(CH_3_CN)](OTf)_2_^28,32^^a^

	**1a**	**2a**	**3a**	**4a**	[Fe^II^(N4Py) (CH_3_CN)]^2+^	[Fe^II^(N2Py2B) (CH_3_CN)]^2+^	[Fe^II^(N2Py2Qn) (CH_3_CN)]^2+^
Fe–N (Å)	2.049(18) N2A	1.966(3) N1	1.963(2) N2	1.979(6) N1	1.976(3) N2	1.964(3) N1	2.103(5) N2
	2.039(19) N2B	1.967(3) N1B					
Fe–N (Å)	1.875(19) N4A	1.956(2) N4	1.964(3) N3	1.971(5) N2	1.967(3) N3	1.983(3) N3	2.092(5) N3
	1.87(2) N4B	1.959(2) N4B					
Fe–N (Å)	1.967(3) N5	1.957(2) N5	1.970(2) N4	1.990(5) N4	1.968(3) N4	1.979(2) N4	2.019(5) N4
		1.962(3) N5B					
Fe–N (Å)	1.964(3) N6	1.963(2) N6	1.953(3) N5	1.980(5) N5	1.975(3) N5	1.983(3) N2	2.010 (5) N5
		1.968(3) N6B					
Fe–NCH3CN(Å)	1.924(3) N7	1.920(2) N8	1.919(3) N6	1.942(6) N6	1.915(3) N6	1.901(3) N6	1.951(6) N1
		1.920(2) N8B					
Fe–N_amine_ (Å)	1.987(3) N1	2.013(2) N3	1.965(2) N1	1.982(5) N3	1.961(3) N1	2.028(3) N5	2.017(5) N6
		2.014(2) N3B					
Fe–N_equatorial_ (avg.) (Å)	1.964 A	1.9605	1.9625	1.980(7)	1.971	1.977	2.056
	1.96 B	1.964 B					
Fe–N_pyridine_ (avg.) (Å)	1.935 A	1.9565	1.962	1.980(8)	1.971	1.974	2.0145
	1.934 B	1.9605 B					
Fe–N_NMI/IQ/NMB/Qn_ (avg.)	2.049(18)	1.9645	1.963(2)	1.975(6)		1.981	2.0975
	2.039(19)	1.9675 B					
Angle_amine-Fe-CH3CN_ (deg)	179.16(12)	179.48(10)	177.74(11)	176.7(2)	177.3	175.7	166.7
		179.05(11)					

a NMI: (*N*-methyl)imidazolyl moiety. IQ: *N*-(isoquinolin-3-ylmethyl) moiety.

The Fe–N bond lengths of the equatorial plane observed for the complexes vary in the range of 1.9–2.0 Å, which is in agreement with those observed for [Fe^II^(N4Py)(CH_3_CN)](ClO_4_)_2_^30^ and some previously reported related Fe^II^ complexes.^17,18^ There are very slight shortenings of the Fe–N bond distances in the equatorial plane by approximately 0.01 Å for both **1**·(ClO_4_)_2_ and **2**·(ClO_4_)_2_ relative to the parent complex, while the axial Fe–N bond lengths are slightly longer than those for [Fe^II^(N4Py)(CH_3_CN)](ClO_4_)_2_. The replacement of two pyridyl moieties with (*N*-methyl)imidazolyl moieties in **2a**·(ClO_4_)_2_ results in a lengthening of the Fe–N_amine_ bond distance, *trans* to the acetonitrile ligand, by approximately 0.05 Å. Similar axial elongations have been observed for the Mn(II) analogue of **2a**(31) and for the complex [Fe^II^(N2Py2B)(CH_3_CN)]^2+26^ (*cf*. Figure 1 and Table 2). The tilt angles for the axial positions (N_amine_–Fe–N_CH3CN_) are small for all four complexes — **4a**·(BF_4_)_2_ (3.30°) > [Fe^II^(N4Py)(CH_3_CN)](ClO_4_)_2_ (2.68°) > **3a**·(OTf)_2_ (2.26°) > **1a**·(OTf)_2_ (0.84°) > **2a**·(ClO_4_)_2_ (0.52° or 0.95°) — indicating very little steric interaction between the equatorial donor groups and the axial acetonitrile ligands. A comparison between different Fe–N bond distances in **1a**–**4a**, [Fe^II^(N2Py2B)(CH_3_CN)](ClO_4_)_2_,^26^ [Fe^II^(N4Py)(CH_3_CN)](ClO_4_)_2_, and [Fe^II^(N2Py2Qn)](OTf)_2_ is shown in Table 2.

#### Cyclic Voltammetry

**Figure 9:**
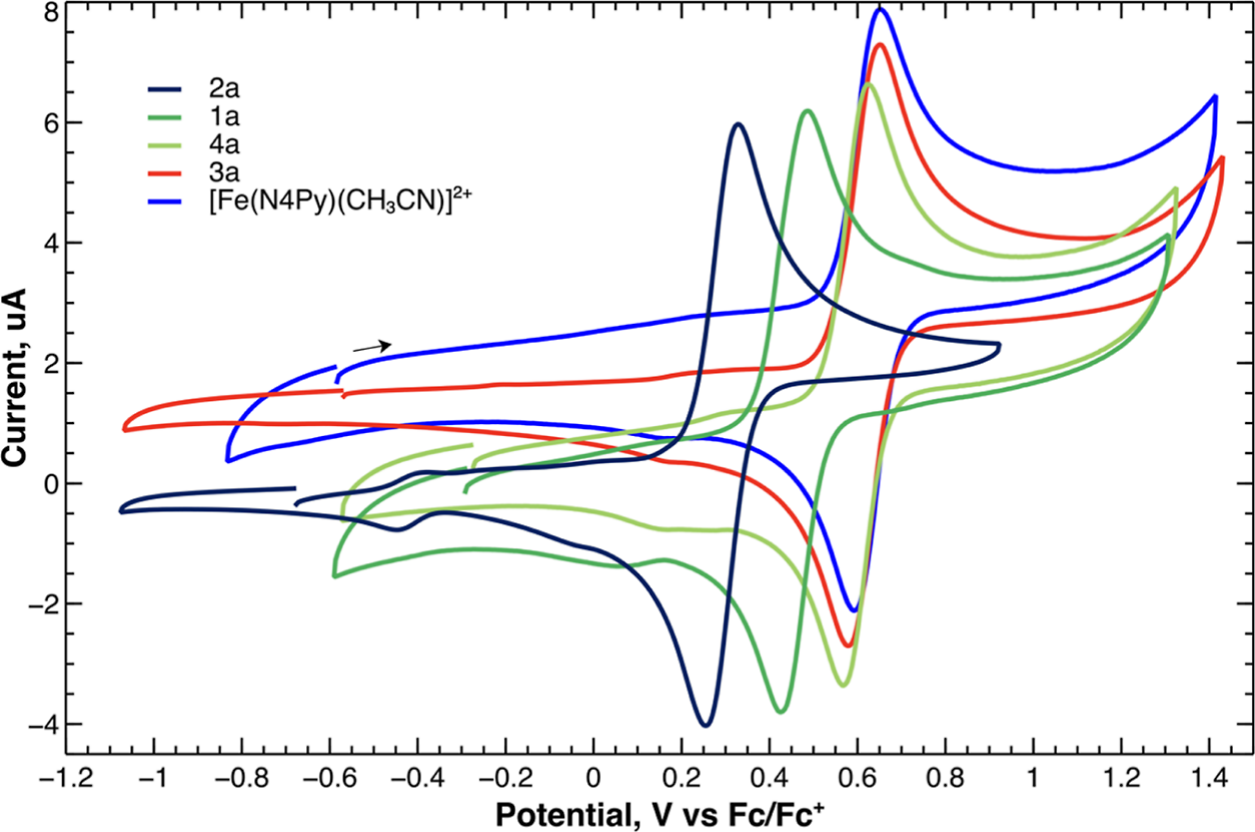
Cyclic voltammograms of **1a**–**4a** and [Fe(N4Py)(CH_3_CN)]^2+^ in acetonitrile at 50 mV/s *vs* Fc/Fc^+^.

Cyclic voltammetry (CV) in acetonitrile was used to measure the half-wave potentials (*E*_1/2_) for the Fe^II^ complexes. The cyclic voltammograms for **1a**–**4a** display a single reversible one-electron oxidation wave with peak separations comparable to that of ferrocene added to the same solution (Table 3 and Figure 9). Peak heights are linearly dependent on the square root of the scan rate examined from 5 to 3200 mV/s, as expected for Nernstian behavior (Figures S21–S24 in the Supporting Information.) The peaks are best assigned to the Fe^III^/Fe^II^ redox couple. Irreversible reduction waves were observed below −2.0 V for all complexes (Figure S26.) Scanning negative produced new small oxidation waves below 0 V even if no reduction peak was observed (See Figure S27 for an example.)

**Table 3:** CV Data for Complexes **1a**–**4a** and [Fe^II^(N4Py)(CH_3_CN)]^2+^

complex	*E*_1/2_ (V)^a^	Δ*E* (V) at scan rate of 100 mV/s^b^
**1a**	0.452	0.063
**2a**	0.298	0.085
**3a**	0.609	0.075
**4a**	0.596	0.067
[Fe^II^(N4Py)(CH_3_CN)]^2+^	0.625	0.070
Fc/Fc^+^	0	0.087

a Voltages measured relative to the ferrocene ([Fe(C_5_H_5_)_2_])/ferrocenium ([Fe(C_5_H_5_)_2_]^+^) redox couple (Fc/Fc^+^). No difference was observed between Pt and glassy carbon working electrodes.

b Observed Δ*E* in 0.10 M (Bu_4_N)PF_6_/CH_3_CN at room temperature, indicating that one electron is reversibly transferred.

The *E*_1/2_ of the complexes become more positive in the following sequence: **2a** < **1a** < **4a** ≤ **3a** ≤ [Fe(N4Py)(CH_3_CN)]^2+^. These results demonstrate that successive replacement of the pyridyl moieties by (*N*-methyl)imidazolyl moieties leads to a significant lowering of the potentials for the oxidation of the corresponding Fe^II^ complexes, while substitution of pyridyl donor moieties with isoquinolines results in only very minor changes in the redox potentials. In addition, the order of potentials correlates well with the Fe–N_amine_ distances observed by crystallography, which are in the order **2a** > **1a** ≥ **4a** > **3a** ≥ [Fe(N4Py)(CH_3_CN)]^2+^. In general, strong ligand fields result in higher redox potential values for the Fe^III^/Fe^II^ redox couple. The correlation suggests that the Fe–N_amine_ distance may be used as an indicator of the ligand field strength in these complexes. As discussed above, **2a** is expected to be in a mixture of high- and low-spin states in the solid state, and its longer Fe–N_amine_ distance may reflect the population of the d_*z*_^2^ orbital in the high-spin form; the Fe–N_amine_ distance of **2a** is ≈ 2.01 Å, while that of the other three (low-spin) complexes is approximately 1.97–1.98 Å.

### Generation and Characterization of Fe^IV^=O Complexes

**Figure 10:**
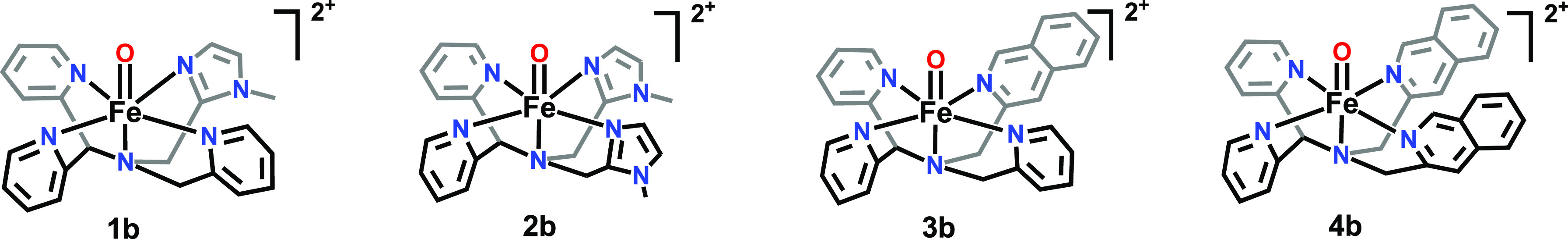
Schematic drawings of Fe^IV^=O complexes **1b**–**4b**.

Upon addition of three equivalents of IBX ester as an oxidant, the reddish-brown solutions of the Fe^II^ complexes **1a**–**4a** turned pale green (cf. graphical abstract) instantaneously, which is in agreement with the expected color of the corresponding Fe^IV^=O complexes **1b**–**4b** (Figure 10), by analogy with previous studies on Fe^IV^=O complexes based on the N4Py ligand scaffold.^24,25^ High-resolution mass spectrometry (HRMS) analyses of the pale green solutions confirmed the formation of the Fe^IV^=O complexes. Both [Fe^IV^=O(**L**^***n***^)X]^+^ and [Fe^IV^=O(**L**^***n***^)]^2+^ (*n* = 1–4, X = ClO_4_^–^ or OTf^–^) peaks were observed (*cf*. the Experimental Section and Figures S36–S39, Supporting Information).

Fe^IV^=O complexes **1b**–**4b** could also be prepared by treating concentrated CH_3_CN solutions of the corresponding Fe^II^ precursors with 4 equiv of ceric ammonium nitrate as the oxidant, in water. Subsequent addition of excess sodium perchlorate to the reaction mixtures resulted in precipitation of the solid ferryl complexes in the case of **3b** and **4b**.

#### Mössbauer Spectroscopy and Magnetic Measurements on **1b**–**4b**

**Figure 11:**
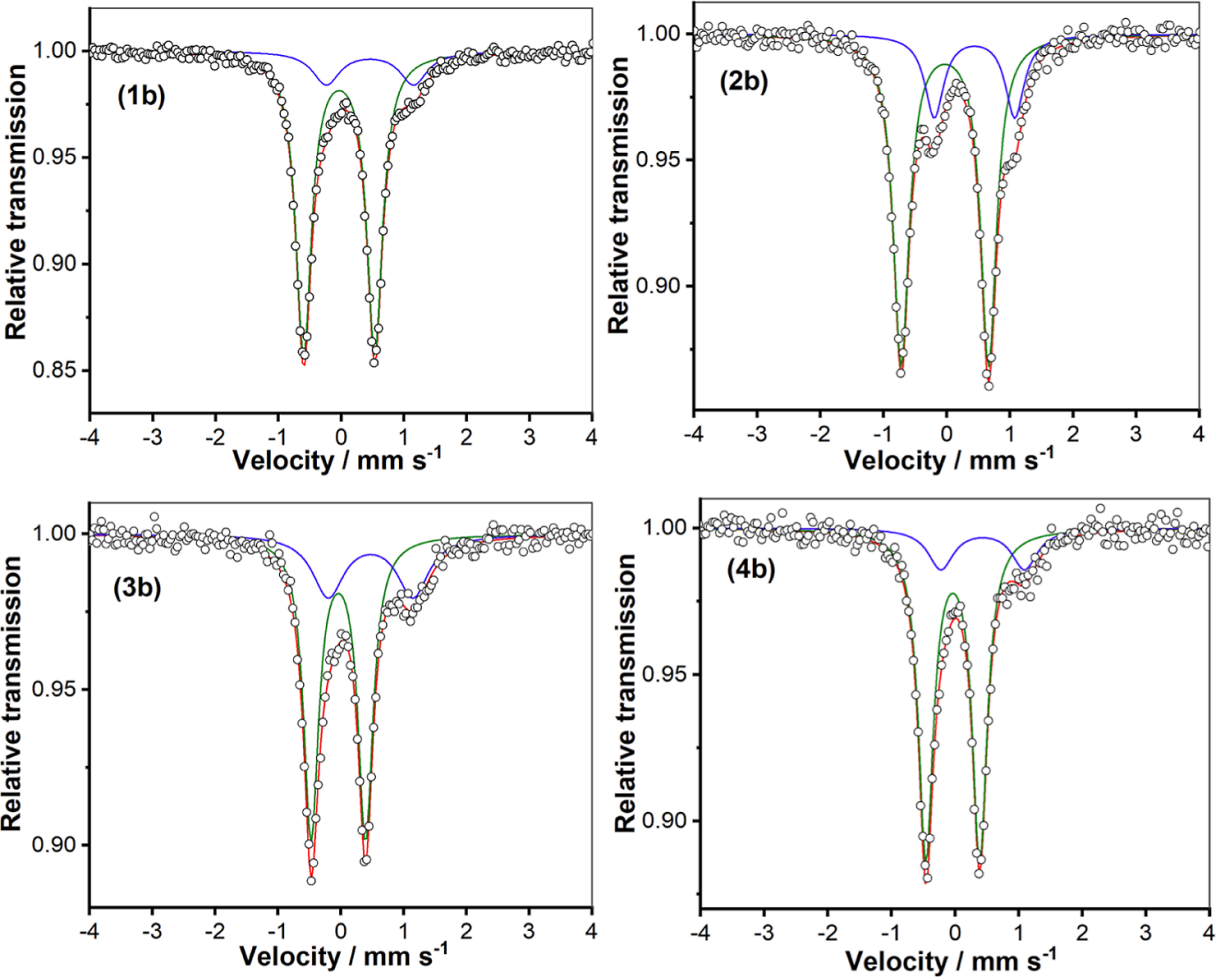
Zero-field Mössbauer spectra of Fe^IV^=O complexes **1b**–**4b** in frozen acetonitrile at 80 K.

Mössbauer spectroscopy was employed to confirm the oxidation states of the *in situ* generated Fe^IV^=O complexes **1b**–**4b** (Figure 11). The isomer shift and quadrupole splitting values are given in Table 4. The Mössbauer parameters for **1b**–**4b** are very similar to those of previously studied low-spin Fe^IV^=O species,^11,25^ strongly indicating the presence of low-spin (*S* = 1) Fe^IV^=O units in all four complexes. The remaining (minor) absorptions are ascribed to Fe^III^ contaminants; their isomer shifts lie in the range 0.43–0.47 mm s^–1^ with quadropole splittings of 1.27–1.39 mm s^–1^—parameters that are typical for high-spin ferric complexes. These contaminants may be (high-spin) Fe^III^–O–Fe^III^ complexes,^33,34^ such as complex **5**.

**Table 4:** Mössbauer Parameters for *In Situ* Generated Complexes **1b**–**4b** and Related Fe^IV^=O Complexes

complex	δ (mm s^–^^1^)	Δ*E*_Q_ (mm s^–^^1^)
**1b** (80 K), 84%	–0.03	1.13
**2b** (80 K), 77%	–0.03	1.38
**3b** (80 K), 70%	–0.04	0.87
**4b** (80 K), 83%	–0.04	0.85
[Fe^IV^(O)(N4Py)]^2+^^35^	–0.04	0.93
[Fe^IV^(O)(N2Py2B)]^2+^^26^	–0.02	1.34
[Fe^IV^(O)(N3Py-(NMB))]^2+^^26^	–0.03	1.1
[Fe^IV^(O)(TMC) (CH_3_CN)]^2+^^36^	0.14	0.78
[Fe^IV^(O)(Bn-tpen)]^2+^^35^	0.01	0.87
[Fe^IV^(O)(N2Py2Qn)]^2+^^32^	0.03	0.56

Magnetic measurements at ambient temperature using the Evans method were performed on the four Fe^IV^=O complexes **1b**–**4b** in acetonitrile solution, and the results indicated that all four complexes are in the low-spin (*S* = 1) state (*cf*. Figures S40–S42, Supporting Information). In addition, SQUID magnetic susceptibility measurements in the temperature range 80–295 K on solid samples of **3b**·(OTf)_2_ and **4b**·(OTf)_2_ gave χ_M_*T* values of ca. 0.76 and 0.92 cm^3^ mol^–1^K, respectively, slightly lower than that expected for the spin-only value of 1.0 cm^3^ mol^–1^K for low-spin (*S* = 1) Fe^IV^=O complexes (Figure S28, Supporting Information). When the temperature was decreased below 80 K, the χ_M_*T* value was found to decrease slowly due to the presence of zero-field splitting. Simulation of the magnetic susceptibility data and variable-temperature variable-field measurements according to the spin Hamiltonian in eq 1 leads to the best fit parameters *g* = 1.77, *D* = 16.6 cm^–1^ for **3b**·(OTf)_2_ and *g* = 1.95, *D* = 20.9 cm^–1^ for **4b**·(OTf)_2_ (see Supporting Information for more details); the low χ_M_*T* and *g* values should be considered with caution, as they may reflect the presence of diamagnetic impurities.
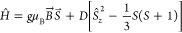
1

**Figure 12:**
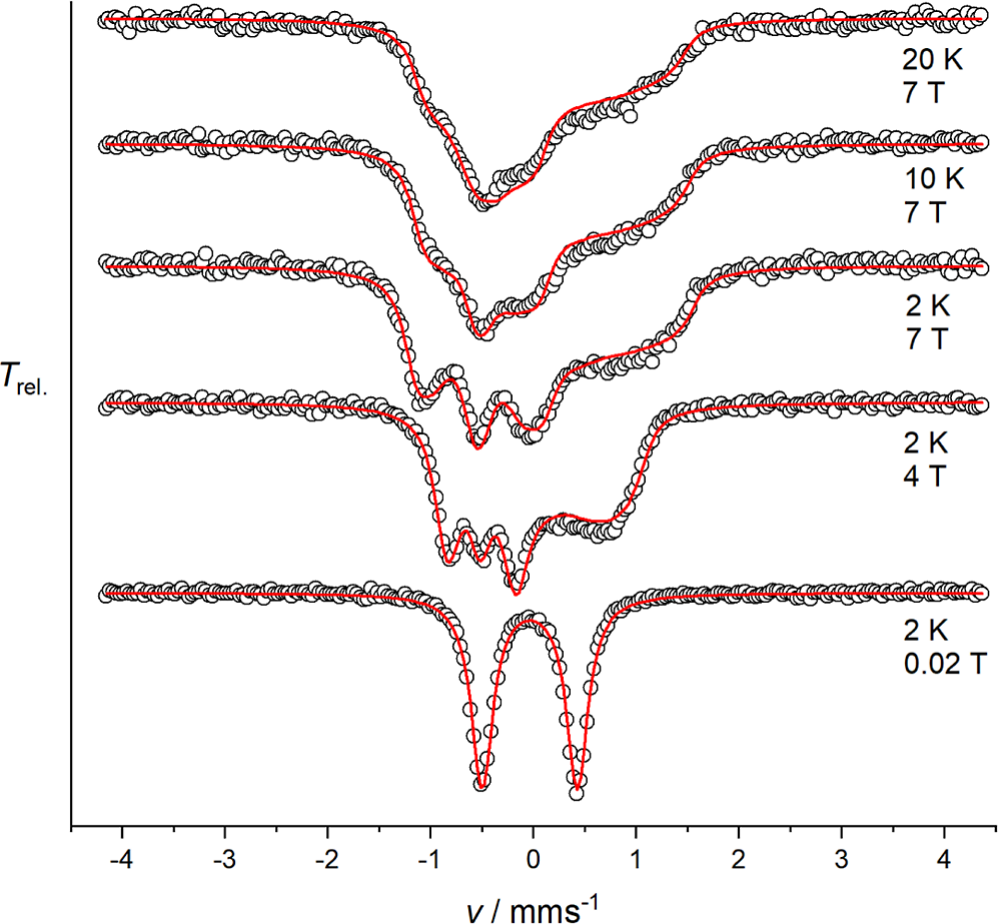
Magnetic Mössbauer spectra of solid **4b**·(OTf)_2_ recorded at the indicated temperatures and fields applied perpendicular to the γ-rays. The red lines represent the best fit to a spin Hamiltonian for *S* = 1 in the fast spin relaxation regime.

To gain more insight into the electronic structure and to verify the *D* value by an independent method, solid **4b**·(OTf)_2_ was investigated by applied magnetic field Mössbauer spectroscopy between 2 and 20 K and up to 7 T magnetic field (Figure 12).

The magnetically split Mössbauer spectra of **4b**·(OTf)_2_ could be well simulated using the spin Hamiltonian for *S* = 1 with fit parameters *D* = 20.8 cm^–1^ (*g* = 1.95, fixed from magnetic susceptibility data), the magnetic hyperfine coupling constants *A*/*g*_N_μ_N_ = [−19.8, −16.8, −2.5] T, as well as δ = −0.04 mm s^–1^ and Δ*E*_Q_ = 0.92 mm s^–1^. Therefore, the sign and the size of the zero-field splitting parameter *D* are in excellent agreement with those derived from the magnetic susceptibility data. Two large negative values and one small negative value of *A*/*g*_N_μ_N_ are in accordance with results previously reported for a tetragonal *S* = 1 Fe^IV^=O species.^37^

#### Absorption Spectroscopy on Fe^IV^=O Complexes

**Figure 13:**
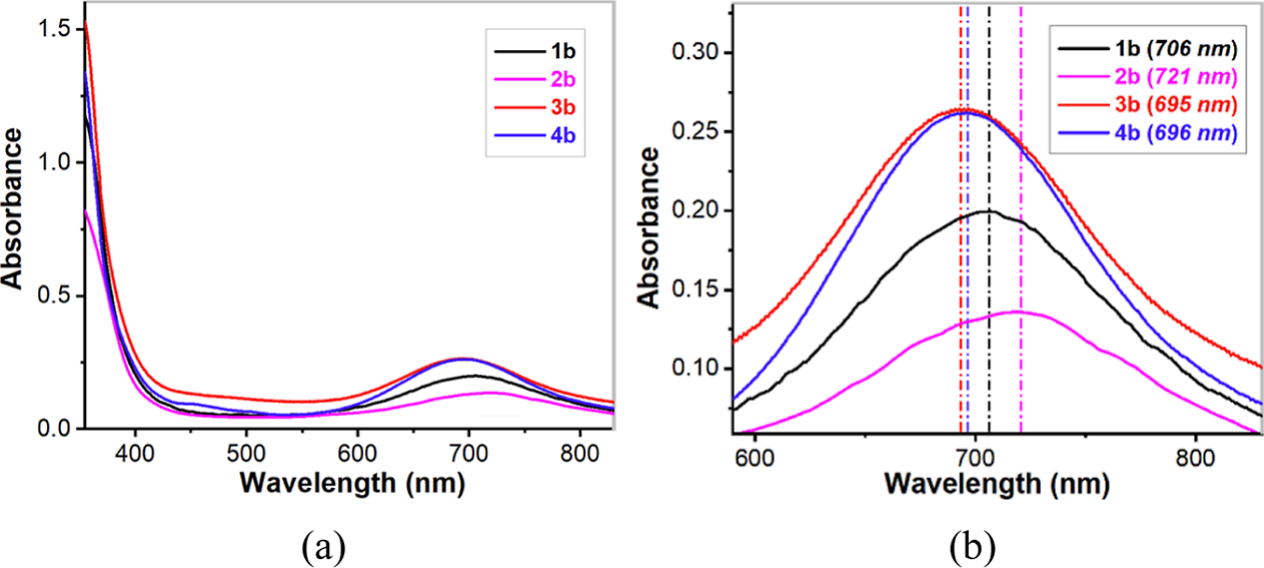
(a) UV/vis spectra of complexes **1b**, **2b**, **3b,** and **4b** (0.5 mM) in acetonitrile recorded at room temperature (298 K). (b) Expansion of the 600–800 nm region of the spectra.

**Figure 14:**
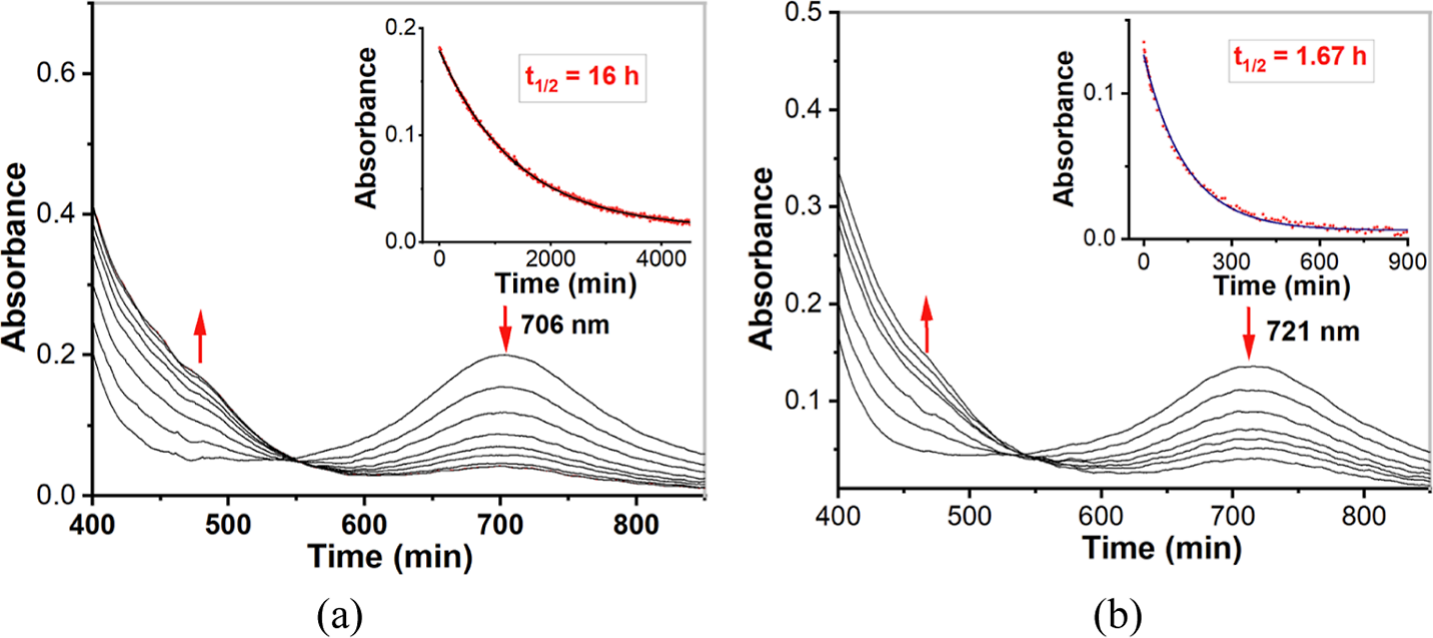
(a) UV/vis spectra of 0.5 mM complex **1b**·(ClO_4_)_2_ in acetonitrile. The inset shows the self-decay of complex **1b**·(ClO_4_)_2_ at room temperature (298 K), as monitored at 706 nm; (b) UV/vis spectra of 0.5 mM complex **2b**·(ClO_4_)_2_ in acetonitrile. The inset shows the self-decay of complex **2b**·(ClO_4_)_2_ at room temperature (298 K), as monitored at 721 nm.

The absorption spectra of Fe^II^ complexes **1a**–**4a** have been discussed above. The UV/vis spectra of pale green **1b**–**4b** resemble those of related Fe^IV^=O complexes of pentadentate nitrogen-donor ligands.^24,38^ In particular, a characteristic weak absorption with λ_max_ in the vicinity of 700 nm was observed for all four complexes (Figure 13). This absorption in the near-IR region has been studied in detail by Solomon, Que and co-workers for the parent complex [Fe^IV^(O)(N4Py)]^2+^,^39^ and it is related to transitions originating from the occupied d_*xy*_ orbital and constitutes an indirect measurement of the strength of the equatorial ligand field. The time courses for decay of the Fe^IV^=O complexes were also monitored at 298 K (Figure 14). As calculated, complex **1b** has a *t*_1/2_ ≈ 16 h; *t*_1/2_ (**2b**) ≈1.67 h; *t*_1/2_ (**3b**) ≈ 63 h; *t*_1/2_ (**4b**) ≈ 45 h. In Table 5, the half-lives and the characteristic near-IR wavelengths of these four new Fe^IV^=O complexes are compared to some previously reported low-spin Fe^IV^=O complexes bearing pentadentate ligands.

**Table 5:** Optical Spectral Properties and Stabilities (*t*_1/2_) of Fe^IV^=O Complexes Supported by Pentadentate Ligands

complex	λ_max,_ nm (ε, M^–^^1^ cm^–^^1^)	*t*_1/2_ at 298 K	ref.
**1b**	706 (379)	16 h	this work
**2b**	721 (274)	1.67 h	this work
**3b**	695 (540)	63 h	this work
**4b**	696 (544)	45 h	this work
[Fe^IV^(O)(N4Py)]^2+^	696 (400)	60 h	(35)
[Fe^IV^(O)(Me_3_cyclam-CH_2_C(O)NMe_2_)]^2+^	810 (270)	5 d	(40)
[Fe^IV^(O)(N_2_Py2B]^2+^	725 (380)	2.5 h	(26)
[Fe^IV^(O)(Bn-tpen)]^2+^	739 (400)	6 h	(35)
[Fe^IV^(O)(TMC-Py)]^2+^	834 (260)	7 h	(41)
[Fe^IV^(O)(N2Py2Qn]^2+^	770 (380)	2.5 h	(25)
[Fe^IV^(O)(N_4_Py^Me2^)]^2+^	740 (220)	14 min	(29)

As shown in Table 5, both the sequence of λ_max_ in the near IR region −721 nm (**2b**) > 706 nm (**1b**) > 696 nm (**4b**) > 695 nm (**3b**) = 695 nm ([Fe^IV^(O)(N4Py)]^2+^) and the sequence of half-lives at room temperature −1.67 h (**2b**) < 16 h (**1b**) < 45 h (**4b**) < 63 h (**3b**) ≈ 60 h ([Fe^IV^(O)(N4Py)]^2+^) indicate that the ligand fields exerted by the isoquinoline-substituted ligands **L**^**3**^ and **L**^**4**^ are roughly equivalent to that of N4Py, while the fields of **L**^**1**^ and **L**^**2**^ tend to become progressively weaker as (*N*-methyl)imidazolyl substituents are introduced in the N4Py ligand scaffold, and are significantly weaker than those of the other two ligands.^23^

#### Crystal and Molecular Structure of [Fe^IV^(O)(L^4^)]^2+^

**Figure 15:**
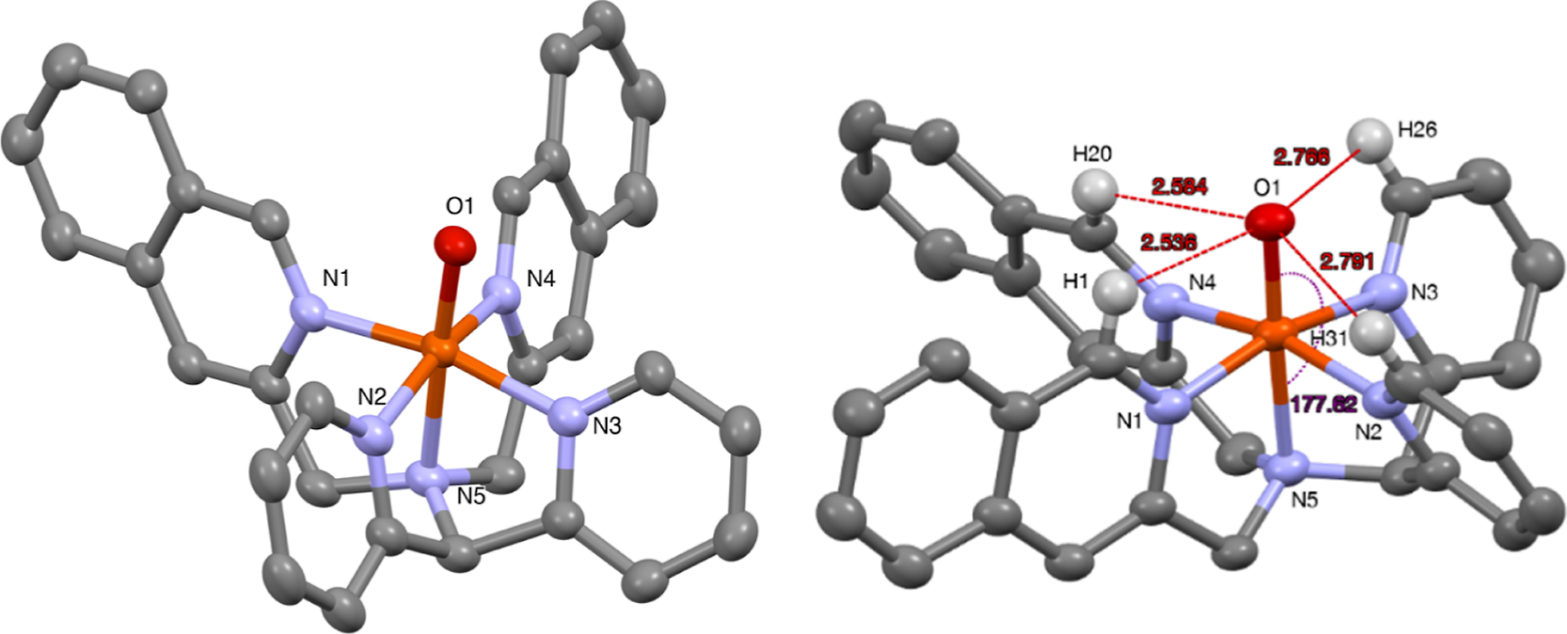
Mercury plots of the complex cation of **4b** with 40% probability ellipsoids in two perspectives. Hydrogen atoms have been omitted for clarity with the exception of ligand hydrogens in direct vicinity of the oxido ligand (right-hand plot). Selected bond distances (Å) and angles (deg): Fe(1)–O(1) 1.6584(14), Fe(1)–N(1) 1.9546(17), Fe(1)–N(2) 1.9685(17), Fe(1)–N(3) 1.9709(17), Fe(1)–N(4) 1.9608(17), Fe(1)–N(5) 2.0486(16), N(4)–Fe(1)–N(2) 164.78(7), O(1)–Fe(1)–N(5) 177.61(7).

Crystals of **4b** suitable for X-ray analysis could be obtained by slow evaporation of acetonitrile at 2–5 °C over the course of 8 h,^25^ and diffraction data were obtained at the MAX IV synchrotron (*cf*. the Experimental Section). Relevant crystallographic details are summarized in Table S2 (Supporting Information). As shown in Figure 15, the axial positions are occupied by the amine nitrogen (N5) and the oxygen atom (O1) with an almost linear N5–Fe1–O1 angle of 177.61(7)°. The tilt angle (2.4°) is much less than that observed in the Fe^IV^=O complexes of the analogous N2Py2Qn ligand but is very similar to that for the parent N4Py complex. The Fe1–O1 bond distance of 1.6584(14) Å is very close to that reported for other crystallographically characterized *S* = 1 Fe^IV^=O complexes. As expected, the steric influence of the ligand moieties in **4b** is minimal, and the effective ligand field is therefore expected to primarily be related to the electronic properties of the various N-donor entities. A comparison of bond lengths and tilt angles, and the relation to the reactivities for **4b** and other related complexes, are listed in Table 8 (*vide infra*).

### HAT Reactions

The observed short lifetimes of complexes **1b** and **2b** indicate that the complexes may exhibit enhanced reactivity toward external substrates in comparison to the parent [Fe^IV^(O)(N4Py)]^2+^ complex. A series of alkane and arene substrates having different C–H bond dissociation energies (BDEs), ranging from 81 to 99.3 kcal/mol, were studied, and the relative reactivities of complexes **1b**–**4b** were evaluated. The reactions showed pseudo-first-order behavior under conditions of excess substrate (10–1000 equiv), and the observed rate constants (*k*_obs_) were linearly dependent on substrate concentration. From these linear plots, second-order rate constants (*k*_2_) were obtained. The *k*_2_ values thus obtained are listed in Table 6. A primary kinetic isotope effect (KIE) value of ∼14 for HAT by **2b** was obtained by determining the second-order rate constants for separate reactions of toluene and its d_8_ isotopomer with the Fe^IV^=O complex (Figure S29), using the same methodology described above; this KIE value is quite similar to that obtained for the (*N*-methyl)benzimidazolyl equivalent of **2b**.^26^

**Table 6:** Comparison of Second-Order Rate Constants for HAT Reactions Affected by Complexes **1b**–**4b** and [Fe^IV^(O)(N4Py)]^2+^^a^

alkane^b^	D_C__–__H_^c^(kal/mol)	*k*_2_^d^ (M^–^^1^ s^–^^1^) for **1b**	*k*_2_^d^ (M^–^^1^ s^–^^1^) for **2b**	*k*_2_^d^ (M^–^^1^ s^–^^1^) for **3b**	*k*_2_^d^ (M^–^^1^ s^–^^1^) for **4b**	*k*_2_^d^ (M^–^^1^ s^–^^1^) for [Fe^IV^(O)N4Py]^2+^
triphenylmethane (1)	81	8.2 × 10^–2^	8.43 × 10^–1^	6.05 × 10^–2^	1.23 × 10^–1^	0.037
cumene (1)	84.5	3.56 × 10^–^^3^	2.13 × 10^–2^	3.0 × 10^–^^3^	3.6 × 10^–^^3^	2 × 10^–^^3^
ethylbenzene (2)	87	2.66 × 10^–^^3^	1.84 × 10^–2^	1.9 × 10^–^^3^	3.1 × 10^–^^3^	4 × 10^–^^3^
toluene (3)	90	5.78 × 10^–^^4^	4.7 × 10^–^^3^	3.3 × 10^–^^4^	4.5 × 10^–^^4^	6.3 × 10^–^^4^
cyclooctane (16)	95.3	2 × 10^–^^3^	1.2 × 10^–2^	3 × 10^–^^4^	6.7 × 10^–^^6^	
2,3-dimethylbutane (2)	96.5			2.3 × 10^–^^4^		1.2 × 10^–^^4^
cyclohexane (12)	99.3	3.53 × 10^–^^4^	4.33 × 10^–^^3^	9.14 × 10^–^^5^	6.7 × 10^–^^6^	5.5 × 10^–^^5^
self-decay		1.17 × 10^–^^5^	1.17 × 10^–^^4^	2.8 × 10^–^^6^	4.3 × 10^–^^6^	2.3 × 10^–^^6^

a All experiments were performed in triplicate, at a minimum.

b Numbers in parentheses represent the number of H atoms on the substrate that would react with the Fe^IV^=O species.

c D_C–H_ values were obtained from ref (29).

d The *k*_2_’ values used in Figure 16 were obtained by dividing *k*_2_ values listed here by the number of equivalent H atoms on the substrate listed under the alkane substrate column.

A plot of logarithmic values of second-order rate constants (log *k*_2_’) (*k*_2_’ is the second-order rate constant divided by the number of equivalent C–H bonds in the substrate) for absorbance decay versus C–H BDEs for the different substrates shows a roughly linear correlation (Figure 16). This correlation and the observed large KIE value for **2b** constitute strong evidence of the reactions taking place *via* HAT.

**Figure 16:**
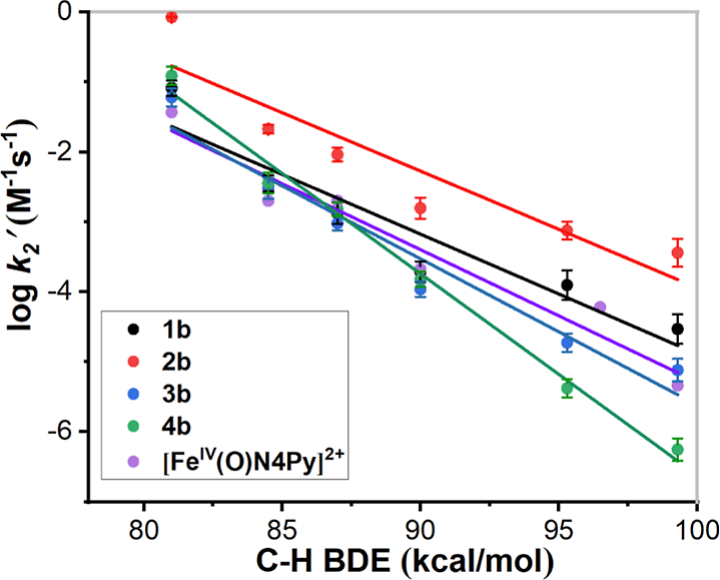
Plots of log *k*_2_’ versus C–H BDEs of different alkane substrates for **1b**, **2b**, **3b**, **4b**, and [Fe^IV^(O)(N4Py)]^2+^; see Table 6 for the identities of the substrates.

Complexes **1b** and **2b** reacted readily with the C–H bonds of the different substrates at ambient temperature. On the other hand, for substrates with high BDEs, **3b** and **4b** reacted at slightly lower rates than [Fe^IV^(O)(N4Py)]^2+^. The plots in Figure 16 clearly show that **2b** consistently reacted with faster rates of reaction than the other ferryl complexes. The approximate collinearity of the plots/slopes for complexes **1b** and **2b** indicates that the effect on reaction rates upon introducing the second (*N*-methyl)imidazolyl arm is roughly the same irrespective of the substrate. At higher substrate BDEs—95.3 kcal/mol (cyclooctane), 99.3 kcal/mol (cyclohexane)—the HAT rates for the four different Fe^IV^=O complexes are clearly differentiated, and the order is **2b** > **1b** > **3b** > **4b** in the order of decreasing HAT rates. Given that the steric influence of the ligands appears to be low, as reflected by the N_amine_–Fe–N_CH3CN_ angles in **1a**–**4a** (Table 2, *vide supra*), we interpret the differences in the reactivities of the Fe^IV^=O complexes to be mainly attributable to the electronic (donor) properties of the various N-donor entities of the four ligands. It may be noted that while **4b** displays the lowest reactivity of all Fe^IV^=O complexes, the observed rate constant for this complex is on par with those of both **1b** and **3b** for HAT from triphenylmethane, which is the substrate with the lowest BDE but the greatest steric bulk around the C–H bond; **2b** remains the most reactive species also for this substrate. Again, this suggests that it is the electronic contributions of the ligand donor moieties that mainly dictate the reactivities of the complexes rather than the steric influence of the ligands.

### OAT Reactions

**Scheme 2:**
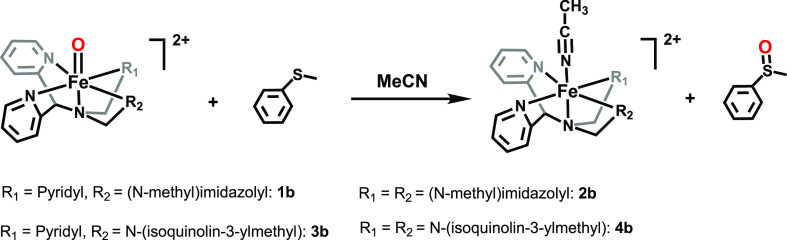
OAT to Thioanisole by Complexes **1b**–**4b**

**Figure 17:**
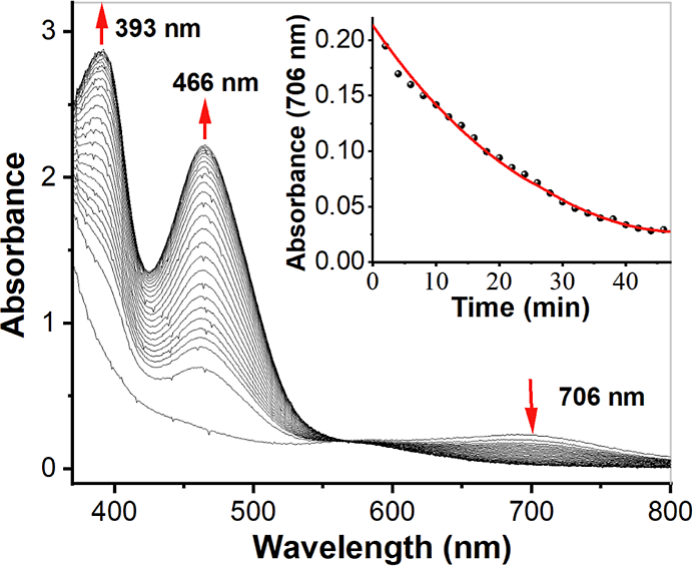
Decay of **1b** to its Fe^II^ precursor in the presence of thioanisole (250 mM) at 243 K. Inset: time course of the decay monitored at 706 nm.

The OAT abilities of **1b**–**4b** were also investigated, using thioanisole (PhSMe) as a substrate (Scheme 2). Because of the rapid reaction rates, the reactivities of **1b** and **2b** were studied at 243 K. Upon the addition of thioanisole, the characteristic near-IR band (λ_max_ = 706 nm) decayed for complex **1b**, indicating OAT from the Fe^IV^=O complex to thioanisole to form methyl phenyl sulfoxide (*vide infra*). During the course of the reaction, complex **1b** was converted into its Fe^II^ precursor (**1a**) with a clear isosbestic point at 565 nm as identified by UV/vis spectrophotometry (Figure 17). The reaction showed pseudo-first order behavior under conditions of excess substrate (100–500 equiv) and the observed rate constant (*k*_obs_) was linearly dependent on substrate concentration (Figure S30). From this linear plot, a second-order rate constant (*k*_2_) with a value of (2.6 ± 0.2) × 10^–3^ M^–1^ s^–1^ was obtained for the oxidation of thioanisole by complex **1b**. Under the same conditions, **2b** reacted with thioanisole at a rate considerably faster than **1b**. The second-order rate constant (*k*_2_) for complex **2b** was found to be (4.5 ± 0.2) × 10^–1^ M^–1^ s^–1^, meaning that **2b** is more reactive by approximately two orders of magnitude than **1b** in this OAT reaction (Figure S30). Similarly, the OAT reactivities of **3b** and **4b** were analyzed at 273 K (Figure S31). Table 7 shows a comparison of the reaction rates of the OAT (thianisole oxidation) processes of complexes **1b**–**4b** with previously reported Fe^IV^=O complexes. It can be seen that complex **2b** is among the most reactive Fe^IV^=O complexes for OAT (thianisole oxidation) and approximately on par with its (*N*-methyl)benzimidazolyl analogue [Fe^IV^(O)(N_2_Py2B)]^2+^.^26^ Based on a previous study of a series of *S* = 1 Fe^IV^=O complexes, the rates of OAT to thioanisole correlate linearly with the increase in the redox potentials, reflecting the relative electrophilicities of the Fe^IV^=O complexes.^38^ It may thus be inferred that complex **2b** has much higher electrophilicity than complexes **1b, 3b, 4b**, [Fe^IV^(O)(N4Py)]^2+^, and most other reported iron-oxo species supported by mixed amine/pyridine donor pentadentate ligands.

**Table 7:** OAT (Thioanisole Oxidation) of Different Fe^IV^=O Complexes Supported by Different N5 Ligands

complex	*k*_2_ (M^–^^1^ s^–^^1^)	temperature of measurement (K)	ref.
**1b**	0.00026	243	this work
**2b**	0.45	243	this work
**3b**	0.068	273	this work
**4b**	0.034	273	this work
[Fe^IV^(O)(N4Py)]^2+^	0.014	263	(25)
[Fe^IV^(O)(N4Py)]^2+^	0.00024	233	(25)
[Fe^IV^(O)(Bn-tpen)]^2+^	0.33	263	(38)
[Fe^IV^(O)(Bn-tpen)]^2+^	0.014	233	(38)
[Fe^IV^(O)(N_2_Py2B)]^2+^	0.31	243	(26)
[Fe^IV^(O)(N_3_Py-(NMB))]^2+^	0.033	243	(26)
[Fe^IV^(O)(N2Py2Qn)]^2+^	7.4	233	(25,29)
[Fe^IV^(O)(N3Py-Qn)]^2+^	0.023	233	(28)
[Fe^IV^(O)(N_4_Py^Me2^)]^2+^	1.03	263	(29)
[Fe^IV^(O)(N4Py)]^2+^	0.049	273	(25)

Comparative parameters for a few structurally related ferryl complexes of N4Py and its derivatives are collated in Table 8. In contradiction to previous reports where a linear correlation has been proposed between the tilt angle and the reactivity,^25^ the reactivity patterns cannot be directly correlated with the tilt angle of the Fe–O moiety. For example, the tilt angle of **4b** is 2.4°, which lies between the Fe^IV^=O complexes of the N2Py2B and N4Py ligands. However, complex **4b** is much less reactive than the Fe^IV^=O complex of both N2Py2B and N4Py ligands in cyclohexane/thioanisole oxidation. Also, there is no correlation between the Fe=O bond lengths in the Fe^IV^=O complexes and their reactivities. Based on the discussion above, it remains difficult to adequately predict reactivity only depending on steric/structural criteria.

**Table 8:** Comparison of Bond Lengths and Fe^IV^=O Tilt Angles with Observed Rate Constants for **4b** and Related Complexes

ligand	**L**^4^	N4Py	N2Py2B	N2Py2Qn	N3Py-Qn
Fe=O (Å)	1.6584(14)	1.639(5)	1.656(4)	1.677(5)	1.642(5)
Fe–N_amine_ (Å)	2.0486(16)	2.033(8)	2.115(6)	2.084(4)	2.053(6)
Fe–N_Py_ (avg.) (Å)	1.9697(17)	1.964(5)	1.989	2.023	1.977
Fe–N_IQ/Py/NMB/Qn_ (avg.) (Å)^a^	1.9577(17)	1.949(5)	1.952	2.070	2.025(5)
Fe–N_equatorial_ (avg.)	1.9637(17)	1.9565(5)	1.9705	2.0465	1.989
tilt angle (deg)	2.4	0.6	3	9.57	5.8
*k*_2_ for thioanisole oxidation (M^–^^1^ s^–^^1^) (temp., K)	0.0345 (273 K)	0.049 (273 K)	0.31 (243 K)	7.4 (233 K)	0.023 (233 K)
*k*_2_ for cyclohexane oxidation (M^–^^1^ s^–^^1^) (298 K)	0.0067 × 10^–^^3^	0.055 × 10^–^^3^	2.9 × 10^–^^3^	30 × 10^–^^3^	0.86 × 10^–^^3^
ref.	this work	(25)	(26)	(29)	(28)

a IQ: *N*-(isoquinolin-3-ylmethyl) moiety.

### Catalytic Oxidation Reactions Effected by the Fe^II^ Complexes **1a**–**4a**

A series of aromatic and aliphatic substrates were used to analyze catalytic oxidations effected by **1a**–**4a**, using IBX ester or H_2_O_2_ as the terminal oxidants. The catalytic data are collected in Tables 9 and 10. For OAT catalysis with thioanisole, all Fe^IV^=O complexes in this work show excellent turnovers (yield ≈ 100%) with IBX ester. Similarly, for the HAT reaction with IBX ester as an oxidant, complexes **3a** and **4a** gave higher yields than **1a** or **2a** for substrates with high BDEs. Furthermore, complexes **3a** and **4a** could be recovered from the reaction mixtures after the completion of the catalytic reactions, but this was not the case for **1a** and **2a**. These observations may be rationalized by the established greater instability of the active species formed from **1a** and **2a**, which are presumed to be the corresponding ferryl complexes **1b** and **2b**. These unstable species may undergo degradation, leading to unproductive pathway(s) and low catalytic turnover. For the substrates toluene and cyclohexene, the total turnover numbers (all products) were less than 1 when IBX ester was used as an oxidant, and the reactions were thus not catalytic.

**Table 9:** Catalytic Activities of Complexes **1**–**4b** with IBX Ester (Catalyst:IBX Ester = 1:10)

substrate (mmol)	product	complex **1a**		complex **2a**		complex **3a**		complex **4a**	
		TON^a^	efficiency^b^	TON	efficiency	TON	efficiency	TON	efficiency
thioanisole (0.25)	thioanisole oxide	9.2	92	10	100	10	100	10	100
9,10-DHA (0.05)	anthracene	9.6	96	10	100	10	100	9.6	96
1,4-CHD (0.25)	benzene	9.3	93	9.7	97	10	100	10	100
triphenylmethane (0.25)	triphenylmethanol	9.8	98	10	100	9.3	93	8.65	86.5
cumene (0.25)	2-phenyl-2-propanol					1.66	30.7	3.56	57.7
	acetophenone					1.41		2.21	
ethylbenzene (0.25)	1-phenylethanol	0.47	19.3	0.57	24	0.75	29.1	0.71	32.1
	1-acetophenone	1.46		1.826	2.16	2.5			

a Turnover number (TON) = mol of product/mol of catalyst.

b Efficiency (%) = moles of total products/mol of oxidant ×100.

**Table 10:** Catalytic Activities of Complexes with H_2_O_2_ (Catalyst:H_2_O_2_ = 1:100)

substrate (mmol)	product	complex **1a**		complex **2a**		complex **3a**		complex **4a**	
		TON^a^	efficiency^b^	TON	efficiency	TON	efficiency	TON	efficiency
thioanisole (0.25)	thioanisole oxide	1.8	1.8	2.5	2.5	27.8	27.8	27	27
9,10-DHA (0.05)	anthracene	15.9	15.9	23.2	23.2	28.2	28.2	26.7	26.7
1,4-CHD	benzene	16.5	16.5	22.4	22.4	54.4	54.4	49.5	49.5
triphenylmethane (0.25)	triphenylmethanol	22.8		30.8	30.8	8	8	10	10
cumene (0.25)	2-phenyl-2-propanol					6.9	7.49	12.6	14.2
	acetophenone					0.59		1.6	
ethylbenzene (0.25)	1-phenylethanol	5.3	12.33	7	16.02	5.42	13.24	4.4	12.8
	1-acetophenone	7.03		9.02		7.82		8.4	
toluene (0.25)	benzaldehyde	4	4	6.1	6.1	4.5	4.5	3.5	3.5
cyclohexane (0.5)	cyclohexanol	4.38	8.98	6.2	13.4	5.86	12.33	5.6	11.87
	cyclohexanone	4.6		7.2		6.47		6.27	

a Turnover number (TON) = mol of product/mol of catalyst.

b Efficiency (%) = moles of total products/mol of oxidant ×100.

When H_2_O_2_ was used as an oxidant, **3a** and **4a** gave much better yields than did **1a** and **2a**. The identities of the active oxidants were not determined in these reactions. Studies on the reactions of the parent complex [Fe^II^(L)(N4Py)]^n+^ (L = monodentate ligand) with H_2_O_2_ show that an Fe^III^–OOH complex is formed initially,^42^ and this hydroperoxo complex may undergo O–O bond homolysis to generate the corresponding Fe^IV^=O complex and a hydroxyl radical. However, at high concentrations of hydrogen peroxide, studies indicate that the formation of an Fe^III^(OH) species is favored over ferryl formation.^43^ A relatively low steady-state concentration of hydrogen peroxide should therefore favor the formation of oxidative species (Fe^IV^=O, OH·). Considering the high concentrations of hydrogen peroxide used in our study and the low catalytic efficiencies (Table 9), substantial formation of an unproductive Fe^III^(OH) species cannot be excluded nor can the oxidation of substrates by hydroxyl radicals formed in Fenton reactions be excluded.

For triphenylmethane and 9,10-dihydroanthracene (9,10-DHA) — the substrates in this study that contain the greatest steric bulk around the C–H bond — the catalytic oxidations in this work give excellent yields. The small difference in yields between 9,10-DHA and 1,4-cyclohexadiene (1,4- CHD), which involve similar BDEs but very different steric environments, also indicates that the electronic contribution of the ligand donor moieties mainly dictates the reactivities of the complexes rather than the steric influence of the ligands.

## Summary and Conclusions

We report the synthesis and characterization of four new Fe^II^ complexes and four corresponding Fe^IV^=O complexes supported by four new pentadentate nonheme ligands based on the N4Py framework. As discussed above, the spin states and the redox potentials for the Fe^II^ complexes, the absorption wavelengths in the near-IR region, the half-lives, and the HAT/OAT reactivities of the Fe^IV^=O complexes are all consistent with ligand **L**^**2**^, containing two (*N*-methyl)imidazolyl donors, exerting the weakest ligand field in these complexes. This is followed by **L**^**1**^, where only one pyridyl group is replaced with an (*N*-methyl)imidazolyl group. The sequences of λ_max_ for the characteristic near-IR absorptions of the Fe^IV^=O complexes and of the half-lives at room temperature are consistent with the replacement of pyridyl arms with (*N*-methyl)imidazolyl moieties rendering the ligand fields of **L**^**1**^ and **L**^**2**^ to be weaker than for the parent N4Py ligand. Compared to the parent complex [Fe^IV^(O)(N4Py)]^2+^, complexes [Fe^IV^(O)(**L**^**1**^)]^2+^ (**1b**) and [Fe^IV^(O)(**L**^**2**^)]^2+^ (2**b**) exhibit enhanced reactivity in both the OAT and HAT reactions. The ferryl complexes **1b**–**4b** are robust for the conversion of the OAT to thioanisole with excellent turnover numbers. Using H_2_O_2_ as the oxidant, complexes **1b**–**4b** also displayed good performance in catalytic oxidation reactions (HAT and OAT). The crystal structures of Fe^II^ complexes **1a**–**4a** and Fe^IV^=O complex **4b** and the reactivities of the ferryl complexes toward bulky substrates show that the steric influence of the four new ligands on the sixth (axial) position in the iron complexes is relatively minor.

Table 3 (*vide supra*) provides a clear indication of the effect the different N-donor substituents in the ligand framework have on the redox potential of their corresponding iron complexes and thus indicates the effective overall donor capacity of the pentadentate ligands. The half-wave potentials (*E*_1/2_) of the Fe^III^/Fe^II^ redox couple become more positive in the following sequence of the ligands: **L**^**2**^ (two (*N*-methyl)imidazolyl) < **L**^**1**^ (one (*N*-methyl)imidazolyl) < **L**^**4**^ (two isoquinolines) < **L**^**3**^ (one isoquinoline) < N4Py (*cf*. Figure 9 and Table 3). The trend in half-wave potentials can be interpreted as a decrease in the electron density over the iron center due to the electron-withdrawing effect of the groups. It is in agreement with the effect seen by introducing electron-withdrawing and electron-donating substituents in para-position of the central (axial) pyridyl moiety in Fe^II^ complexes of the pentadentate ligand Py5Me_2,_^44^ where the more electron-withdrawing groups give a more positive *E*_1/2_ for the Fe^III^/Fe^II^ couple. In this study, the (*N*-methyl)imidazolyl substituent is essentially a pure sigma-donor ligand and leads to the largest electron density on the ferrous ion and the weakest ligand field/highest-lying HOMOs. The isoquinoline and pyridyl substituents are σ-donors and π-acceptors; the metal π to ligand π* back-donation for these nitrogen donors leads to lower electron densities on the ferrous ions and stronger ligand fields/lower-lying HOMOs.

In summary, the observed HAT/OAT reactivities of the ferryl complexes in the present study may be directly correlated to the electronic properties of the ligand substituents rather than to steric effects leading to consequential electronic effects.

## Experimental Section

### Materials

All reagents and solvents were obtained from commercial sources and used without further purification unless otherwise noted. All solvents were of at least 99.5% purity. 1-Methyl-1-H-2-carbaldehyde,^45^ bis(2-pyridyl)methylamine,^46^ and *N*-[di(2-pyridinyl)methy]-*N*-(2-pyridinylmethyl)methylamine^47^ were prepared according to literature procedures.

### Physical Methods

Preparation and handling of air-sensitive reactions were carried out under a nitrogen atmosphere in a glovebox. All room-temperature NMR spectra were collected on a Bruker Avance 400 MHz spectrometer in CD_3_CN or DMSO-*d*_6_ solvents. Variable-temperature NMR spectra were collected on Bruker Avance or Varian Inova 500 MHz spectrometers in CD_3_CN solvents. Fourier transform infrared spectroscopy on KBr pellets was performed on a Cary 630 FTIR Spectrometer (IR spectra of Fe^II^ and Fe^IV^=O complexes are found in the Supporting Information). The electrochemical analyses were recorded using a Pine Research WaveNow potentiostat, a 2 mm glassy carbon working electrode wiped between scans, a platinum wire counter electrode, and Ag/Ag^+^ (silver wire in 0.005 M AgNO_3_, 0.10 M Bu_4_NPF_6_, and CH_3_CN) isolated by a frit used as a reference electrode and calibrated versus ferrocene. Anhydrous acetontrile (Carlo Erba) was used as the solvent with 0.10 M tetrabutylammonium hexafluorophosphate as the supporting electrolyte. All potentials are referenced to the ferrocene/ferrocenium couple (0.400 V *vs* NHE). The solutions for the electrochemical experiments were purged with solvent-saturated nitrogen and kept under an inert atmosphere throughout the measurements. HRMS was performed using a UHPLC/SFC-QTOF instrument. UV/vis spectra and all kinetic experiments were performed on a CARY100 UV/vis spectrophotometer (Agilent Technologies) equipped with a R928 PMT detector and a USP-203-B Unisoku cryostat, which permits monitoring of the temperature of the experiments from 193 to 373 K. All UV/vis spectra were measured using a four-side transparent quartz cuvette with a screw cap (path length: 10 mm). Product analyses were carried out by GC measurements on an Agilent Technologies 7820 A gas chromatograph with a 16-sample automatic liquid sampler and a flame ionization detector. The products passed through an SPB-35 capillary GC column and were identified by their different retention times. Zero-field and magnetic field Mössbauer measurements were carried out with ^57^Co sources in Rh matrices on alternating constant *Wissel* Mössbauer spectrometers equipped with *Janis* or *OptiCool* helium cryostats. Magnetic susceptibility measurements were made with a *Quantum-Design* MPMS3 SQUID magnetometer equipped with a 7 T magnet. A detailed description of the experimental setup and data treatment for Mössbauer and magnetic measurements is found in the Supporting Information.

### Synthesis and Characterization of Ligands

#### Synthesis of *N*-(1-Methyl-2-imidazolyl)methyl-*N*-(2-pyridyl)-methyl-*N*-(bis-2-pyridylmethyl)-amine (**L**^**1**^)

A round-bottom flask was charged with *N*-[di(2-pyridinyl)methyl]-*N*-(2-pyridinylmethyl)methylamine (N3Py, *cf.*Scheme 1, 1.00 g, 3.6 mmol) and NaBH(CH_3_COO)_3_ (5.34 g, 25.2 mmol) in CH_2_Cl_2_. The solution was kept in an ice bath for 0.5 h under a nitrogen atmosphere. After that, 1-methyl-1*H*-imidazole-2-carboxaldehyde (B, 0.4 g, 3.6 mmol) was added. The ice bath was removed after 1 h, after which the solution was left stirring for 48 h at room temperature and under a nitrogen atmosphere. Subsequently, the solution was neutralized with saturated NaHCO_3_ solution until no bubbles were generated. The resultant solution was then extracted with CH_2_Cl_2_, washed with brine, and dried over Na_2_SO_4_. Evaporation of the organic solvent afforded the crude ligand **L**^**1**^ as a brown oil (*cf*. Scheme 1). The crude product was purified by passing it through a silica column using a mixture of ethyl acetate, dichloromethane, and triethylamine (2:1:1) as eluent. Yield 1.05 g (75%). ^1^H NMR (400 MHz, DMSO) δ 8.49 (dddd, *J* = 10.6, 4.8, 1.9, 0.9 Hz, 3H), 7.87–7.66 (m, 5H), 7.45 (d, *J* = 7.8 Hz, 1H), 7.32–7.18 (m, 3H), 6.96 (d, *J* = 1.2 Hz, 1H), 6.74 (d, *J* = 1.2 Hz, 1H), 5.25 (s, 1H), 3.79 (d, *J* = 17.9 Hz, 4H), 3.40 (s, 3H) (Figure S1).

#### Synthesis of *N*-bis(1-Methyl-2-imidazolyl)methyl-*N*-(bis-2-pyridylmethyl)amine (**L**^**2**^)

Ligand **L**^**2**^ was obtained by the reaction of bis(2-pyridyl)methylamine (A) with two equivalents of 1-methyl-1*H*-imidazole-2-carboxaldehyde (B) in the presence of NaBH(CH_3_COO)_3_ in CH_2_Cl_2_ (*cf*. Scheme 1). The subsequent workup was identical with that used for Ligand **L**^**1**^. Yield: 55%. ^1^H NMR (400 MHz, DMSO) δ 8.60–8.44 (m, 2H), 7.83–7.68 (m, 2H), 7.64–7.42 (m, 2H), 7.25 (dddd, *J* = 14.9, 7.5, 4.8, 1.2 Hz, 2H), 7.02 (dd, *J* = 6.9, 1.2 Hz, 2H), 6.77 (dd, *J* = 17.8, 1.2 Hz, 2H), 5.28–4.95 (m, 1H), 3.80 (s, 2H), 3.77–3.56 (m, 2H), 3.30 (s, 6H). ^13^C NMR (101 MHz, DMSO) δ 172.53, 161.86, 159.87, 149.23, 145.15, 137.07, 136.75, 126.89, 124.71, 122.66, 122.59, 122.28, 122.02, 70.02, 68.25, 55.37, 46.51, 40.46, 32.36, 21.60, 11.68 (Figures S2–S4).

#### Synthesis of *N*-(Isoquinolin-3-ylmethyl)-1,1-di(pyridin-2-yl)-*N*-(pyridin-2-ylmethyl)methanamine (**L**^**3**^)

A round-bottom flask was charged with a mixture of *N*-[di(2-pyridinyl)methyl]-*N*-(2-pyridinylmethyl)methylamine (N3Py, 1.00 g, 3.6 mmol) and potassium carbonate (2.3 g, 16 mmol) in CH_3_CN. The solution was stirred for several minutes at room temperature. After that, 3-(chloromethyl)isoquinoline (C, *cf.*Scheme 1, 0.7 g, 4 mmol) was added, and the mixture was then refluxed for 48 h. After cooling to room temperature, the solvent was evaporated, and the residue was purified by flash column chromatography (Al_2_O_3_ neutral activated, ethyl acetate/hexane/triethylamine (10:5:1, R_f_ = 0.3) to give *N*-(isoquinolin-3-ylmethyl)-1,1-di(pyridin-2-yl)-*N*-(pyridin-2-ylmethyl)methanamine (**L**^**3**^, 0.76 g, 51%) as a dark red oil. ^1^H NMR (400 MHz, CDCl_3_) δ 9.25 (s, 1H), 8.61 (ddd, *J* = 4.8, 1.9, 0.9 Hz, 2H), 8.55–8.46 (m, 1H), 7.96 (d, *J* = 8.2 Hz, 1H), 7.92 (s, 1H), 7.84–7.77 (m, 3H), 7.74–7.52 (m, 6H), 7.17 (ddd, *J* = 7.5, 4.8, 1.2 Hz, 2H), 7.09 (ddd, *J* = 7.5, 4.9, 1.2 Hz, 1H), 5.49 (s, 1H), 4.18 (s, 2H), 4.09 (s, 2H) (Figure S5).

#### Synthesis of *N*,*N*-bis(Isoquinolin-3-ylmethyl)-1,1-di(pyridin-2-yl)methanamine (**L**^**4**^)

A mixture of di(2-pyridyl)methylamine, (A, 0.67 g, 3.6 mmol), potassium carbonate (2.3 g, 16 mmol), and acetonitrile were stirred for several minutes at room temperature. To the mixture was added 3-(chloromethyl)isoquinoline (C, 1.4 g, 8 mmol). The subsequent workup was identical to that used for Ligand **L**^**3**^ to give **L**^**4**^ (1.3 g, 77%) as a dark red oil. ^1^H NMR (400 MHz, CDCl_3_) δ 9.17 (s, 2H), 8.58 (d, *J* = 4.8 Hz, 2H), 7.97 (s, 2H), 7.88 (d, *J* = 8.2 Hz, 2H), 7.84 (d, *J* = 7.9 Hz, 2H), 7.77 (d, *J* = 8.2 Hz, 2H), 7.68 (d, *J* = 7.6 Hz, 2H), 7.62 (d, *J* = 7.2 Hz, 2H), 7.51 (t, *J* = 7.5 Hz, 2H), 7.14 (td, 2H), 5.55 (s, 1H), 4.21 (s, 4H). ^13^C NMR (101 MHz, CDCl_3_) δ 160.4, 151.9, 149.3, 136.3, 130.1, 127.6, 127.4, 126.6, 126.5, 123.9, 121.9, 118.8, 72.2, 57.10 (Figures S6 and S7).

### Synthesis and Characterization of Complexes

Complexes [Fe^II^(CH_3_CN)(**L**^**1**^)](ClO_4_)_2_ (**1a**·(ClO_4_)_2_), [Fe^II^(CH_3_CN)(**L**^**2**^)](ClO_4_)_2_ (**2a**·(ClO_4_)_2_), [Fe^II^(**L**^**3**^)(CH_3_CN)](ClO_4_)_2_ (**3a**·(ClO_4_)_2_), and [Fe^II^(CH_3_CN)(**L**^**4**^)](ClO_4_)_2_ (**4a**·(ClO_4_)_2_) were prepared by the reaction of one equivalent of **L**^**1**^/**L**^**2**^/**L**^**3**^/**L**^**4**^ with one equivalent of Fe^II^ salts in a minimum amount of dry acetonitrile at room temperature in a N_2_ atmosphere (Scheme 2, Supporting Information). ***CAUTION!****Perchlorate salts are potentially explosive and should be handled with care*.

Complexes [Fe^II^(CH_3_CN)(**L**^**1**^)](OTf)_2_ (**1a**·(OTf)_2_), [Fe^II^(CH_3_CN)(**L**^**2**^)](OTf)_2_ (**2a**·(OTf)_2_), [Fe^II^(**L**^**3**^)(CH_3_CN)](OTf)_2_ (**3a**·(OTf)_2_), and [Fe^II^(CH_3_CN)(**L**^**4**^)](OTf)_2_ (**4a**·(OTf)_2_) were prepared by the reaction of ligand (**L**^**1**^/**L**^**2**^/**L**^**3**^/**L**^**4**^) with one equivalent of Fe(OTf)_2_·2MeCN. Similarly, the reaction of **L**^**4**^ with Fe(BF_4_)_2_·6H_2_O in a glovebox gave [Fe^II^(CH_3_CN)(**L**^**4**^)](BF_4_)_2_ (**4a**·(BF_4_)_2_). The methods of crystallizations of **1a**–**4a** are described in the Supporting Information.

#### **1a**·(ClO_4_)_2_

^1^H NMR (400 MHz, CD_3_CN) δ 9.19–8.92 (m, 3H), 7.93 (d, *J* = 6.0 Hz, 4H), 7.70 (td, *J* = 7.6, 1.4 Hz, 1H), 7.41 (d, *J* = 13.9 Hz, 4H), 7.18 (d, *J* = 7.3 Hz, 1H), 7.09 (s, 1H), 6.49 (s, 1H), 4.55–4.12 (m, 4H), 3.41 (s, 3H) (Figure S8). HRMS: *m*/*z* = 213.0631 [Fe^II^(**L**^**1**^)]^2+^(z = 2) calc. 213.0628, *m*/*z* = 525.0754 [Fe^II^(**L**^**1**^)(ClO_4_)]^+^(z = 1) calc. 525.0741 (Figure S32).

#### **2a**·(ClO_4_)_2_

^1^H NMR (400 MHz, CD_3_CN) δ 79.29, 52.91, 37.38, 25.22, 16.73, 14.00 (s, 1H), 13.00, 11.89, 11.79, 10.19, 6.48, 5.43, 4.58, 3.3, 1.12, −1.60 (Figure S10). HRMS: *m*/*z* = 214.5684 [Fe^II^(**L**^**2**^)]^2+^(*z* = 2) calc. 214.5628, *m*/*z* = 528.0861 [Fe^II^(**L**^**2**^)(ClO_4_)]^+^(*z* = 1) calc. 528.0850 (Figure S33).

#### **3a**·(ClO_4_)_2_

^1^H NMR (400 MHz, CD_3_CN) δ 9.81 (s, 1H), 9.10–9.04 (m, 1H), 8.98 (ddt, *J* = 10.9, 5.5, 1.2 Hz, 2H), 8.23–8.16 (m, 1H), 8.04–7.90 (m, 4H), 7.86–7.72 (m, 3H), 7.64 (td, *J* = 7.7, 1.5 Hz, 1H), 7.49 (s, 1H), 7.47–7.30 (m, 3H), 7.05 (d, *J* = 7.9 Hz, 1H), 6.38 (s, 1H), 4.50–4.26 (m, 4H) (Figure S12). HRMS ([^57^Fe^II^(**L**^**3**^)(CH_3_CN)](OTf)_2_): *m*/*z* = 237.0951 [^57^Fe^II^(**L**^**3**^)]^2+^ (z = 2) calc. 237.0651, and *m*/*z* = 623.0323 [^57^Fe^II^(**L**^**3**^)(OTf)]^+^ (z = 1) calc. 623.0828 (Figure S34, mass spectrum obtained on an ^57^Fe sample).

#### **4a**·(ClO_4_)_2_

^1^H NMR (400 MHz, CD_3_CN) δ 9.83 (s, 2H), 9.03 (d, *J* = 7.8 Hz, 2H), 8.15 (d, *J* = 8.5 Hz, 2H), 8.06–7.89 (m, 4H), 7.81–7.64 (m, 6H), 7.44 (s, 2H), 7.38 (ddd, *J* = 7.3, 5.5, 2.0 Hz, 2H), 6.40 (s, 1H), 4.49 (d, *J* = 17.1 Hz, 2H), 4.39–4.25 (m, 2H) (Figure S14). HRMS: *m*/*z* = 261.5743 [Fe^II^(**L**^**4**^)]^2+^ (*z* = 2) calc. 261.5730, and *m*/*z* = 622.0962 [Fe^II^(**L**^**4**^)(ClO_4_)]^+^ (z = 1) calc. 622.0944 (Figure S35).

#### **4a**·(BF_4_)_2_

Elemental analysis (C_33_H_28_B_2_F_8_FeN_6_): calc. C, 53.70; H, 3.82; N, 11.39, found C, 53.83; H, 3.85; N, 11.41. *m*/*z* = 568.1446 [Fe^II^(**L**^**4**^) (HCOO)]^+^ (*z* = 1) (calc. 568.14).

### Crystal Structure Determinations

The crystals of **2a**·(ClO_4_)_2_ and **5**·(OTf)_2_ were immersed in cryo-oil, mounted in a Nylon loop, and measured at a temperature of 120 K. The X-ray diffraction data were collected on Bruker Kappa Apex II and Bruker Kappa Apex II Duo diffractometers using Mo Kα radiation (λ = 0.710 73 Å). The *APEX*2^48^ program package was used for cell refinements and data reductions. The structure was solved by the charge flipping technique (SUPERFLIP)^49^ or direct methods using the *SIR*2011 program^50^ with the *Olex*2 graphical user interface.^51^ A semiempirical numerical absorption correction based on equivalent reflections (*SADABS*)^52^ was applied to all data. Structural refinements were carried out using *SHELXL-*97.^53^ The crystal of **2**·(ClO_4_)_2_ was diffracting only weakly, and therefore atoms N2, C16, C17, C18, C19, and C20 were restrained to have the same *U*_ij_ components within the standard uncertainty of 0.02. In **2**·(ClO_4_)_2_, one molecule of the acetonitrile of crystallization was disordered over two sites with equal occupancy. Hydrogen atoms were positioned geometrically and were also constrained to ride on their parent atoms, with C–H = 0.95–0.1.00 Å and *U*_iso_ = 1.2–1.5 *U*_eq_ (parent atom).

X-ray data for **1a**·(OTf)_2_ and **3a**·(OTf)_2_ were collected on a BRUKER D8-QUEST diffractometer (monochromated Mo–Kα radiation, λ = 0.71073 Å) by use of ω or ω and φ scans at low temperature. The structures were solved with SHELXT^54^ and refined on F^2^ using all reflections with SHELXL.^55^ Non-hydrogen atoms were refined anisotropically. Hydrogen atoms were placed in calculated positions and assigned to an isotropic displacement parameter of 1.5/1.2 *U*_eq_(C). One CF_3_SO_3_^–^ ion was found to be disordered about two positions in **1a**·(OTf)_2_ (occupancy factors: 0.809(3)/0.191(3)) and about three positions in **3a**·(OTf)_2_ (occupancy factors: 0.391(2)/0.305(2)/0.303(3)). SAME and RIGU restraints and EADP constraints were applied to model the disordered parts. In **1a**,·(OTf)_2_ parts of the ligand were found to be disordered (occupancy factors: 0.506(7)/0.494(7)). SAME, SADI (*d*(Fe–N)), and SIMU restraints were used to model the disorder. The noncoordinating MeCN in **3a**·(OTf)_2_ was refined at half occupancy. Absorption corrections were performed by the multiscan method with SADABS.^52^

X-ray diffraction data for **4a**·(BF_4_)_2_ were collected at room temperature with an Oxford Diffraction Excalibur 3 system using ω-scans and Mo–Kα radiation (λ = 0.71073 Å). The data were extracted and integrated using Crysalis RED. The structure was solved by direct methods and refined by full-matrix least-squares calculations on F^2^ using SHELXT,^55^ SHELXL,^54^ integrated in OLEX2.^49^ Disordered electron density corresponding to 0.33 MeCN/Fe was handled using the solvent mask in OLEX2.

Because the crystals of **4b**·(ClO_4_)_2_ were too small to be measured at a regular X-ray source, data were collected at the MAX IV synchrotron. The crystals were measured at the BioMAX beamline,^56^ equipped with a Dectrix Eiger X 16 M detector and an Arinax MD3 microdiffractometer. The beamline wavelength was set to 0.6199 for the data collection, and the beam size was set to 0.05 mm. A 900° data set was collected about a single axis at 22 ms per degree and a beam attenuation of 85% to avoid exceeding the count rate of the detector. The software program XDS^56^ was used for data processing. Data were integrated to a maximum resolution (sin θ/λ) of 0.655 (θ_max_ = 28.832 deg), with a data completeness of 84.9%. The space group was determined with the CCP4 program Pointless^57^ and the structure determined *ab initio* with SHELXD.^58^

The crystallographic details are summarized in Tables S2–S8, Supporting Information.

### Generation of Fe^IV^=O Complexes

To an acetonitrile solution (0.5 mM) of the perchlorate salts of **1a**, **2a**, and **4a**, and the triflate salt of **3a**, three equiv of IBX ester was added at 25 °C. Immediately, the reddish-brown solutions turned pale green. Based on HRMS of the pale green solution originating from **1a**·(ClO_4_)_2_, the peaks at *m*/*z* = 221.1334 and *m*/*z* = 541.2484 correspond to the formulations of [Fe^IV^=O(**L**^**1**^)]^2+^ (calc. 221.0602) and [Fe^IV^=O(**L**^**1**^)(ClO_4_)]^+^ (calc. 541.0690), respectively (Figure S36). Based on HRMS of the pale green solution originating from **2a**·(ClO_4_)_2_, the peaks at *m*/*z* = 222.5657 and *m*/*z* = 544.0801 correspond to the formulations of [Fe^IV^=O(**L**^**2**^)]^2+^ (calc. 222.5657) and [Fe^IV^=O(**L**^**2**^)(ClO_4_)]^+^ (calc. 544.0799.), respectively (Figure S37). Based on HRMS of the pale green solution originating from an enriched sample of [^57^Fe^II^(**L**^**3**^)(CH_3_CN)](OTf)_2_, the peaks at *m*/*z* = 245.0928 ([^57^Fe^IV^=O(**L**^**3**^)]^2+^ (calc. 245.0628) and *m*/*z* = 639.0225 ([^57^Fe^IV^=O(**L**^**3**^)(OTf)]^+^ (calc. 639.0776)) correspond to complex **3b** (Figure S38). Based on HRMS of the pale green solution originating from **4a·**(ClO_4_)_2_, the peaks at *m*/*z* = 269.5718 and *m*/*z* = 638.0911 correspond to the formulations of [Fe^IV^=O(**L**^**4**^)]^2+^ (calc. 269.5700) and [Fe^IV^=O(**L**^**4**^)(ClO_4_)]^+^ (calc. 638.0900), respectively (Figure S39). After deaeration of the solutions of the Fe^IV^=O complexes, UV/vis spectroscopy was performed at 25 °C with maximum absorption at 706 nm (ε = 379 M^–1^ cm^–1^) for [Fe^IV^=O(**L**^**1**^)]^2+^(complex **1b**), 721 nm (ε = 274 M^–1^ cm^–1^) for [Fe^IV^=O(**L**^**2**^)]^2+^ (complex **2b**)), 695 nm (ε = 540 M^–1^ cm^–1^) for [Fe^IV^=O(**L**^**3**^)]^2+^ (complex **3b**)), and 696 nm (ε = 544 M^–1^ cm^–1^) for [Fe^IV^=O(**L**^**4**^)]^2+^ (complex **4b**) separately. The time course decays of the Fe^IV^=O complexes were then monitored, giving the half-lives for complex **1b** (1.67 h), complex **2b** (16 h), complex **3b** (64 h), and complex **4b** (45 h).

### HAT Reactions

Kinetic experiments were performed by adding an appropriate amount of the relevant substrate (2.5–500 mM) to a 0.5 mM solution of complex **1***b***/2b/3***b***/4b** in CH_3_CN (generated by addition of three equivalents of IBX ester to **1a**·(ClO_4_)_2_/**2a**·(ClO_4_)_2_/**3a**·(ClO_4_)_2_/**4a**·(ClO_4_)_2_). After deaeration with argon, the time course decay of the Fe^IV^=O complex was monitored at 25 °C by UV/vis spectroscopy. Rate constants, *k*_obs_, were evaluated by pseudo-first-order fitting of the absorbance decay at 706 nm/721 nm/695 nm/696 nm for complex **1b/2b/3b/4b**. Second-order rate constants were evaluated from the slope of the linear fits of *k*_obs_ versus concentration-dependence data. To isolate the organic products, the solutions after the end of the reaction were passed through a silica column, using ethyl acetate as the eluent, to remove the metal complex. The ethyl acetate solutions were then analyzed by GC using a known concentration of naphthalene solution as the quantification standard. Results obtained from these studies are listed in Table 6.

### OAT (Thioanisole Oxidation) Reactions

The Fe^IV^=O complexes **1***b***/2b/3***b***/4b** solutions were prepared as described above. The solutions were placed in a cuvette, and the temperature of the UV/vis instrument was adjusted to −30 °C. Subsequently, appropriate amounts of the substrate, thioanisole, were added to the Fe^IV^=O solution. After that, the subsequent decay was monitored. Time courses were subjected to pseudo-first-order fitting, and second-order rate constants were evaluated from the concentration-dependence data. The products were quantified following the procedure already described. Results obtained from these studies are listed in Table 7.

### Analysis of the Products after Oxidation of Substrates

The Fe^II^ precursor complexes (**1a**–**4a**) (0.005 mmol) were dissolved in 3 mL of dry, deoxygenated acetonitrile. To the resulting solution, 10 equiv of IBX ester or 100 equiv of H_2_O_2_ was added along with the substrates at 298 K under an inert atmosphere. The reaction occurred under stirring for 4 h. Then, the resulting solution was passed through a silica column (60–120 mesh size) using ethyl acetate as an eluent. The combined organic phase was then analyzed by GC. For GC analyses, naphthalene was used as an internal standard, and the products were identified by comparison of their GC retention times with those of authentic compounds.

#### Theoretical Methods

Geometry optimization of complex **4a** was performed in vacuum at the DFT level using the PBE0^59,60^ and def2-TZVP(-f) basis set for all atoms.^61−63^ Dispersion effects (if existent) were included using Grimme’s D3 correction with Becke-Johnson (BJ) damping.^64,65^ The evaluation of the four-center integrals was accelerated with the RIJCOSX algorithm, using the resolution of identity approximation for the Coulomb part (RIJ) and the chain of spheres approach for the Fock exchange (COSX).^66,67^ RIJ requires the specification of an auxiliary basis set for the Coulomb part (Def2/J) and a numerical integration grid for the exchange part (DefGrid-2).^68^ Analytical harmonic vibrational frequency calculations were conducted to verify whether the ground state is the minima on the potential energy surface. TD-DFT^69^ was employed to obtain the first 25 singlet excited states, including the solvent correction, using the linear response conductor-like polarizable continuum model (LR-CPCM) method.^70^ All these calculations were performed using Orca 5.0.3,^71^ and the geometric representations of the complexes were obtained using the Chemcraft program.^72^

## Supporting Information Available

Synthesis methods; crystallographic data; UV/vis spectra; FTIR spectra; NMR spectra; Mössbauer spectra; SQUID measurements; cyclic voltammograms; tables of selected bond distances (Å) and bond angles (deg) of complexes; optimized ground-state geometries; and CCDC entries (1a.(OTf)_2_), (2a.(ClO_4_)_2_), (3a.(OTf)_2_), (4a.(BF_4_)_2_), (4b.(ClO_4_)_2_), and (5.(OTf)_2_) with the supplementary crystallographic data for the structures described in this paper (PDF)
