# Supplementary material for: Nonheme FeIV=O Complexes Supported by Four Pentadentate Ligands: Reactivity toward H- and O- Atom Transfer Processes
Source: Inorg Chem. 2023 Nov 1;62(45):18338–56. doi: 10.1021/acs.inorgchem.3c02526 (PMC10647104; doi:10.1021/acs.inorgchem.3c02526)
Supplement: Supplementary file 1 — ic3c02526_si_001.pdf [file ic3c02526_si_001.pdf]

Supplementary Information

Non-heme Fe^{IV}=O complexes supported by four pentadentate ligands: reactivity towards H- and O-atom transfer processes

Yong Li,[a] Reena Singh,[a] Arup Sinha,[a] George C. Lisensky,[b] Matti Haukka,[c] Justin Nilsson,[a] Solomon Yiga,[d] Serhiy Demeshko,[e] Sophie Jana Gross,[e] Sebastian Dechert,[e] Ana Gonzalez,[f] Giliandro Farias,[g] Ola F. Wendt,[d,*] Franc Meyer,[e] Ebbe Nordlander[a,*]

a. Chemical Physics, Department of Chemistry, Lund University, Box 124, SE-221 00 Lund, Sweden, E-mail: Ebbe.Nordlander@chemphys.lu.se
b. Department of Chemistry, Beloit College, 700 College Street, Beloit, Wisconsin 53511, U.S.A.
c. Department of Chemistry, University of Jyväskylä, P.O. Box-35, Jyväskylä, FI-40014, Finland
d. Center for Analysis and Synthesis, Department of Chemistry, Lund University, P.O. Box 124, SE-22100 Lund, Sweden, E-mail: Ola.Wendt@chem.lu.se
e. Georg-August Universität Göttingen, Institut für Anorganische Chemie, Tammanstrasse 4, D-37077 Göttingen, Germany
f. MAXIV Laboratory, Lund University, P.O. Box 118, SE-221 00 Lund, Sweden
g. Department of Chemistry, Federal University of Santa Catarina, 88040900, Florianópolis, SC, Brazil

NMR spectra of ligands

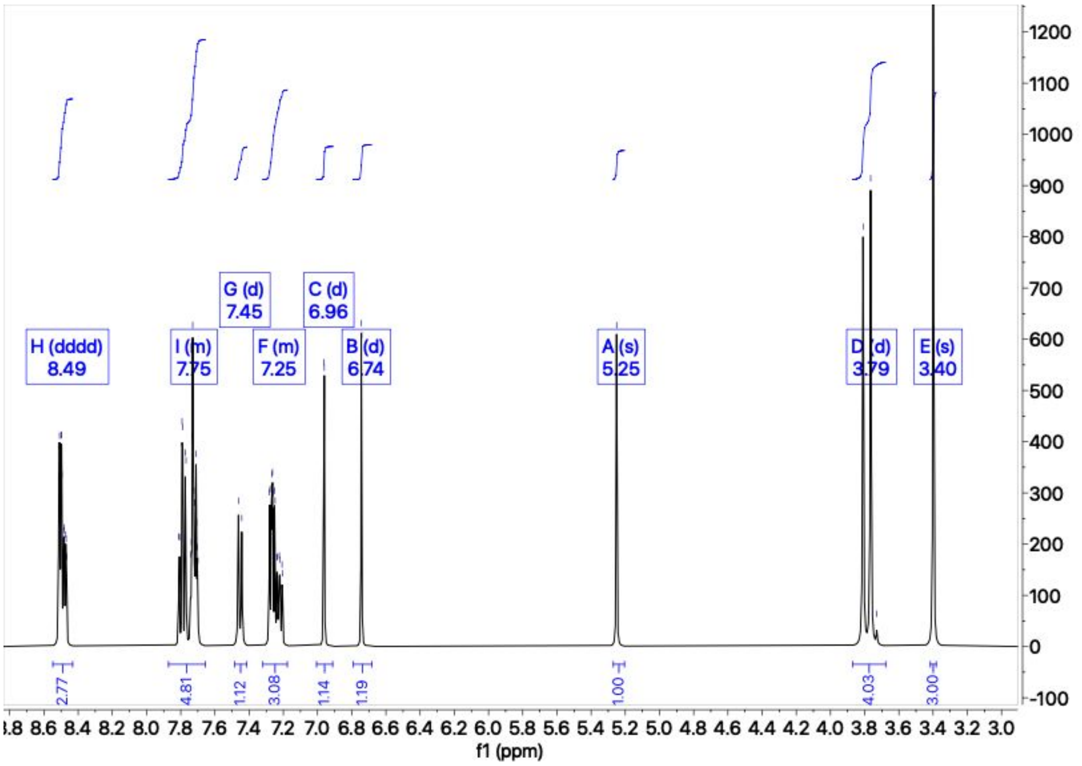

Figure S1. The ^{1}H NMR spectrum of ligand $\mathbf{L^1}$ in d_6-DMSO (400 MHz) measured at 298 K.

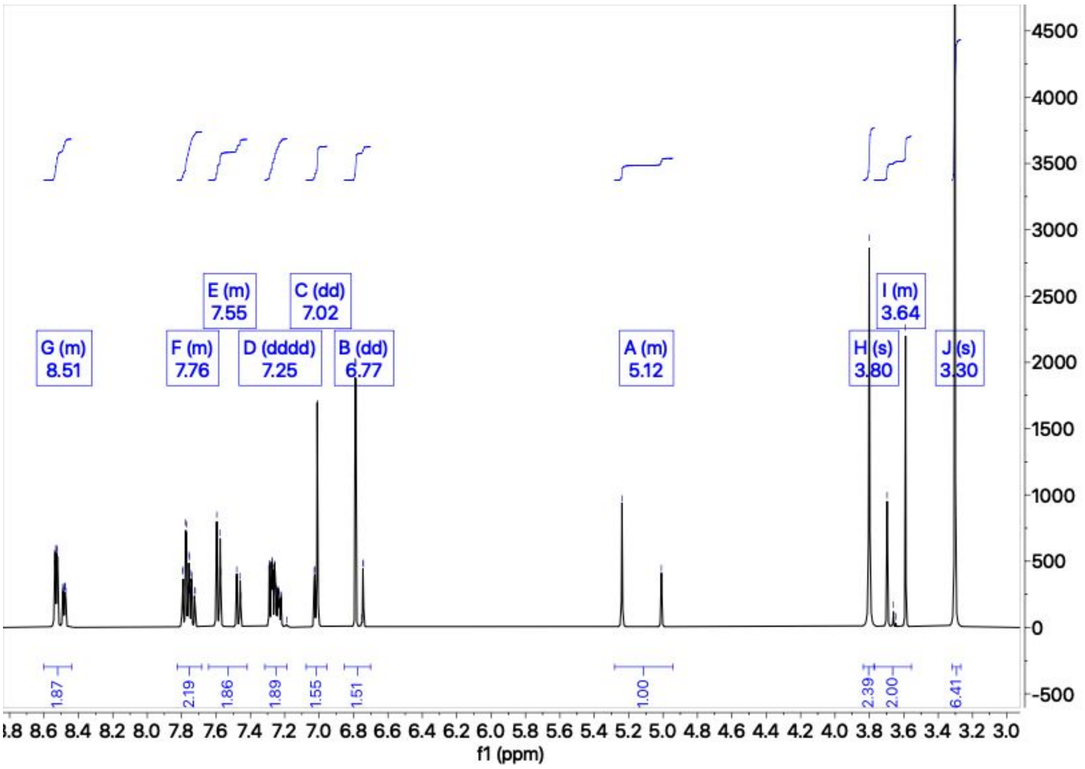

Figure S2. The ^{1}H NMR spectrum of ligand $\mathbf{L^2}$ in d_6-DMSO (400 MHz) measured at 298 K.

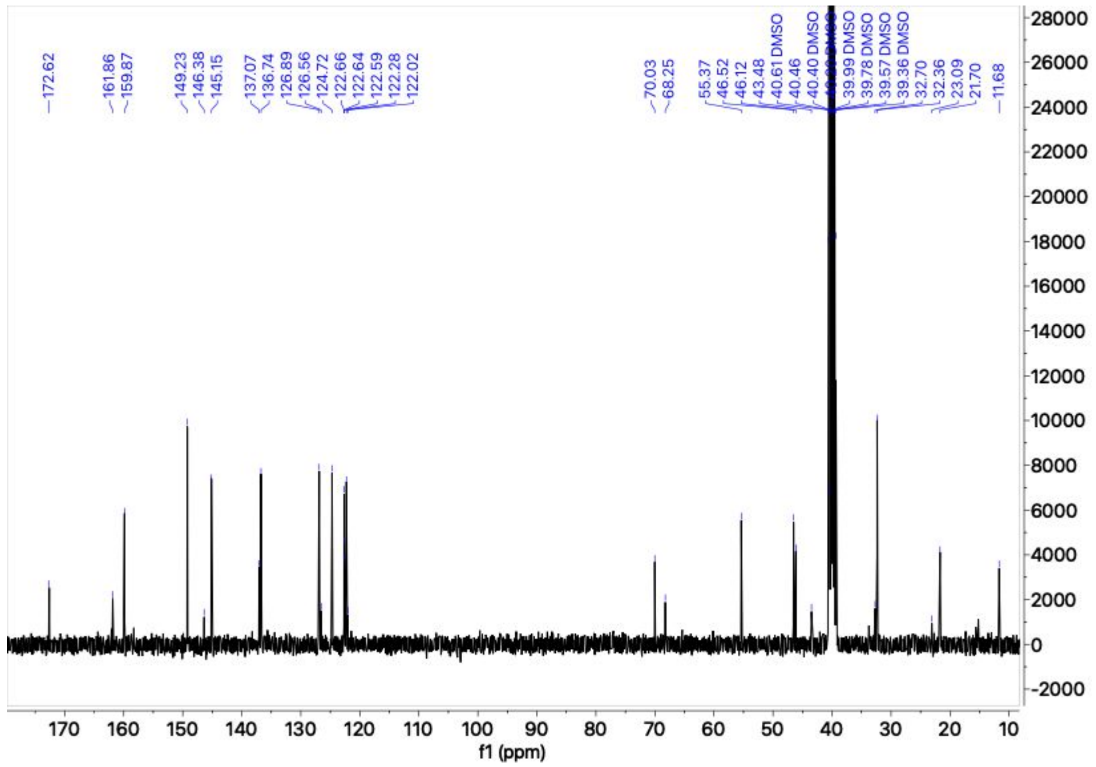

Figure S3. The ^{13}C NMR spectrum of ligand $\mathbf{L^2}$ in d_6-DMSO (100 MHz) measured at 298 K.

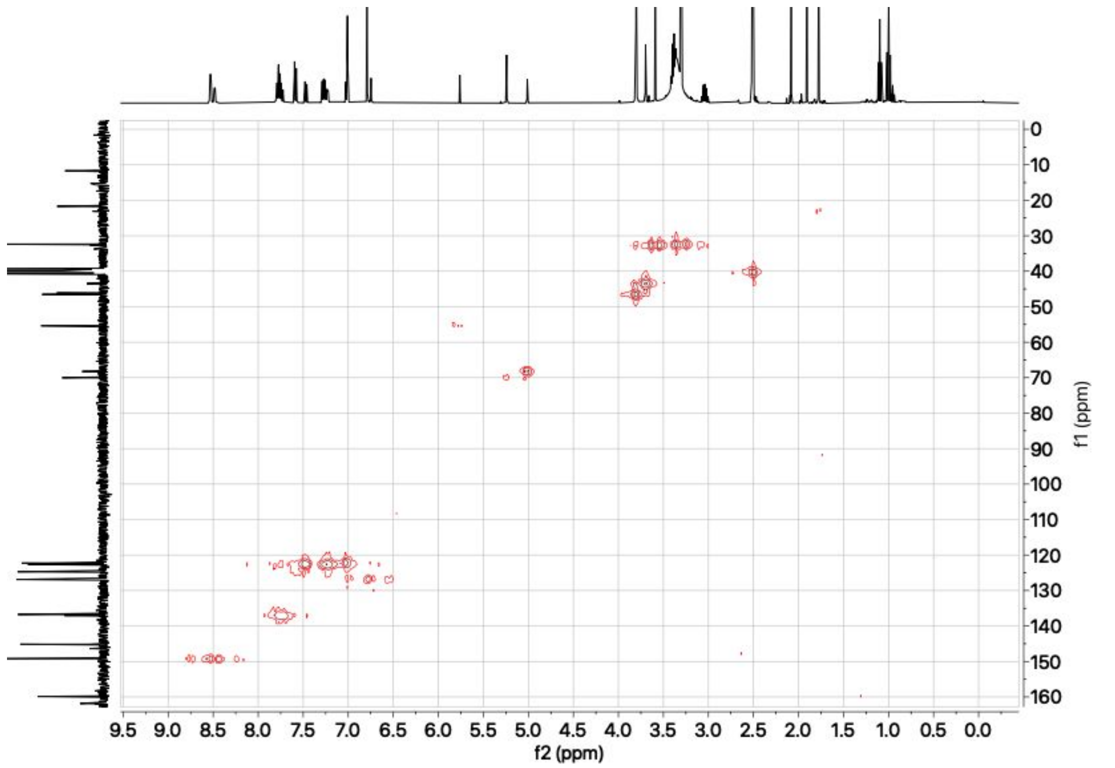

Figure S4. The HSQC NMR spectrum of ligand $\mathbf{L^2}$ in d_6-DMSO measured at 298 K.

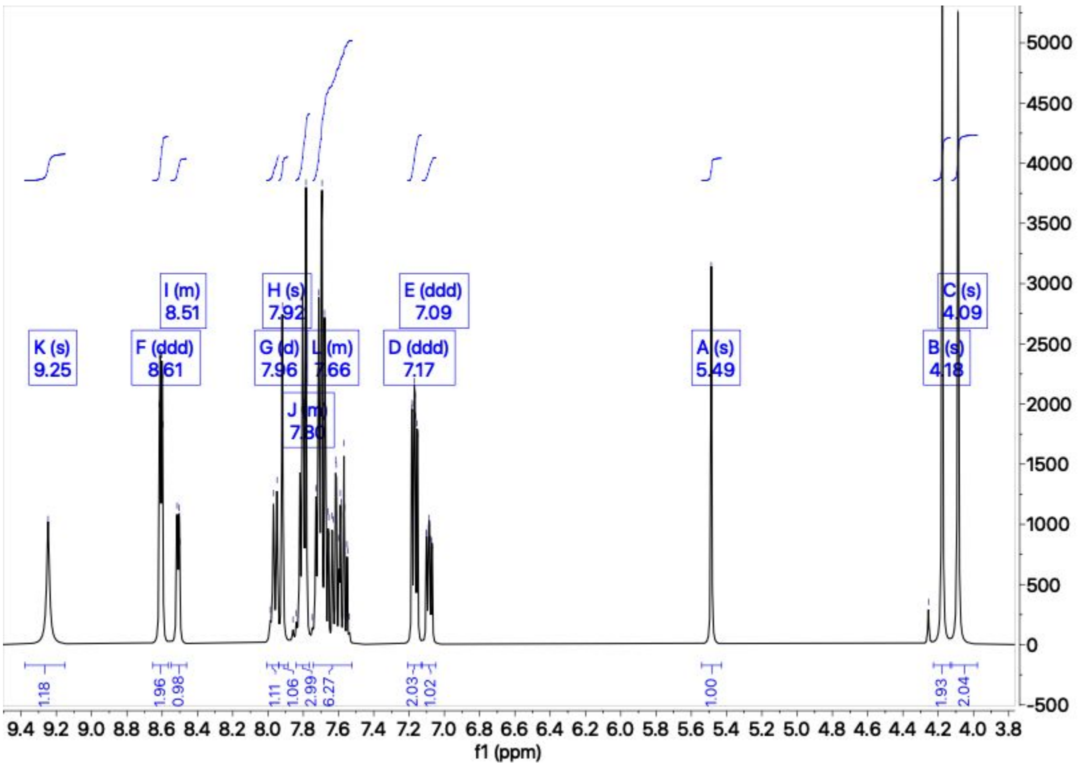

Figure S5. The ^{1}H NMR spectrum of ligand $\mathbf{L^3}$ in $CDCl_3$ (400 MHz) measured at 298 K.

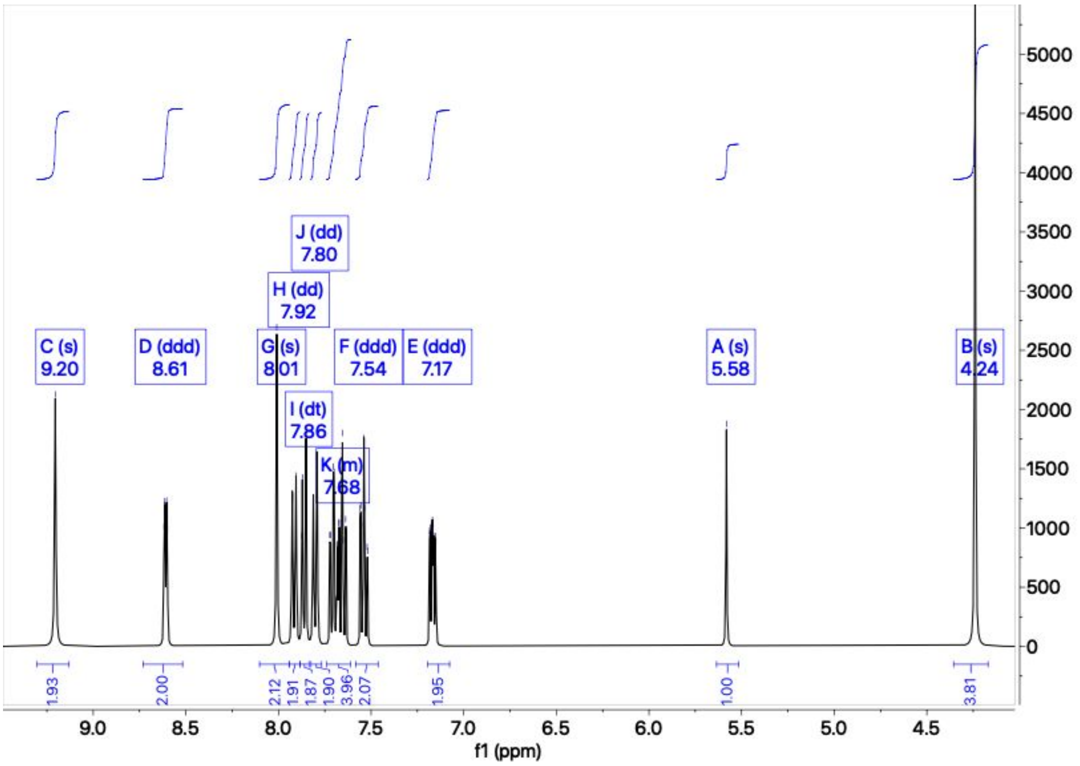

Figure S6. The ^{1}H NMR spectrum of ligand **4** in $CDCl_3$ (400 MHz) measured at 298 K.

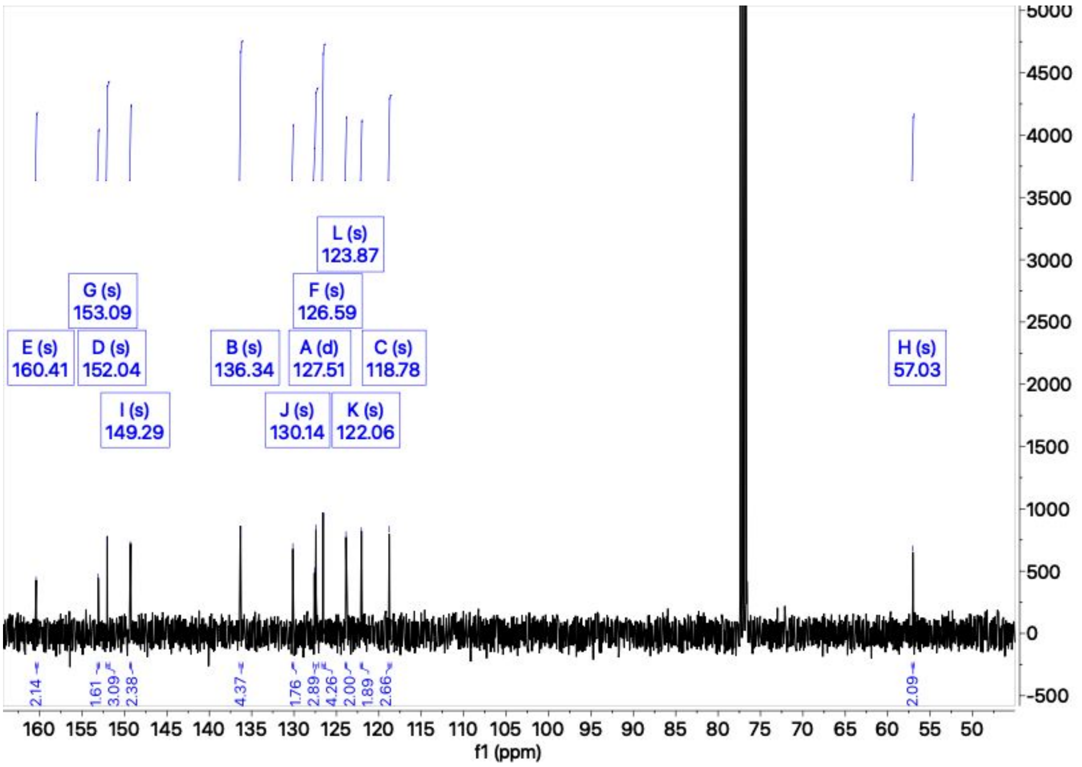

Figure S7. The ^{13}C NMR spectrum of ligand **4** in $CDCl_3$ (100 MHz) measured at 298 K

Synthesis of complexes

If not otherwise specified, the syntheses described below were performed at room temperature in a glovebox.

Synthesis of complexes 1a-4a·$(ClO_4)_2$

The Fe^{II} complexes **1a**·$(ClO_4)_2$, **2a**·$(ClO_4)_2$, **3a**·$(ClO_4)_2$, and **4a**·$(ClO_4)_2$ were prepared using the same method: 0.2 mmol of each ligand (**L^1/L^2/L^3/L^4**) was combined with one equivalent of $Fe(ClO_4)_2{\cdot}xH_2O$ (0.2 mmol) in 3ml anhydrous and degassed acetonitrile and the resultant mixture was stirred overnight at room temperature in a glovebox. After that, the concentrated solvent mixture was transferred into a test tube and the relevant Fe^{II} complex was crystallized by liquid layer diffusion of degassed diethyl ether into the concentrated solution inside a glove box, after which the complex could be isolated as an air-stable solid (yields: ~ 60%).

Synthesis of complexes 1a-4a·$(OTf)_2$

To a solution of 0.2 mmol of each ligand (**L^1/L^2/L^3/L^4**) in anhydrous and degassed acetonitrile (3 mL), one equivalent of $Fe(OTf)_2{\cdot}2MeCN$ was added. After stirring for overnight, the resultant mixture was transferred into a test tube and the relevant Fe^{II} complex was crystallized by liquid layer diffusion of degassed diethyl ether into the concentrated solution inside a glove box, after which the complex could be isolated as an air-stable solid (yields: ~ 55%).

Synthesis of 4a·$(BF_4)_2$

A solution of $Fe(BF_4)_2{\cdot}6H_2O$ (233mg, 0.69 mmol) in CH_3CN (3 mL) was added to a solution of ligand **L^4** (322 mg, 0.69 mmol) in CH_3CN (3 mL). The dark red solution was placed in an ethyl acetate bath and after 3 days the title compound (400 mg, 0.55 mmol, 80%) was isolated as dark-red crystals.

Synthesis and characterization of the dinuclear complex $[Fe^{III}(\mathbf{L}^2)_2(\mu\text{-}O)](OTf)_2$ (**5**·$(OTf)_2$)

A solution of **L^2** (0.2 mmol) was combined with a solution of $Fe(OTf)_2$ (0.2 mmol) in 3 mL of acetonitrile. The resultant mixture was allowed to stir for 3 h at room temperature and the dark-red reaction mixture was concentrated to ~2 mL by slow evaporation under ambient air. The complex was crystallized by liquid layer diffusion of diethyl ether into the concentrated solution, and then the complex was isolated as air-stable solids. Yield: 45%. X-ray crystallographic analysis confirmed the structure as $[Fe^{III}_2(\mathbf{L}^2)_2(\mu\text{-}O)]$ $(OTf)_2$ (Table S8).

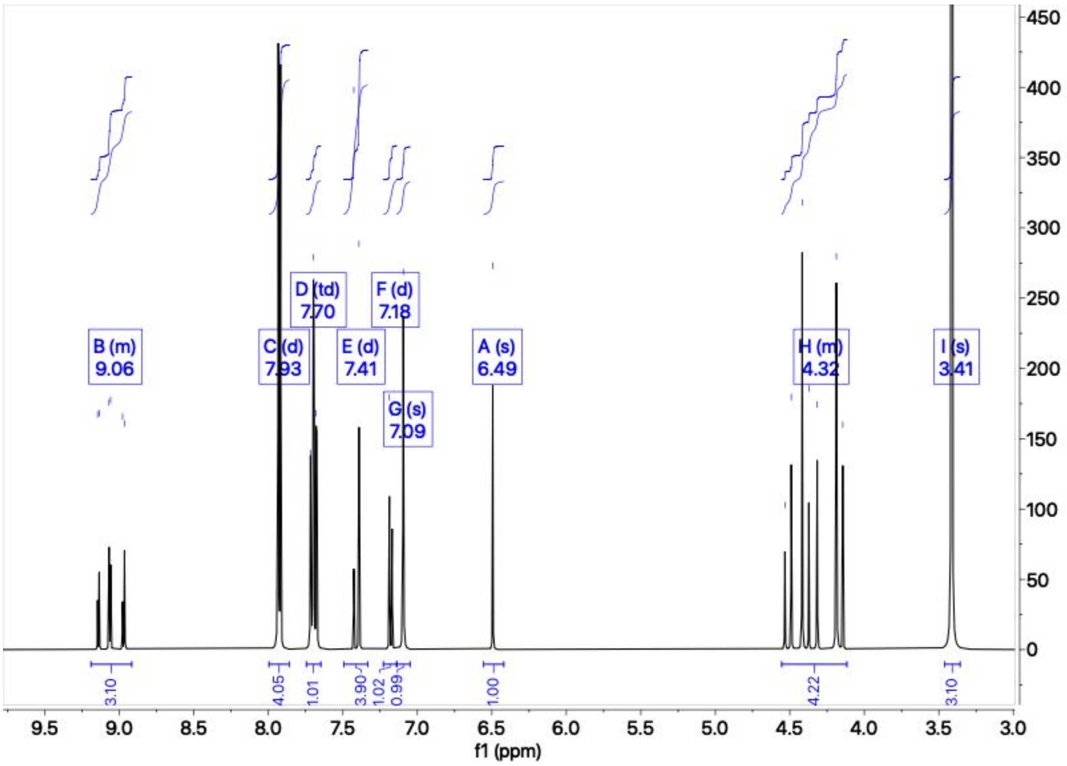

Figure S8. The ^{1}H NMR spectrum of complex **1a**·$(ClO_4)_2$ in CD_3CN measured at 298 K.

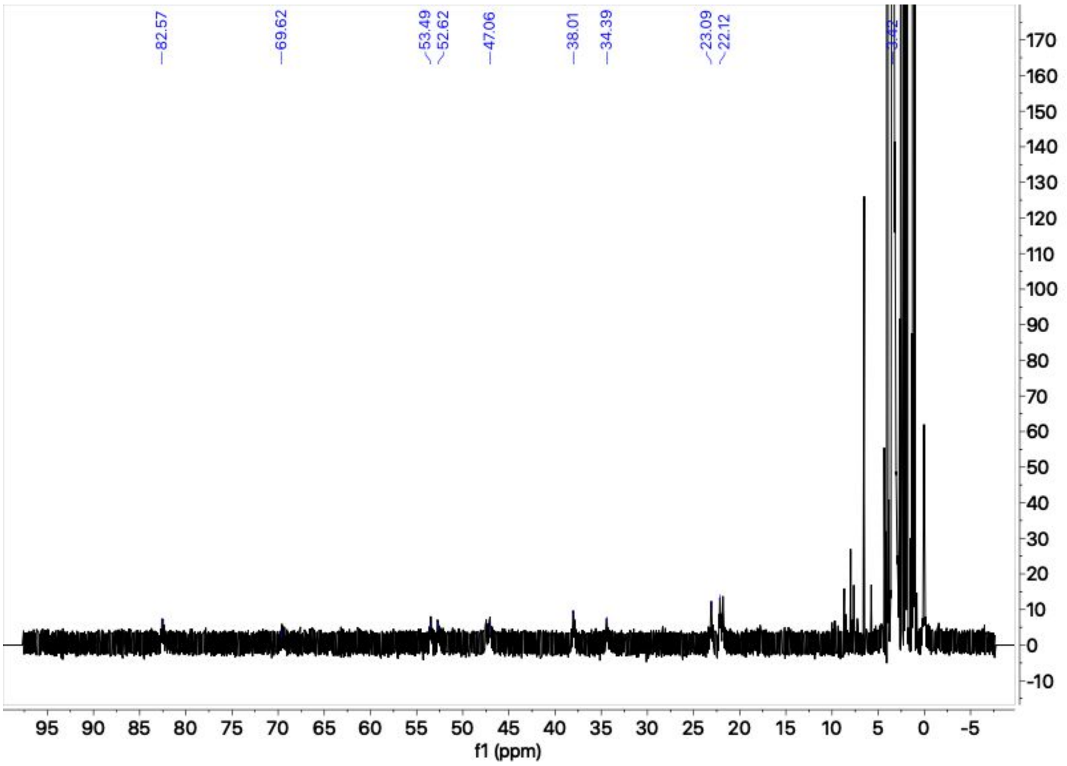

Figure S9. The ^{1}H NMR spectrum of complex **1a**·$(ClO_4)_2$ in d_6-DMSO measured at 298 K.

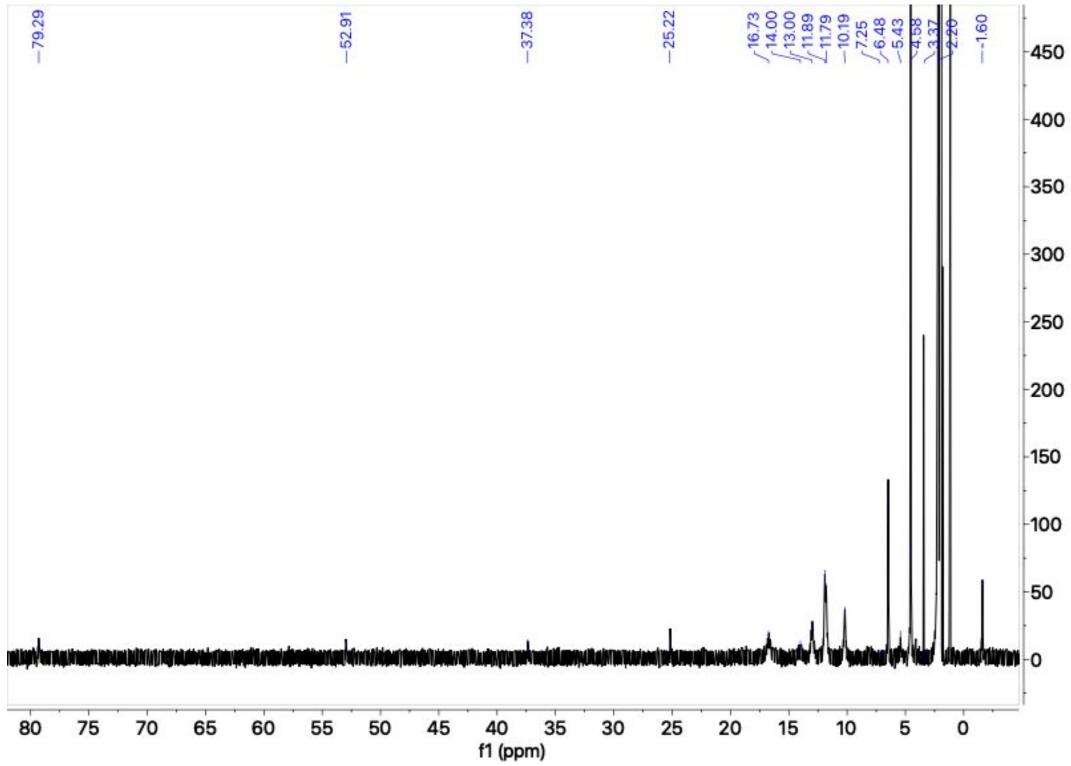

Figure S10. The ^{1}H NMR spectrum of complex **2a**·$(ClO_4)_2$ in CD_3CN measured at 298 K.

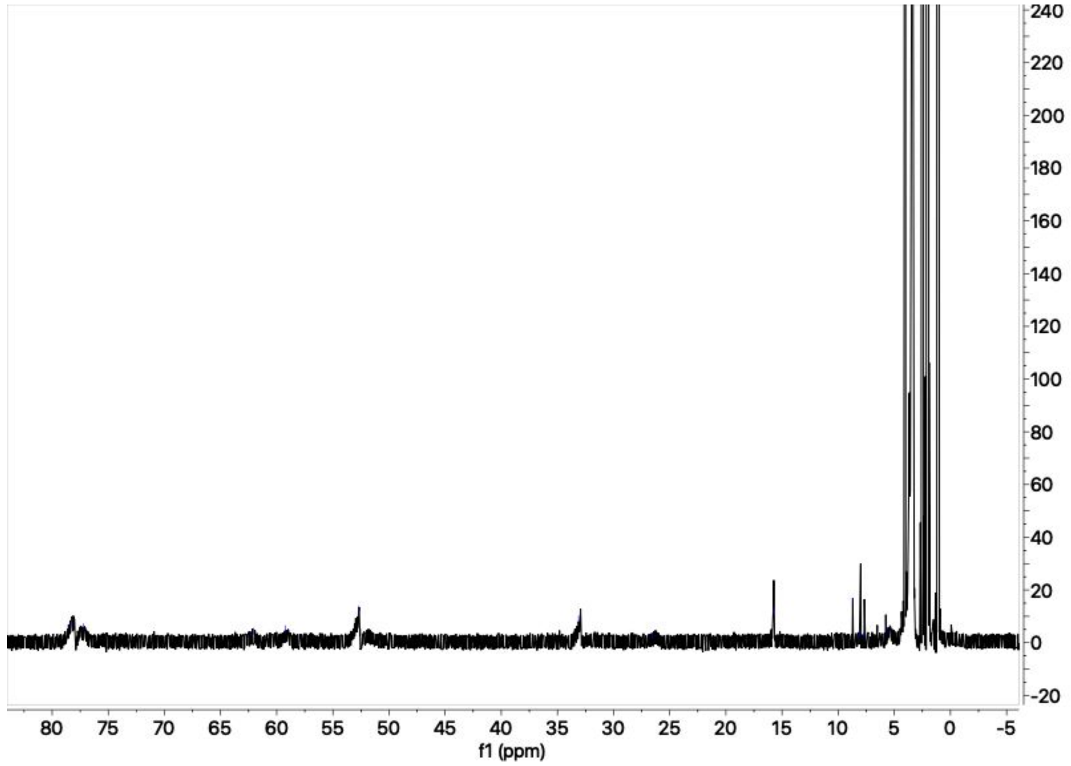

Figure S11. The ^{1}H NMR spectrum of complex **2a**·$(ClO_4)_2$ in d_6-DMSO measured at 298 K.

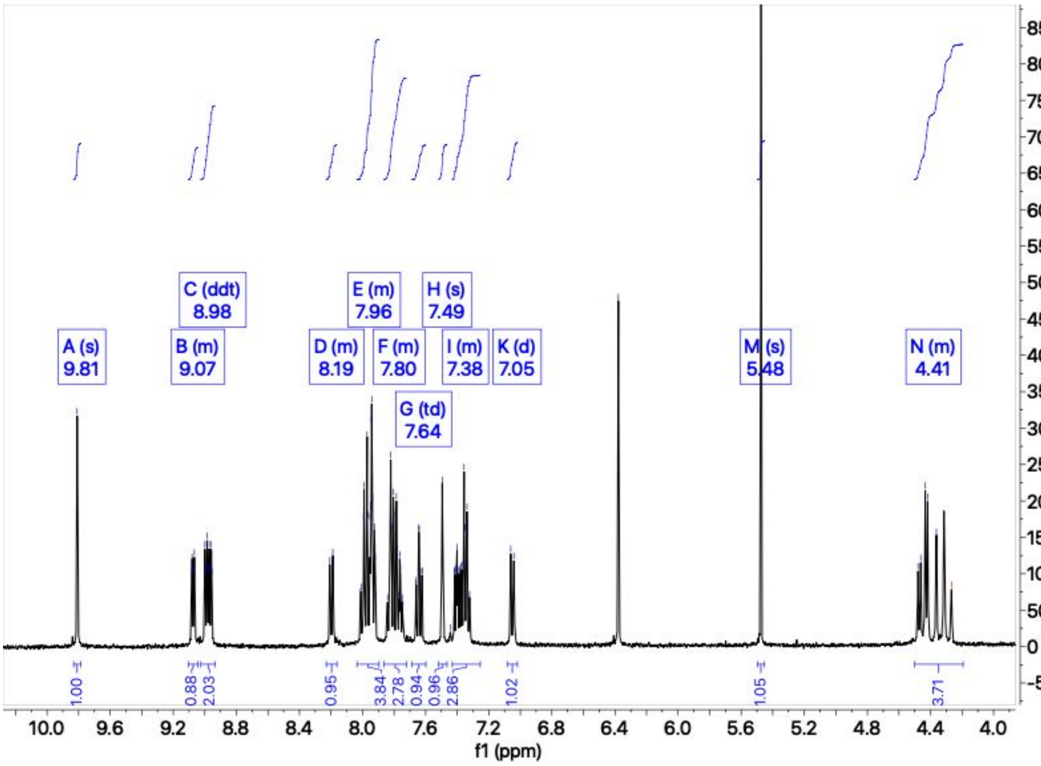

Figure S12. The ^{1}H NMR spectrum of complex **3a**·$(ClO_4)_2$ in CD_3CN measured at 298 K

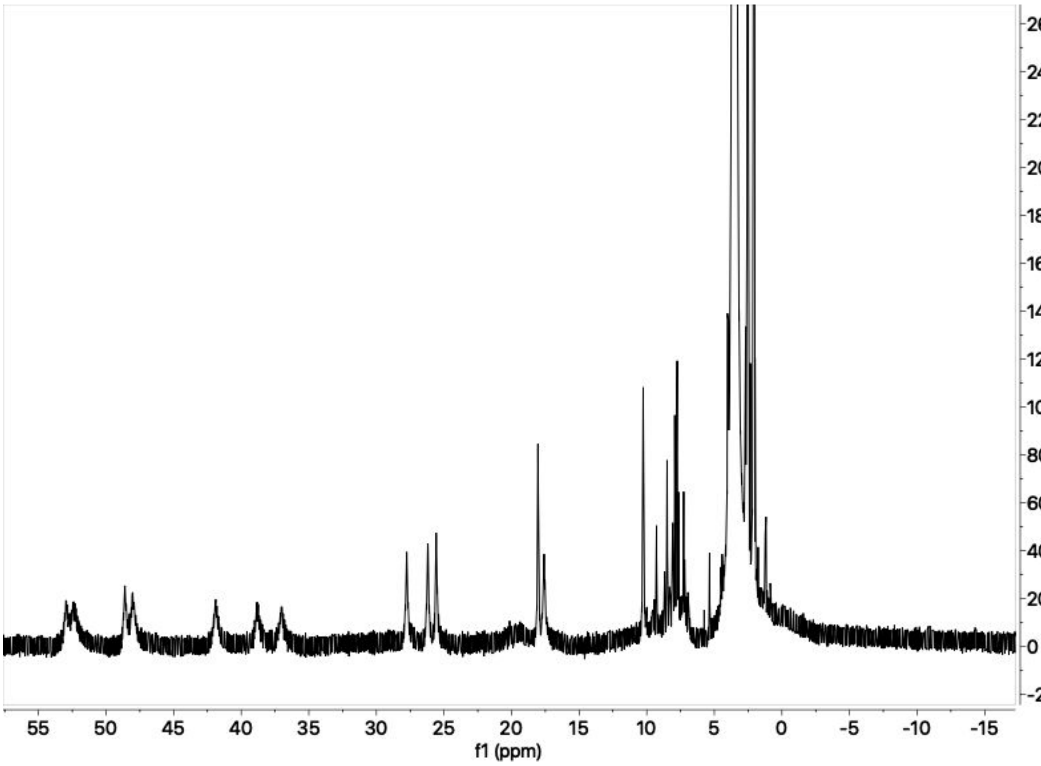

Figure S13. The ^{1}H NMR spectrum of complex **3a**·$(ClO_4)_2$ in d_6-DMSO measured at 298 K

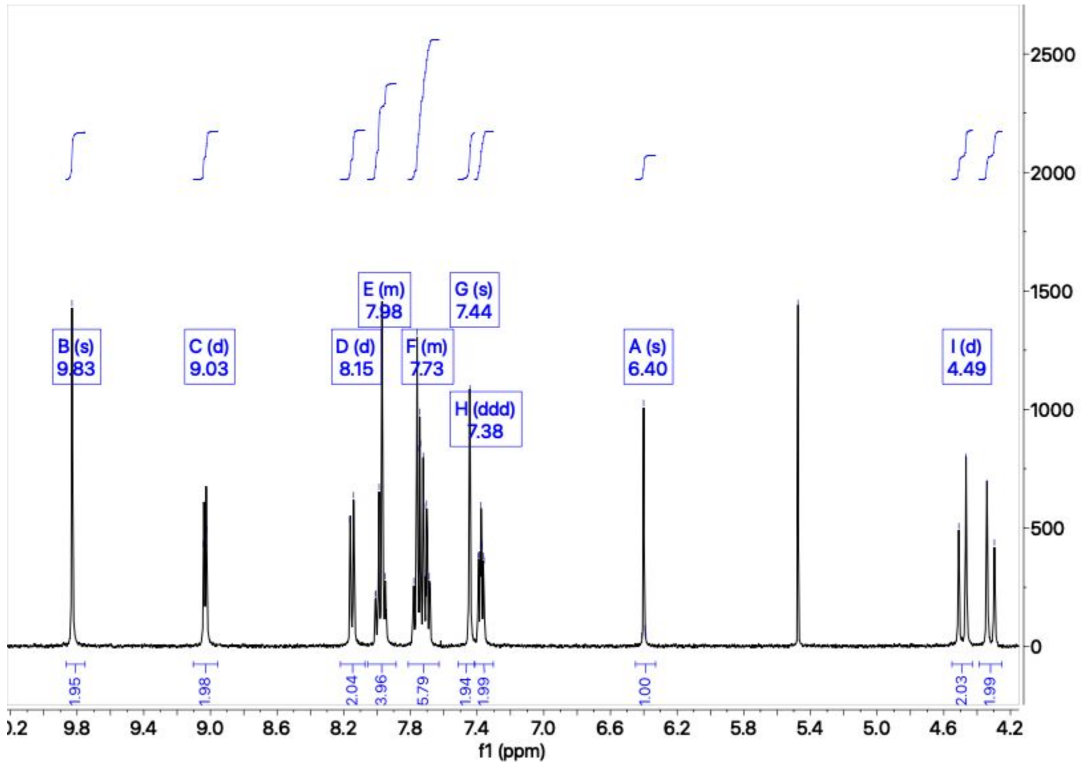

Figure S14. The ^{1}H NMR spectrum of complex **4a**·$(ClO_4)_2$ in CD_3CN measured at 298 K

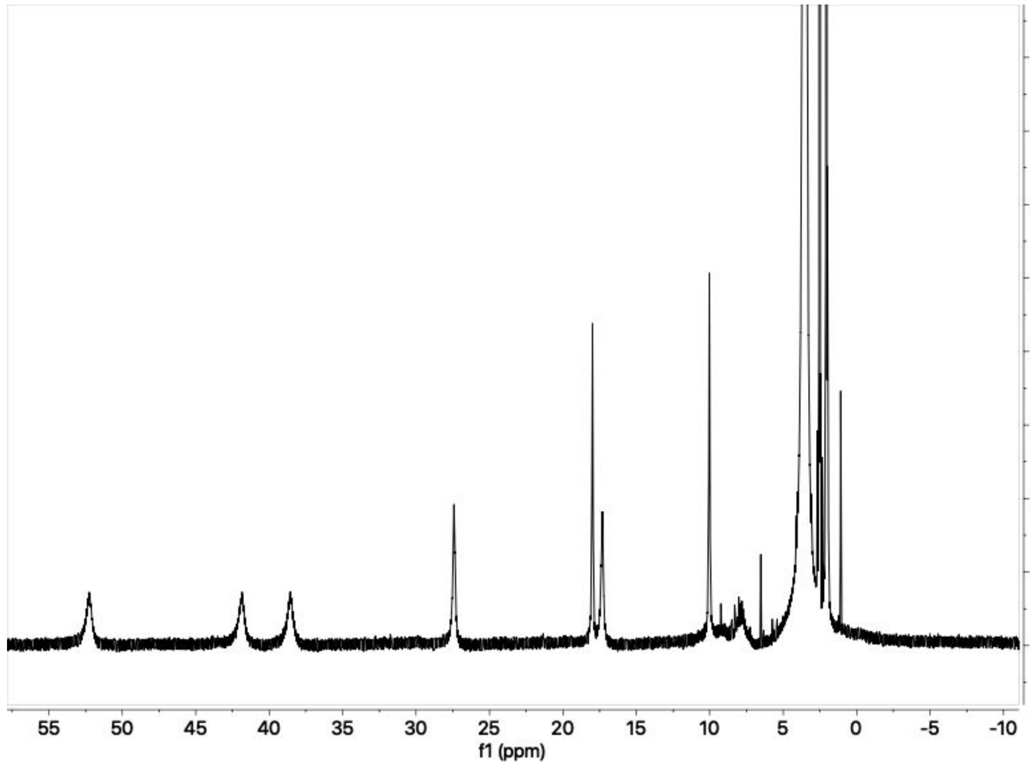

Figure S15. The ^{1}H NMR spectrum of complex **4a**·$(ClO_4)_2$ in d_6-DMSO measured at 298 K

Mössbauer spectroscopy. Zero-field Mössbauer spectra were recorded with a ^{57}Co source in a Rh matrix using an alternating constant acceleration *Wissel* Mössbauer spectrometer operated in the transmission mode and equipped with a *Janis* closed-cycle helium cryostat. Isomer shifts are given relative to iron metal at ambient temperature. Simulation of the experimental data was performed with the *Mfit* program using *Lorentzian* line doublets: E. Bill, Max-Planck Institute for Chemical Energy Conversion, Mülheim/Ruhr, Germany.

Magnetic field Mössbauer spectra were recorded with a ^{57}Co source in a Rh matrix using an alternating constant acceleration *Wissel* Mössbauer spectrometer operated in the transmission mode perpendicular to the γ-rays and equipped with a *OptiCool* closed-cycle helium cryostat from *Quantum Design*. Isomer shifts are given relative to iron metal at ambient temperature. Simulation of the experimental data was performed with the *MX* program by diagonalization of the spin Hamiltonian for the electronic and nuclear spins: E. Bill, Max-Planck Institute for Chemical Energy Conversion, Mülheim/Ruhr, Germany.

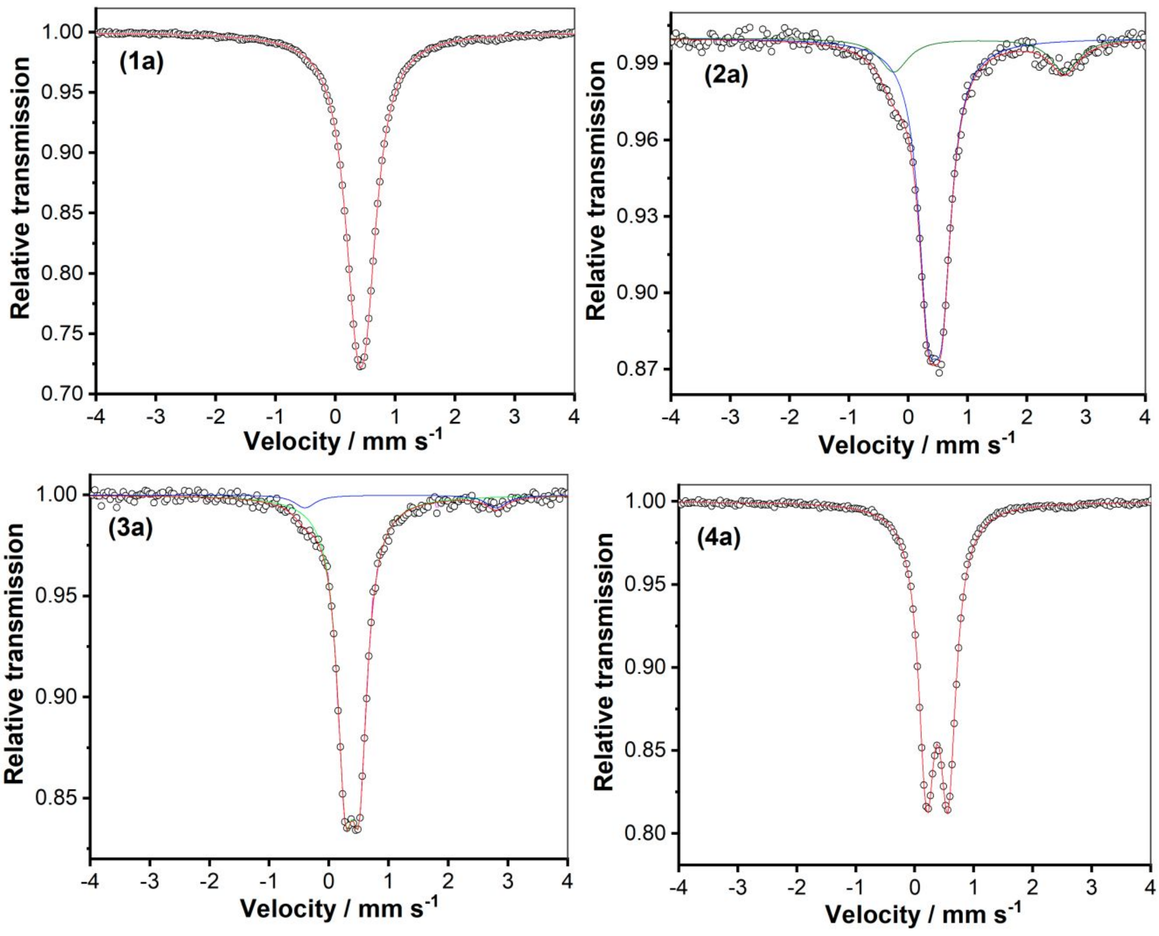

Figure S16. Zero-field Mössbauer spectra for solid samples of complexes **1a-4a**·$(OTf)_2$ at 80 K.

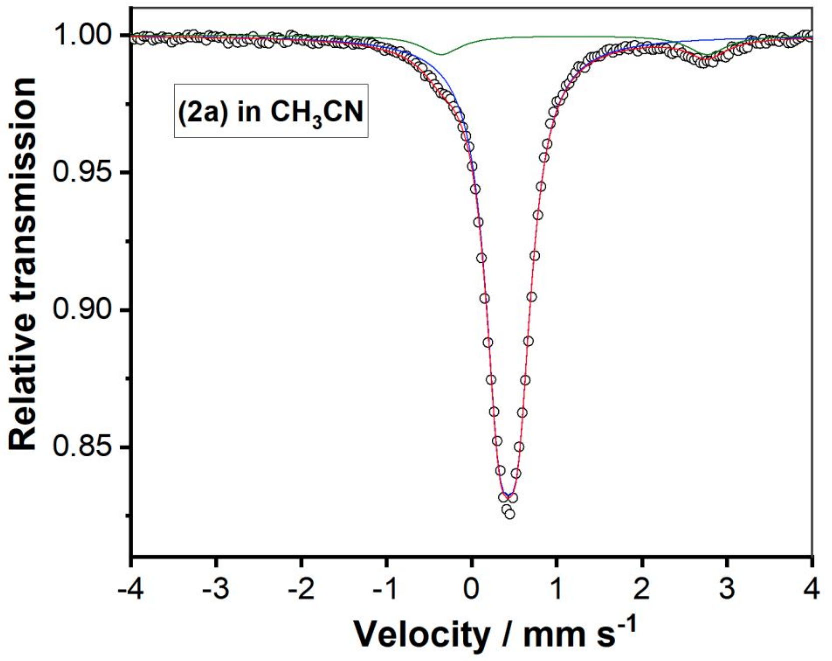

Figure S17. Zero-field Mössbauer spectrum of complex **2a**$\cdot(OTf)_2$ in frozen acetonitrile at 80 K.

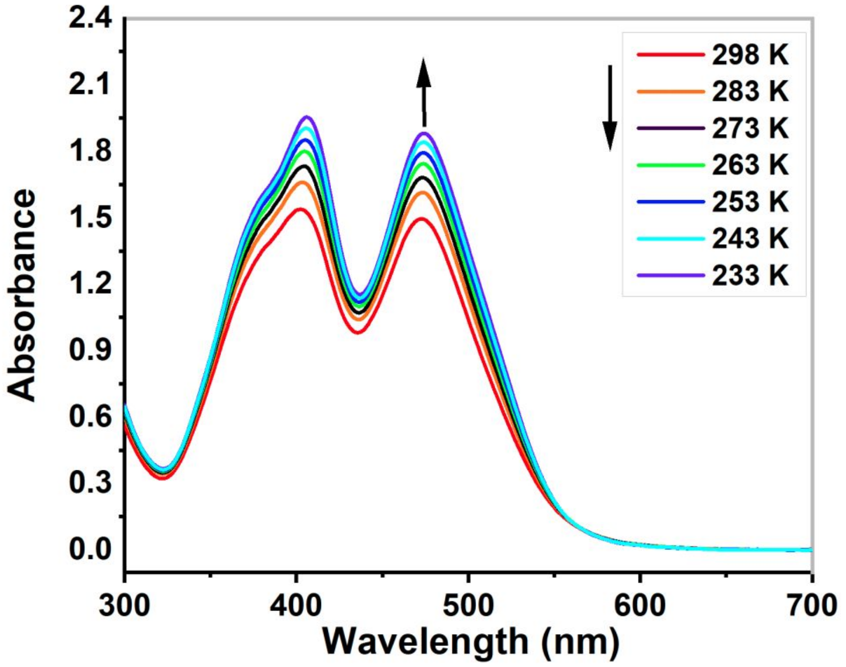

Figure S18. Variable temperature UV/Vis spectra of complex **2a**$\cdot(ClO_4)_2$ (0.5 mM) in liquid acetonitrile.

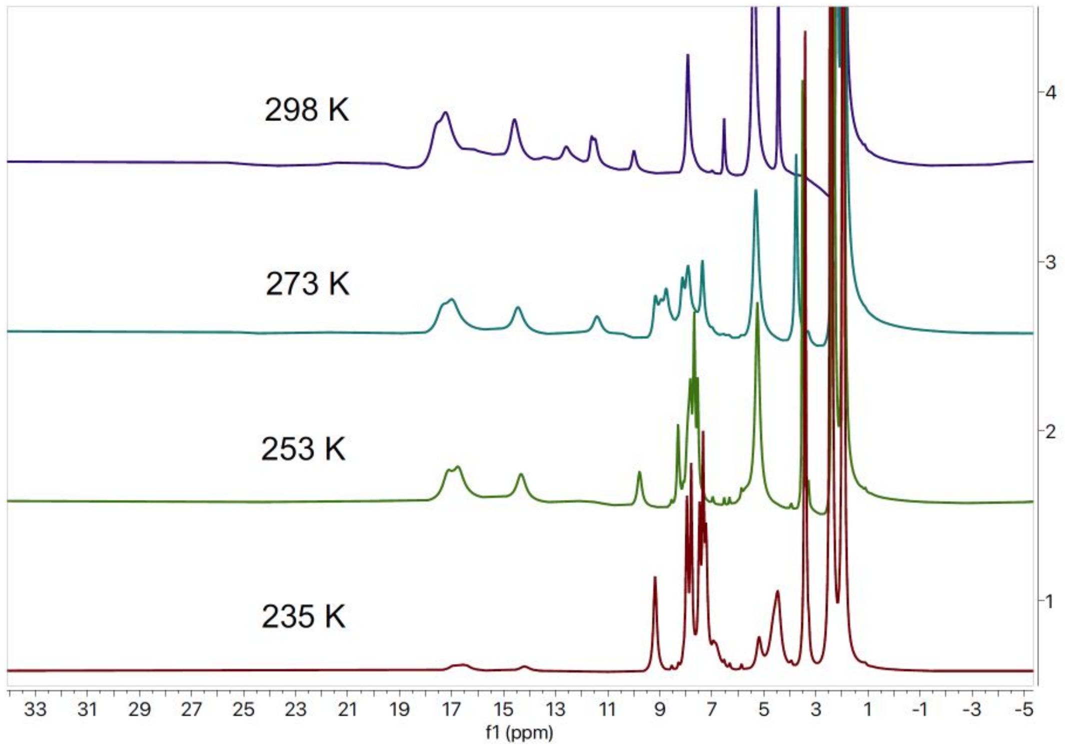

Figure S19. Variable temperature ^{1}H NMR spectra of complex **2a**·$(ClO_4)_2$ in CD_3CN measured from 298-235 K.

Table S1. Data for TD-DFT excitations within PBE0/Def2-TZVP(-f) level of theory for **4a**.

State[a]	Energy eV	Energy nm	*f*	Configuration (%)[b]	Attribuition[c]
S_1	2.431	510	7×10^{-6}	H-2 → L+10 (21)<br>H-1 → L+8 (36)	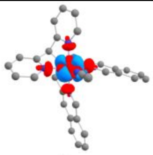
S_2	2.453	505	0.0011	H → L+10 (55)<br>H-1 → L+8 (27)	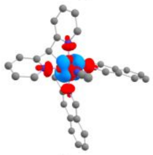
S_3	2.468	502	0.0008	H-1 → L+10 (20)<br>H-2 → L+8 (61)	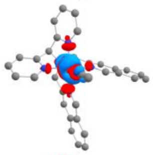
S_4	3.119	397	0.0022	H-1 → L (35)<br>H-1 → L+1 (32)<br>H-2 → L+10 (18)	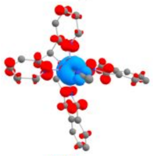
S_5	3.194	388	0.0136	H-2 → L (25)<br>H-2 → L+1 (32)	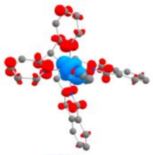
S_6	3.226	384	0.2326	H → L (34)<br>H → L+1 (52)	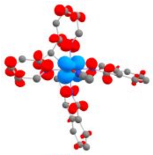
S_7	3.325	373	0.0031	H-2 → L+1 (11)<br>H → L (28)<br>H → L+1 (27)<br>H → L+8 (20)	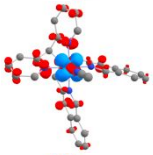
S_8	3.364	369	0.0002	H-1→ L (39)<br>H-1 → L+1 (54)	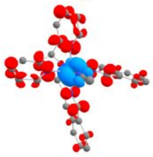
S_9	3.379	367	0.0262	H-2 → L (17)<br>H-2 → L+1 (14)<br>H → L (13)<br>H → L+8 (37)	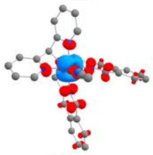
S_{10}	3.453	359	0.0054	H-2 → L (42)<br>H-2 → L+1 (21)<br>H → L+8 (11)	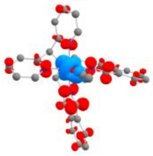

S_{11}	3.464	358	0.1375	H → L+2 (87)	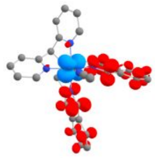
S_{12}	3.604	344	0.0109	H-1 → L+2 (53)	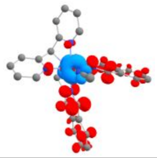

[a] Vertical states taking the ground state geometry as the reference; [b] Transitions with high percentage contributions are shown in parenthesis; [c] For the TD-DFT difference densities between ground and specified excited-state hydrogen were omitted for clarity. Blue indicates decreased, and red indicates increased electronic density.

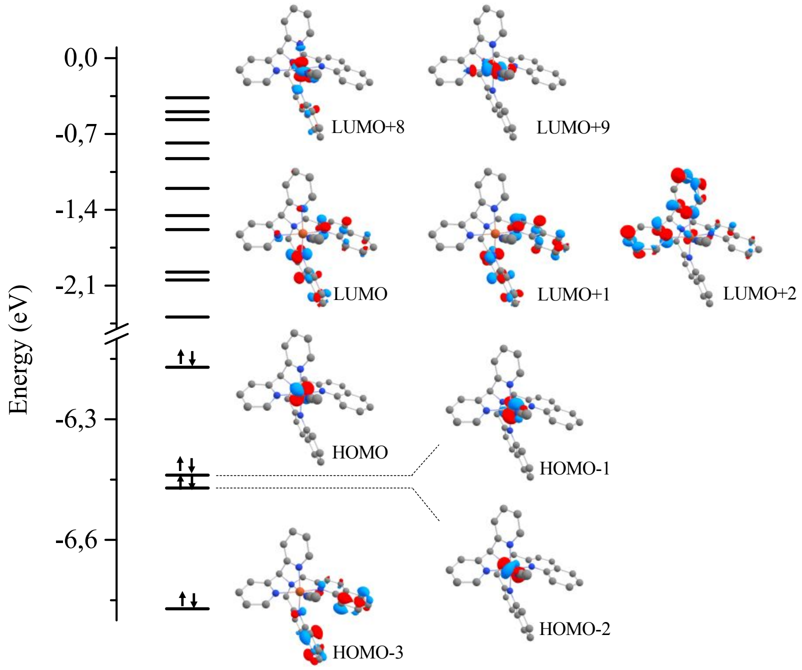

Figure. S20. Calculated frontier orbitals for $[Fe(N_2Py_2Qn2)(CH_3CN)]^{2+}$ within PBE0/Def2-TZVP(-f) level of theory.

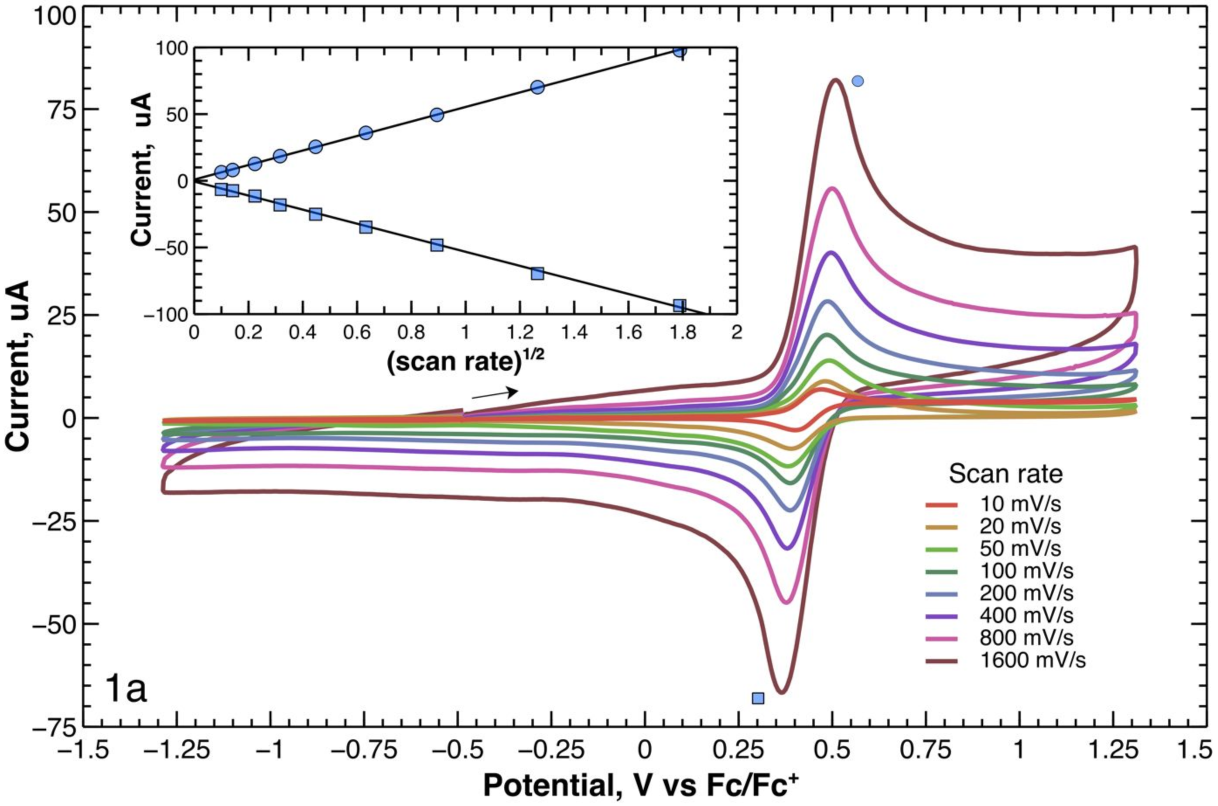

Figure S21. Cyclic voltammograms of **1a**·$(ClO_4)_2$ in acetonitrile at different scan rates.

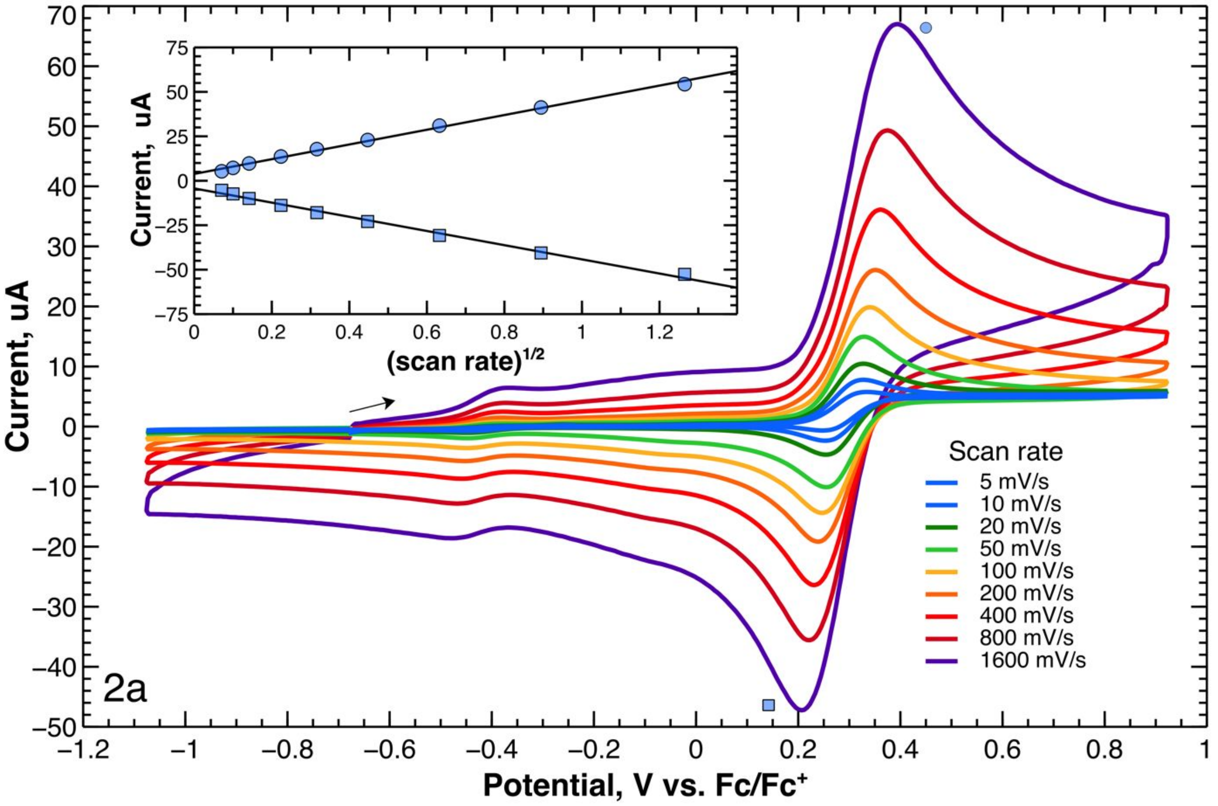

Figure S22. Cyclic voltammograms of **2a**·$(ClO_4)_2$ in acetonitrile at different scan rates. The very small impurity peak near -0.4V was observed on both glassy carbon and platinum working electrodes.

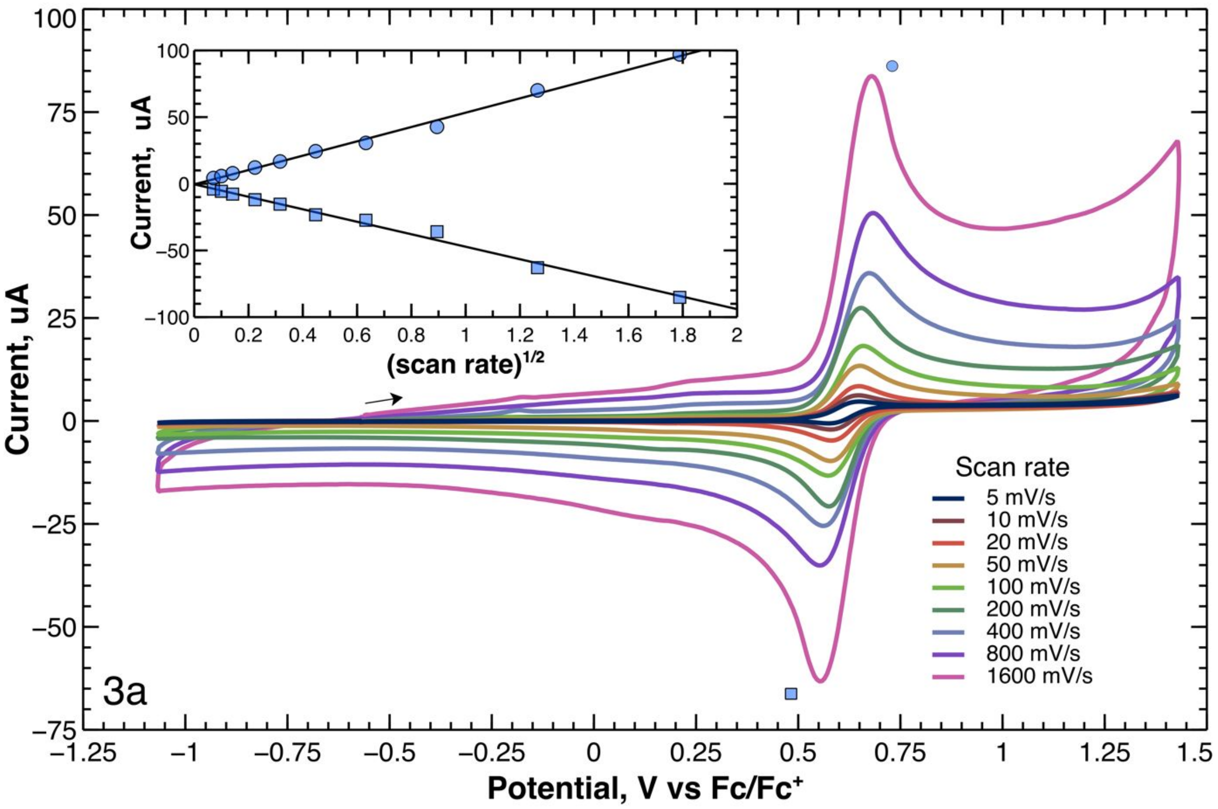

Figure S23. Cyclic voltammograms of **3a**·$(ClO_4)_2$ in acetonitrile at different scan rates.

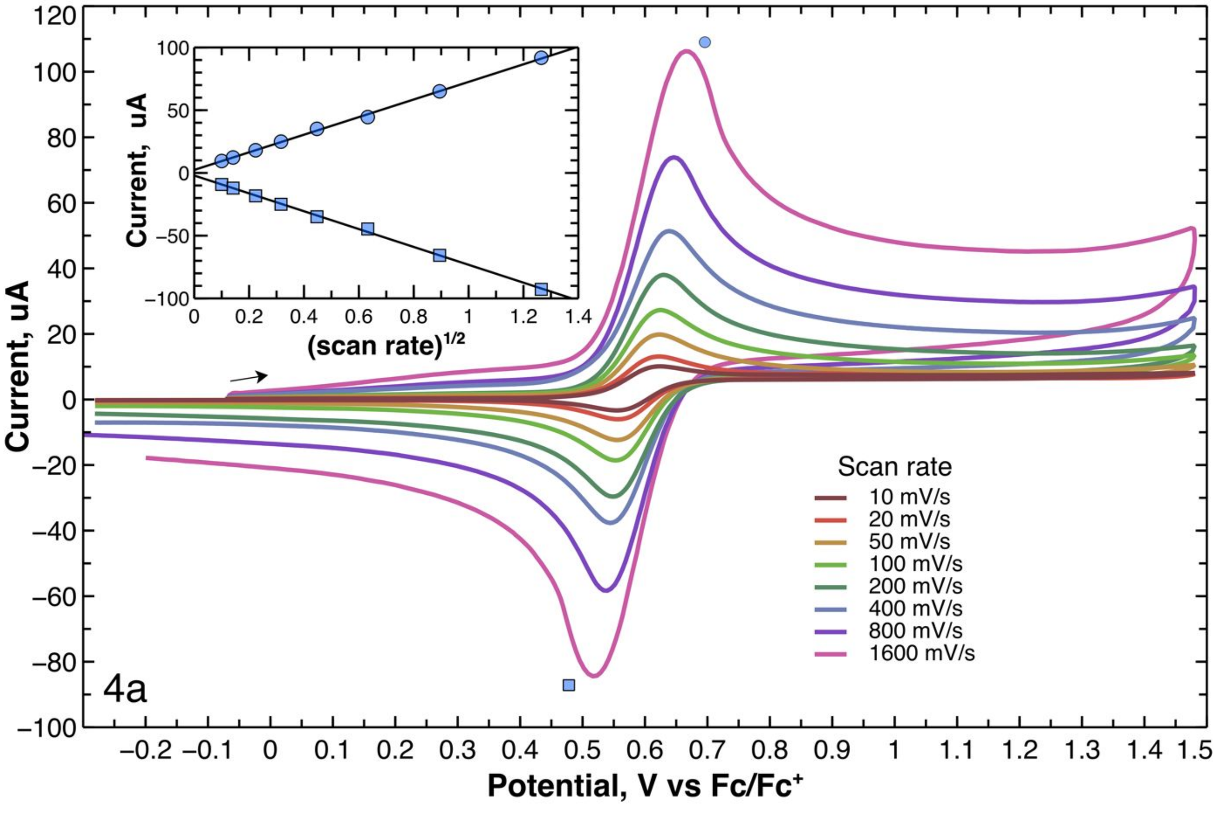

Figure S24. Cyclic voltammograms of **4a**·$(ClO_4)_2$ in acetonitrile at different scan rates.

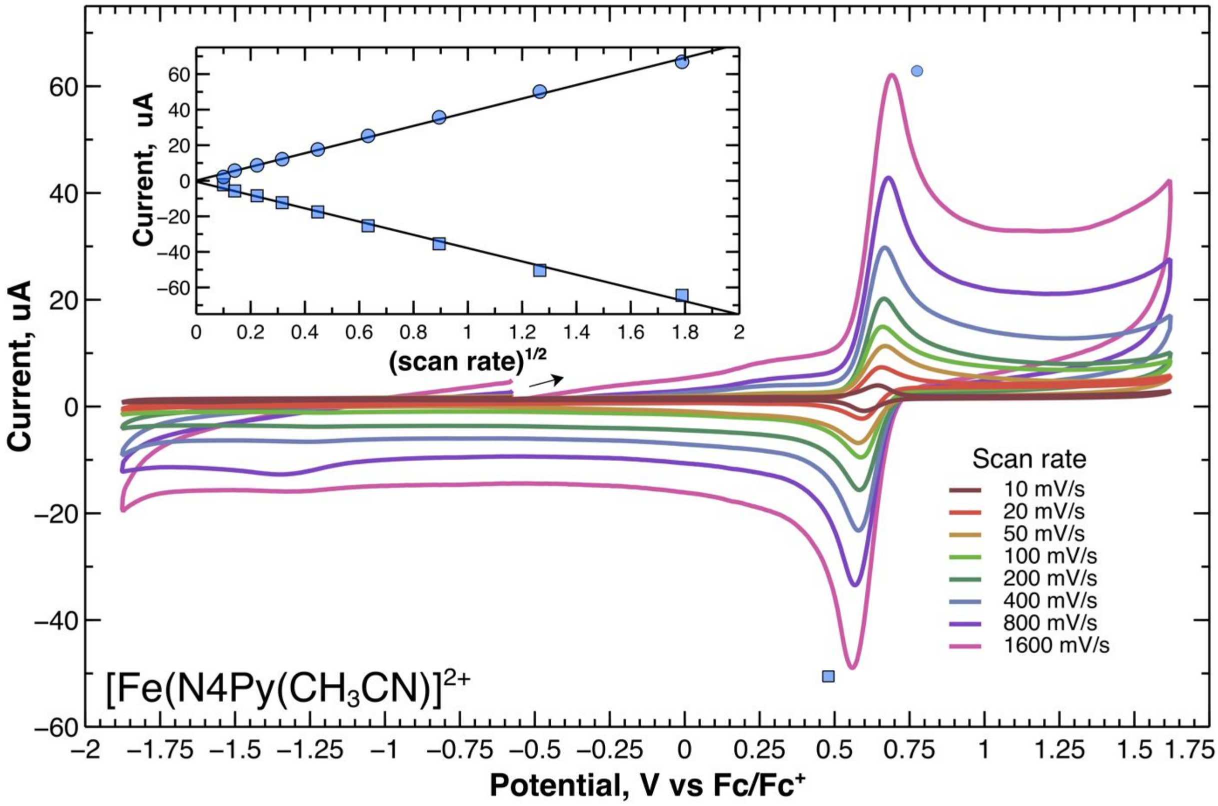

Figure S25. Cyclic voltammograms of $[Fe(N4Py)(CH_3CN)]^{2+}$ in acetonitrile at different scan rates.

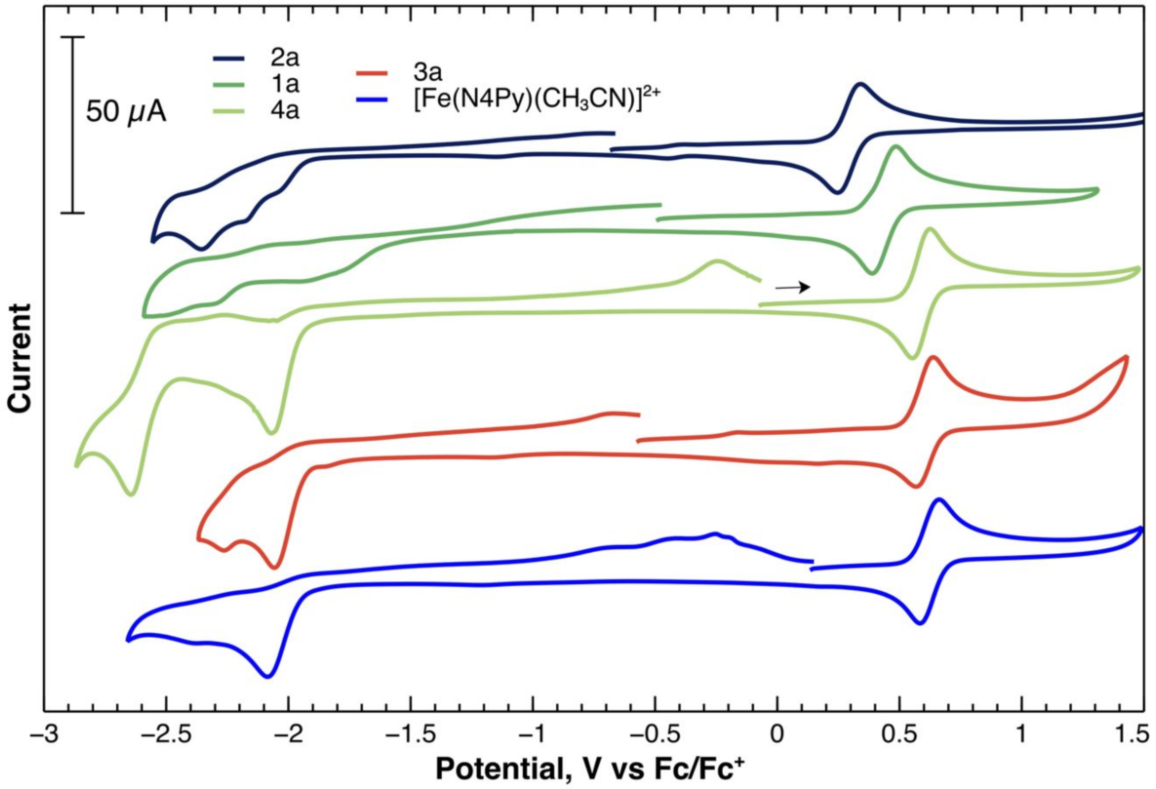

Figure S26. Extended cyclic voltammograms of **1a**-**4a**·$(ClO_4)_2$ and $[Fe(N4Py)(CH_3CN)](ClO_4)_2$ in acetonitrile at 100mV/s using a glassy carbon working electrode. All samples exhibit an irreversible reduction wave near -2.0 V vs Fc/Fc^+. The very small impurity peak near -0.4V was observed on both glassy carbon and platinum working electrodes.

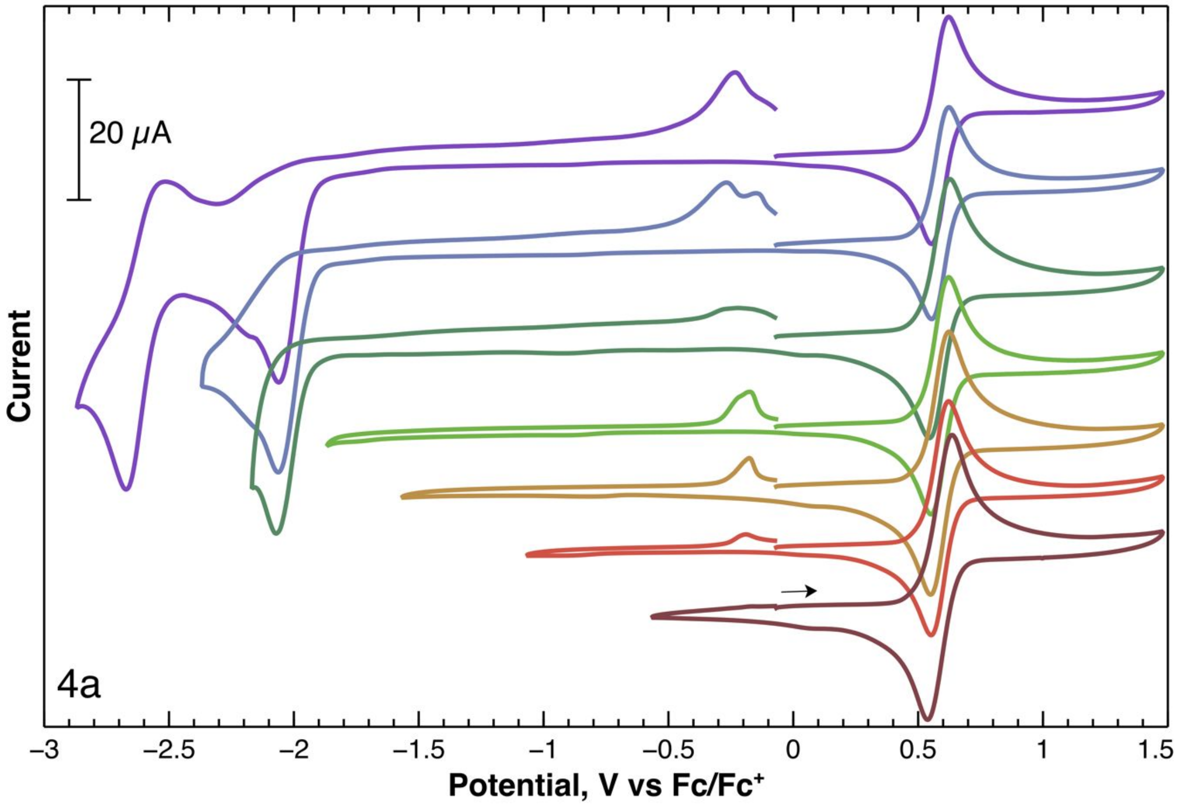

Figure S27. Cyclic voltammograms of **4a**·$(ClO_4)_2$ in acetonitrile at 50 mV/s with varying scan widths using a glassy carbon working electrode. Scanning negative, even if a reduction peak is not observed, results in irreversible oxidation peaks near -0.2 V vs Fc/Fc+. Similar results are observed for all the complexes, and can be observed in Figure S26 depending on the starting potential of the cyclic scan.

Magnetic susceptibility measurements. Temperature-dependent magnetic susceptibility measurements were carried out with a *Quantum-Design* MPMS3 SQUID magnetometer equipped with a 7 Tesla magnet in the range from 295 to 2.0 K at a magnetic field of 0.5 T. The powdered sample was contained in a polycarbonate capsule and fixed in a non-magnetic sample holder. Each raw data file for the measured magnetic moment was corrected for the diamagnetic contribution of the sample holder and the polycarbonate capsule. The molar susceptibility data were corrected for the diamagnetic contribution.

Simulation of the experimental magnetic data was performed with the *julX_2s* program: E. Bill, Max-Planck Institute for Chemical Energy Conversion, Mülheim/Ruhr, Germany.

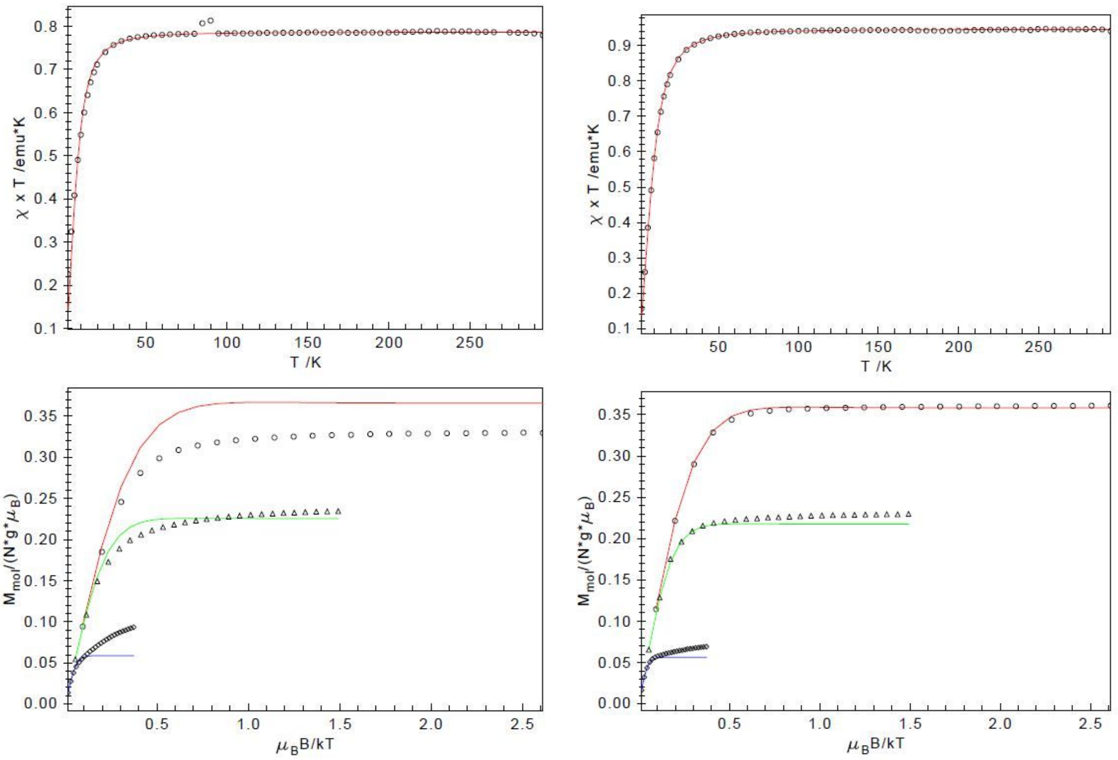

Figure S28. Magnetic susceptibility data (top) and variable-temperature variable-field measurements (bottom) at 7 T (circles), 4 T (triangles), and 1 T squares for the solid samples of Variable-temperature magnetic data for a solid sample of **3b**·$(ClO_4)_2$ (left) and **4b**·$(ClO_4)_2$ (right). The solid line represents the best curve fit with parameters g = 1.77, D =16.6 cm^{-1} for **3b**·$(ClO_4)_2$ and g = 1.95, D = 20.9 cm^{-1} for **4b**·$(ClO_4)_2$.

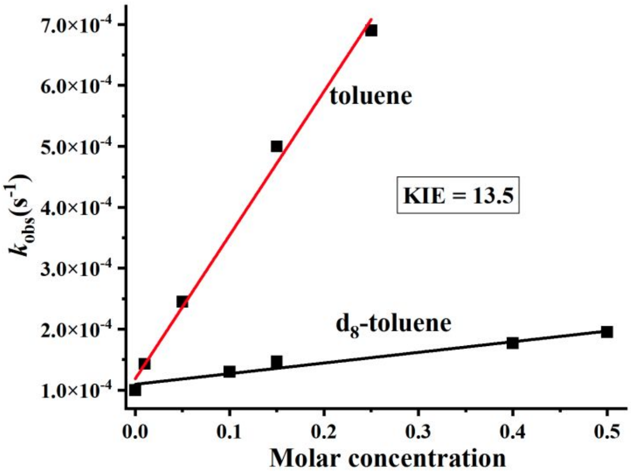

Figure S29. Determination of the kinetic isotope effect (KIE) for separate reactions with toluene and d_8-toluene with complex **4** at room temperature.

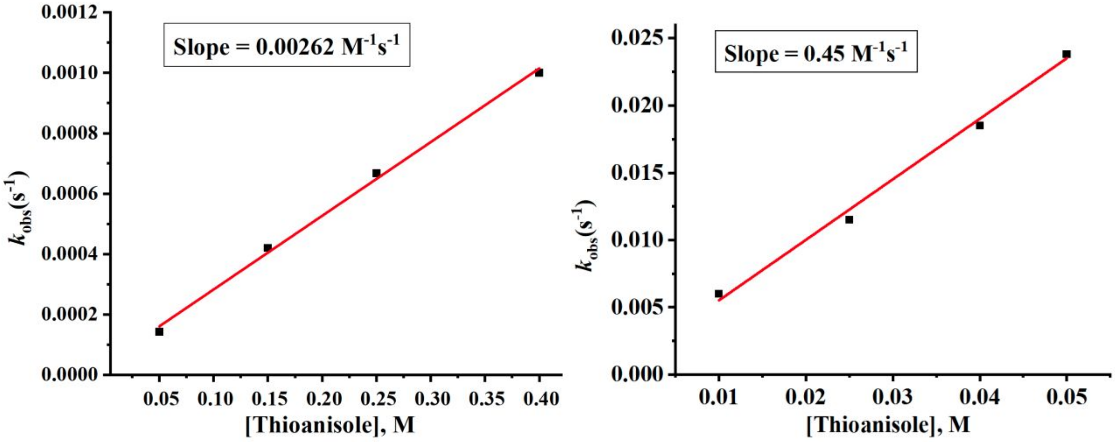

Figure S30. Second order rate constant for thioanisole oxidation by **1b** (left) and **2b** (right) at 243 K.

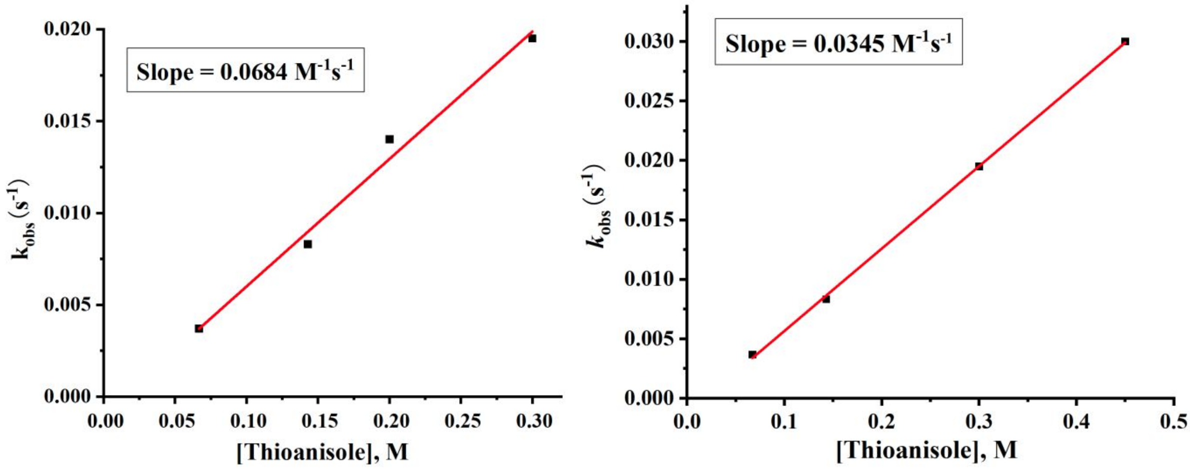

Figure S31. Second order rate constant for thioanisole oxidation by **3b** (left) and **4b** (right) at 273 K.

Table S2. Crystallographic data for complexes **1a**·$(OTf)_2$, **2a**·$(ClO_4)_2$, **3a**·$(OTf)_2$, **4a**·$(BF_4)_2$, **4b**·$(ClO_4)_2$ and $[Fe^{III}(\mathbf{L^2})_2(\mu\text{-}O)](OTf)_2$ (**5**·$(OTf)_2$).

**Compound**	**1a·(OTf)₂**	**2a·(ClO₄)₂**	**3a·(OTf)₂**	**4a·(BF₄)₂**	**4b·(ClO₄)₂**	**5·(OTf)₂**
Empirical formula	$C_{26}H_{25}F_6FeN_7O_6S_2$	$C_{50}H_{60}Cl_4Fe_2N_{18}O_{17}$	$C_{32}H_{27.5}F_6FeN_{6.5}O_6S_2$	$FeC_{33}H_{28}B_2F_8N_6$	$C_{31}H_{25}Cl_2FeN_5O_9$	$C_{54}H_{58}C_{10}F_{12}Fe_2N_{18}O_{13}S_4$
Temperature	100(2) K	120(2) K	100(2) K	293 K	295(2) K	120(2) K
Formula weight	765.50	1438.66	833.07	738.07	738.31	1635.12
Crystal system	Monoclinic	Monoclinic	Monoclinic	Monoclinic	Orthorhombic	Triclinic
Space group	$P2_1/n$	$P2_1/n$	$P2_1/c$	$P2_1/n$	Pbca	P-1
a, Å	12.4830(8)	11.9796(2)	14.0740(6)	10.7581(11)	16.723(3)	12.6166(2)
b, Å	19.3272(12) Å	40.4602(4)	23.2538(10)	21.653(3)	17.309(6)	13.3269(3)
c, Å	12.8998(9) Å	12.7365(2)	11.8054(5)	16.010(3)	20.4960(17)	23.3624(4)
α, deg	90	90	90	90	90	90.014(2)
β, deg	94.307(2)	94.3990(10)	114.6060(10)	106.510(14)	90	92.7140(10)
γ, deg	90.00	90.00	90.00	90	90	116.243(2)
Volume, Å3	3103.4(4)	6155.16(15)	3512.8(3)	3575.7(8)0)	5933(2)	3518.33(13)
Z	4	4	4	4	8	2
$D_{calcd.}$, Mg/m^3	1.638	1.552	1.575	1.371	1.653	1.543
μ Mo-Kα, mm^{-1}	0.710	6.094	0.634	0.496	0.521	0.634
F(000)	1560	2968	1700		3024	1672
θ range, deg	2.107-27.993	3.648-76.915	2.090-27.906		1.712-28.231	2.411-37.598
Reflections collected	91728	44048	68973		237949	124975
Reflections unique	7459	12823	8401	23967	9380	35684
R(int)	0.0596	0.0318	0.0640	0.1056	0.0694	0.0501
Data (I>2σ(I))	7459	12823	8401		9380	35684
Parameters refined	570	823	621	452	471	936
Goodness-of-fit on F^2	1.031	1.024	1.038		1.055	1.023
R_1 [I>2σ(I)]	0.0646	0.0562	0.0575	0.1078	0.0523	0.0584
wR_2	0.1622	0.1452	0.1338	0.3018	0.1489	0.1494
CCDC number	2241871	2241872	2241873	1883453	2241875	2241874

Table S3. Selected bond distances (Å) and bond angles (º) of complex **1a**

Fe(1)-N(4B)	1.87(2)
Fe(1)-N(4A)	1.875(19)
Fe(1)-N(7)	1.924(3)
Fe(1)-N(6)	1.964(3)
Fe(1)-N(5)	1.967(3)
Fe(1)-N(1)	1.987(3)
Fe(1)-N(2B)	2.039(19)
Fe(1)-N(2A)	2.049(18)
N(4B)-Fe(1)-N(7)	96.0(8)
N(4A)-Fe(1)-N(7)	94.4(7)
N(4B)-Fe(1)-N(6)	89.9(9)
N(4A)-Fe(1)-N(6)	168.9(7)
N(7)-Fe(1)-N(6)	96.62(11)
N(4B)-Fe(1)-N(5)	167.4(8)
N(4A)-Fe(1)-N(5)	90.9(8)
N(7)-Fe(1)-N(5)	96.43(12)
N(6)-Fe(1)-N(5)	86.84(11)
N(4B)-Fe(1)-N(1)	84.8(8)
N(4A)-Fe(1)-N(1)	85.8(7)
N(7)-Fe(1)-N(1)	179.16(12)
N(6)-Fe(1)-N(1)	83.15(11)
N(5)-Fe(1)-N(1)	82.75(11)
N(4B)-Fe(1)-N(2B)	90.2(8)
N(7)-Fe(1)-N(2B)	96.7(8)
N(6)-Fe(1)-N(2B)	166.6(8)
N(5)-Fe(1)-N(2B)	90.2(8)
N(1)-Fe(1)-N(2B)	83.5(8)
N(4A)-Fe(1)-N(2A)	90.4(8)
N(7)-Fe(1)-N(2A)	96.7(8)
N(6)-Fe(1)-N(2A)	89.3(7)
N(5)-Fe(1)-N(2A)	166.7(7)
N(1)-Fe(1)-N(2A)	84.1(8)

Table S4. Selected bond distances (Å) and bond angles (º) of complex **2a**

Fe(1)-N(8)	1.920(2)
Fe(1)-N(4)	1.956(2)
Fe(1)-N(5)	1.957(2)
Fe(1)-N(6)	1.963(2)
Fe(1)-N(1)	1.966(3)
Fe(1)-N(3)	2.013(2)
N(8)-Fe(1)-N(4)	96.81(10)
N(8)-Fe(1)-N(5)	97.09(10)
N(4)-Fe(1)-N(5)	87.36(10)
N(8)-Fe(1)-N(6)	95.86(10)
N(4)-Fe(1)-N(6)	167.17(10)
N(5)-Fe(1)-N(6)	89.08(10)
N(8)-Fe(1)-N(1)	95.83(11)
N(4)-Fe(1)-N(1)	89.99(10)
N(5)-Fe(1)-N(1)	167.03(10)
N(6)-Fe(1)-N(1)	90.74(10)
N(8)-Fe(1)-N(3)	179.48(10)
N(4)-Fe(1)-N(3)	82.69(10)
N(5)-Fe(1)-N(3)	82.74(10)
N(6)-Fe(1)-N(3)	84.63(10)
N(1)-Fe(1)-N(3)	84.33(10)
Fe(1B)-N(8B)	1.920(2)
Fe(1B)-N(4B)	1.959(2)
Fe(1B)-N(5B)	1.962(3)
Fe(1B)-N(1B)	1.967(3)
Fe(1B)-N(6B)	1.968(3)
Fe(1B)-N(3B)	2.014(2)
N(8B)-Fe(1B)-N(4B)	96.34(10)
N(8B)-Fe(1B)-N(5B)	97.26(10)
N(4B)-Fe(1B)-N(5B)	86.46(10)
N(8B)-Fe(1B)-N(1B)	95.51(10)
N(4B)-Fe(1B)-N(1B)	89.94(10)
N(5B)-Fe(1B)-N(1B)	167.04(10)
N(8B)-Fe(1B)-N(6B)	96.98(11)
N(4B)-Fe(1B)-N(6B)	166.54(10)
N(5B)-Fe(1B)-N(6B)	89.96(10)
N(1B)-Fe(1B)-N(6B)	90.70(11)
N(8B)-Fe(1B)-N(3B)	179.05(11)
N(4B)-Fe(1B)-N(3B)	82.86(10)

N(5B)-Fe(1B)-N(3B)	83.21(10)
N(1B)-Fe(1B)-N(3B)	83.99(10)
N(6B)-Fe(1B)-N(3B)	83.84(10)

Symmetry transformations used to generate equivalent atoms:

#1 -x+2,-y+1,-z+1

Table S5. Selected bond distances (Å) and bond angles (º) of complex **3a**

Fe(1)-N(6)	1.919(3)
Fe(1)-N(5)	1.953(3)
Fe(1)-N(2)	1.963(2)
Fe(1)-N(3)	1.964(3)
Fe(1)-N(1)	1.965(2)
Fe(1)-N(4)	1.970(2)
N(6)-Fe(1)-N(5)	94.88(11)
N(6)-Fe(1)-N(2)	95.67(10)
N(5)-Fe(1)-N(2)	90.75(10)
N(6)-Fe(1)-N(3)	96.90(11)
N(5)-Fe(1)-N(3)	168.17(11)
N(2)-Fe(1)-N(3)	86.93(10)
N(6)-Fe(1)-N(1)	177.74(11)
N(5)-Fe(1)-N(1)	83.68(10)
N(2)-Fe(1)-N(1)	86.11(10)
N(3)-Fe(1)-N(1)	84.59(11)
N(6)-Fe(1)-N(4)	95.76(10)
N(5)-Fe(1)-N(4)	86.89(10)
N(2)-Fe(1)-N(4)	168.48(10)
N(3)-Fe(1)-N(4)	93.08(10)
N(1)-Fe(1)-N(4)	82.43(10)

Symmetry transformations used to generate equivalent atoms:

Table S6. Selected bond distances (Å) and bond angles (º) of complex **4a**

Fe(1)-N(1)	1.968(5)
Fe(1)-N(2)	1.969(4)
Fe(1)-N(3)	1.977(5)
Fe(1)-N(4)	1.986(5)
Fe(1)-N(5)	1.984(4)
Fe(1)-N(6)	1.943(6)
N(1)-Fe-N(2)	91.5(2)
N(1)-Fe-N(3)	84.7(2)
N(1)-Fe-N(5)	92.8(2)
N(1)-Fe-N(6)	97.7(2)
N(2)-Fe-N(3)	86.8(2)
N(2)-Fe-N(4)	86.5(2)
N(2)-Fe-N(6)	95.4(2)
N(3)-Fe-N(4)	83.0(2)
N(3)-Fe-N(5)	82.5(2)
N(4)-Fe-N(5)	86.9(2)
N(4)-Fe-N(6)	94.6(2)
N(5)-Fe-N(6)	95.0(2)
N(3)-Fe-N(6)	176.961(1)

High resolution mass spectra (HRMS) of iron complexes

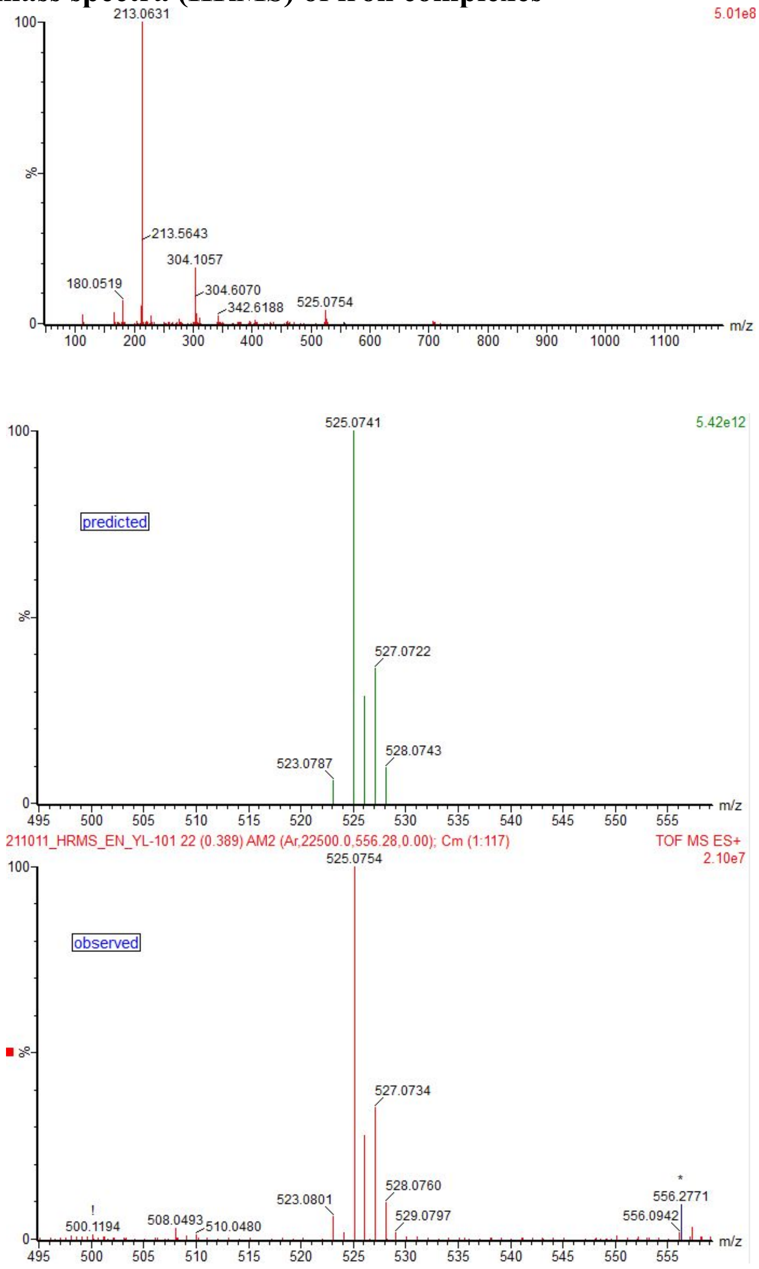

Figure S32. The HRMS of **1a**·$(ClO_4)_2$ in acetonitrile. The peaks at *m/z* = 213.0631 and *m/z* = 525.0754 correspond to the formulations $[Fe^{II}(\mathbf{L^1})]^{2+}$ (calc. 213.0628) and $[Fe^{II}(\mathbf{L^1})(ClO_4)]^{+}$ (calc. 525.0741), respectively.

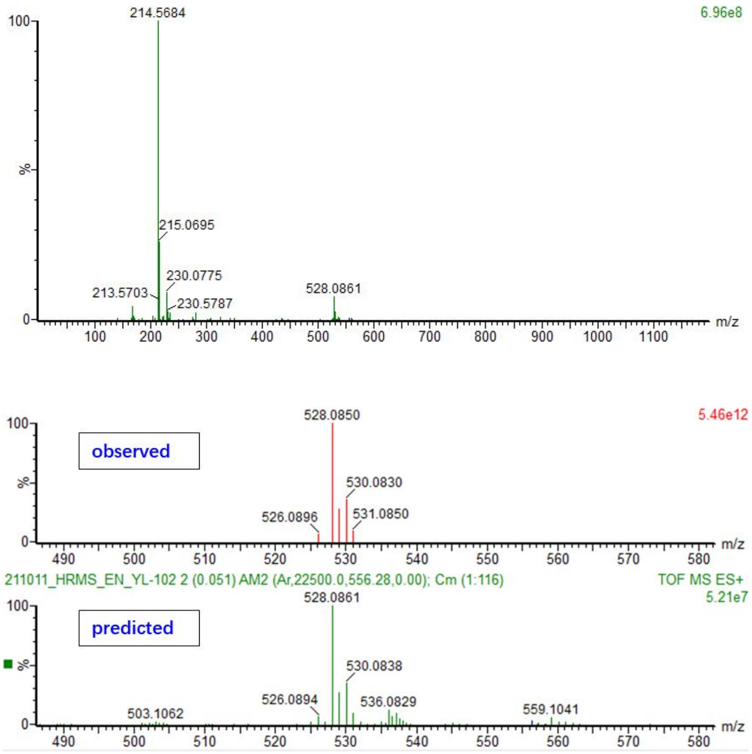

Figure S33. The HRMS of **2a**·$(ClO_4)_2$ in acetonitrile. The peaks at *m/z* = 214.5684 and *m/z* = 528.0861 correspond to the formulations $[Fe^{II}(\mathbf{L^2})]^{2+}$ (calc. 214.5628) and $[Fe^{II}(\mathbf{L^2})(ClO_4)]^{+}$ (calc. 528.0850), respectively.

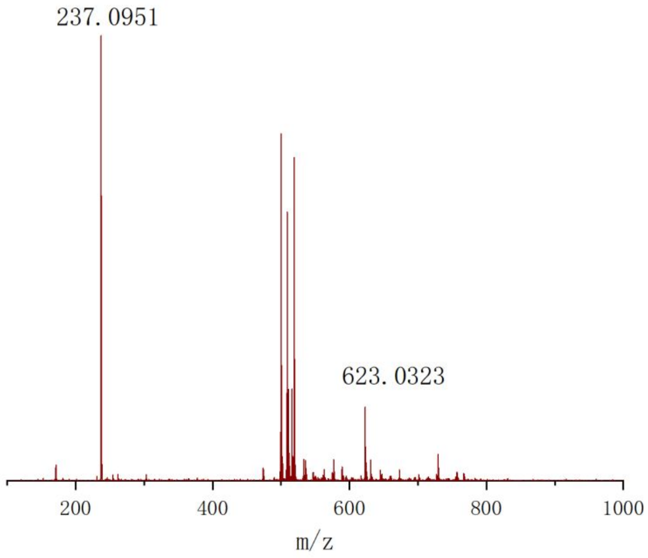

Figure S34. The HRMS of $[^{57}Fe^{II}(\mathbf{L^3})(CH_3CN)](OTf)_2$ in acetonitrile. The peaks at *m/z* = 237.0951 and *m/z* = 623.0323 correspond to the formulations $[^{57}Fe^{II}(\mathbf{L^3})]^{2+}$ (calc. 237.0651) and $[^{57}Fe^{II}(\mathbf{L^3})(OTf)]^{+}$ (calc. 623.0828), respectively.

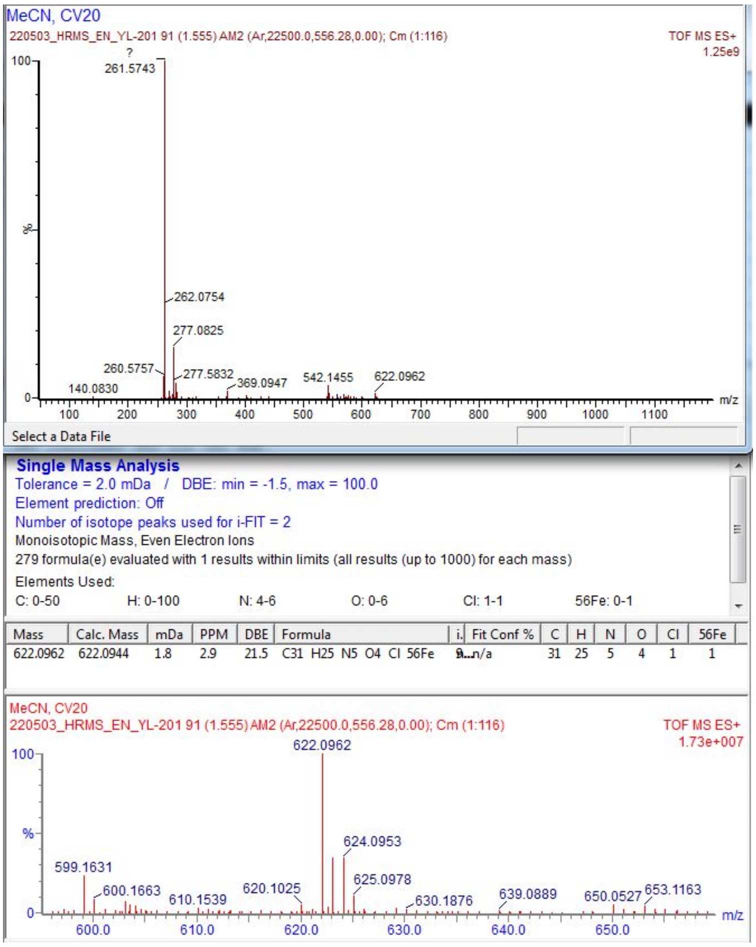

Figure S35. The HRMS of **4a**·$(ClO_4)_2$ in acetonitrile. The peaks at *m/z* = 261.5743 and *m/z* = 622.0962 correspond to the formulations $[Fe^{II}(\mathbf{L^4})]^{2+}$ (calc. 261.5730) and $[Fe^{II}(\mathbf{L^4})(ClO_4)]^{+}$ (calc. 622.0944), respectively.

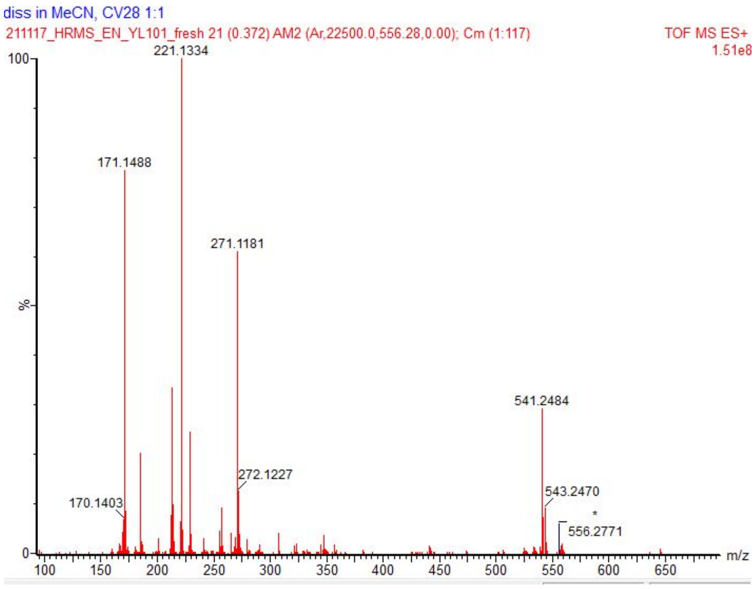

Figure S36. The HRMS data of **1b**·$(ClO_4)_2$ in acetonitrile. The peaks at *m/z* = 221.1334 and *m/z* = 541.2484 correspond to the formulations $[Fe^{IV}=O(\mathbf{L^1})]^{2+}$ (calc. 221.0602) and $[Fe^{IV}=O(\mathbf{L^1})(ClO_4)]^{+}$ (calc. 541.0690), respectively.

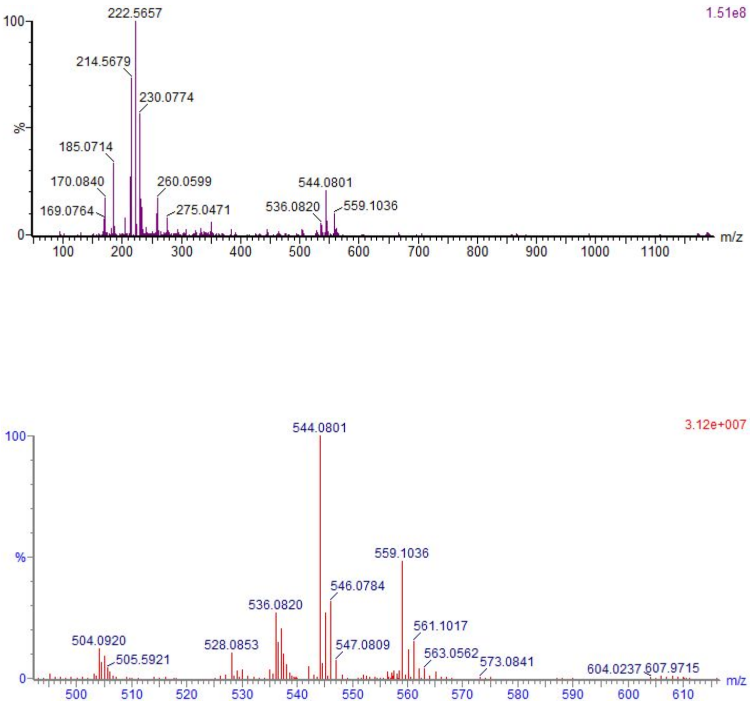

Figure S37. The HRMS of **2b**·$(ClO_4)_2$ in acetonitrile. The peaks at *m/z* = 222.5657 and *m/z* = 544.0801 correspond to the formulations $[Fe^{IV}=O(\mathbf{L^2})]^{2+}$ (calc. 222.5657) and $[Fe^{IV}=O(\mathbf{L^2})(ClO_4)]^{+}$ (calc. 544.0799.), respectively. The peaks at m/z 230.0744 and 559.1036 corresponds to $[Fe^{III}(OCH_3)(\mathbf{L^2})]^{2+}$ and $[Fe^{III}(OCH_3)(\mathbf{L^2})(ClO_4)]^{1+}$, respectively. The peaks at *m/z* = 214.5679 corresponds to $[Fe^{II}(ClO_4)]^{2+}$.

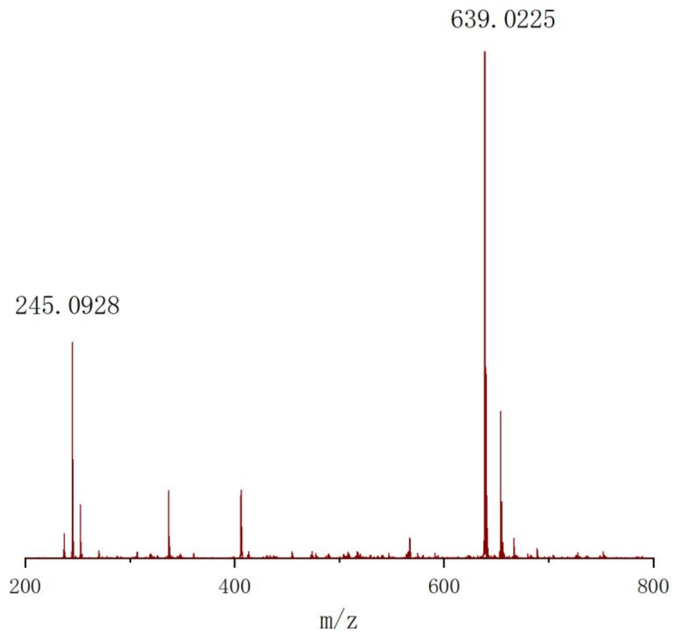

Figure S38. The HRMS of **3b**·(OTf)$_2$ in acetonitrile. The peaks at *m/z* = 245.0928 and *m/z* = 639.0225 correspond to the formulations [$^{57}Fe^{IV}$=O(**L**^{3})]$^{2+}$ (calc. 245.0628) and [$^{57}Fe^{IV}$=O(**L**^{3})(OTf)]$^+$ (calc. 639.0776), respectively.

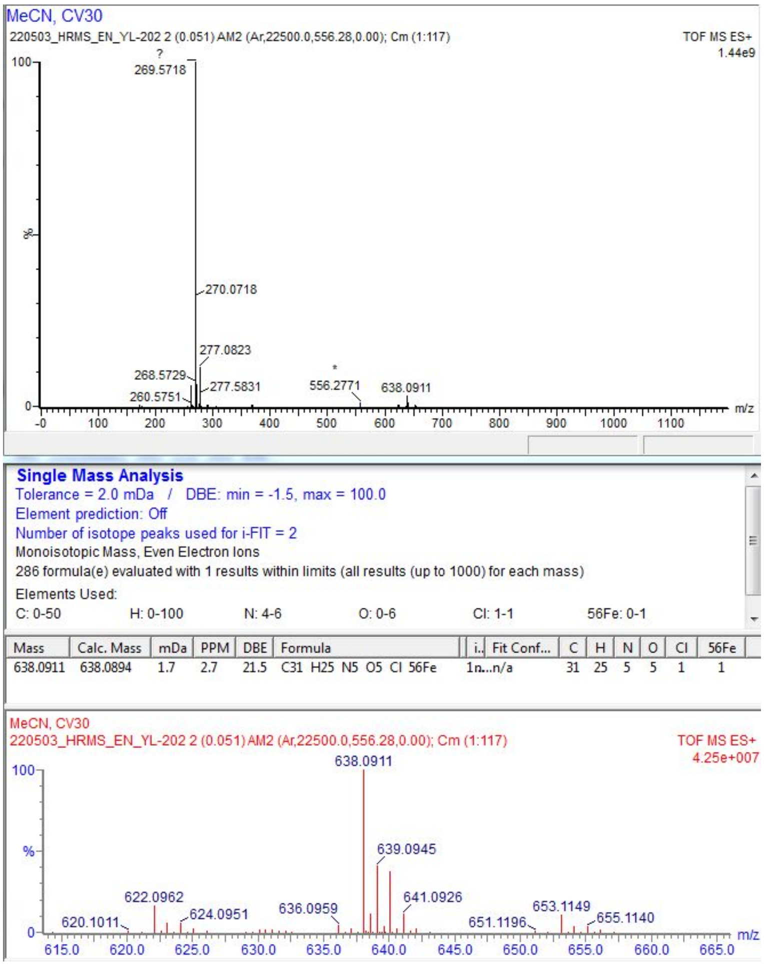

Figure S39. The HRMS of **4b**·$(ClO_4)_2$ in acetonitrile. The peaks at m/z = 269.5718 and m/z = 638.0911 correspond to the formulations $[Fe^{IV}{=}O(\mathbf{L}^4)]^{2+}$ (calc. 269.5704) and $[Fe^{IV}{=}O(\mathbf{L}^4)(ClO_4)]^{+}$ (calc. 638.0894.), respectively.

Table S7. Selected bond distances (Å) and bond angles (º) of complex **4b**

Fe(1)-O(1)	1.6584(14)
Fe(1)-N(1)	1.9546(17)
Fe(1)-N(4)	1.9608(17)
Fe(1)-N(2)	1.9685(17)
Fe(1)-N(3)	1.9709(17)
Fe(1)-N(5)	2.0486(16)
O(1)-Fe(1)-N(1)	97.80(7)
O(1)-Fe(1)-N(4)	97.12(7)
N(1)-Fe(1)-N(4)	85.92(7)
O(1)-Fe(1)-N(2)	98.09(7)
N(1)-Fe(1)-N(2)	91.50(7)
N(4)-Fe(1)-N(2)	164.78(7)
O(1)-Fe(1)-N(3)	96.99(7)
N(1)-Fe(1)-N(3)	165.19(7)
N(4)-Fe(1)-N(3)	91.55(7)
N(2)-Fe(1)-N(3)	87.12(7)
O(1)-Fe(1)-N(5)	177.61(7)
N(1)-Fe(1)-N(5)	84.58(7)
N(4)-Fe(1)-N(5)	83.08(7)
N(2)-Fe(1)-N(5)	81.75(7)
N(3)-Fe(1)-N(5)	80.63(7)

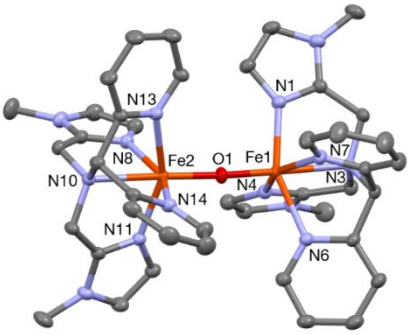

Table S8. Selected bond distances (Å) and bond angles (º) of complex **5**·$(OTf)_2$

Fe(1)-O(1)	1.7665(11)
Fe(1)-N(1)	2.0727(15)
Fe(1)-N(4)	2.0786(14)
Fe(1)-N(7)	2.1624(14)
Fe(1)-N(6)	2.1646(14)
Fe(1)-N(3)	2.2852(14)
Fe(2)-O(1)	1.7673(11)
Fe(2)-N(8)	2.0687(14)
Fe(2)-N(11)	2.0746(14)
Fe(2)-N(14)	2.1577(14)
Fe(2)-N(13)	2.1792(14)
Fe(2)-N(10)	2.2841(14)
O(1)-Fe(1)-N(1)	102.66(6)
O(1)-Fe(1)-N(4)	105.75(6)
N(1)-Fe(1)-N(4)	90.88(6)
O(1)-Fe(1)-N(7)	100.65(6)
N(1)-Fe(1)-N(7)	90.53(6)
N(4)-Fe(1)-N(7)	152.56(6)
O(1)-Fe(1)-N(6)	103.97(6)
N(1)-Fe(1)-N(6)	152.90(6)
N(4)-Fe(1)-N(6)	86.71(6)
N(7)-Fe(1)-N(6)	79.74(5)
O(1)-Fe(1)-N(3)	175.56(6)
N(1)-Fe(1)-N(3)	77.50(5)
N(4)-Fe(1)-N(3)	78.67(5)
N(7)-Fe(1)-N(3)	74.91(5)
N(6)-Fe(1)-N(3)	75.56(5)
O(1)-Fe(2)-N(8)	104.84(6)
O(1)-Fe(2)-N(11)	102.46(6)
N(8)-Fe(2)-N(11)	89.55(6)
O(1)-Fe(2)-N(14)	101.79(6)
N(8)-Fe(2)-N(14)	152.57(6)
N(11)-Fe(2)-N(14)	91.10(6)
O(1)-Fe(2)-N(13)	104.39(6)
N(8)-Fe(2)-N(13)	87.54(5)
N(11)-Fe(2)-N(13)	152.83(5)
N(14)-Fe(2)-N(13)	79.53(5)
O(1)-Fe(2)-N(10)	176.58(6)
N(8)-Fe(2)-N(10)	78.58(5)
N(11)-Fe(2)-N(10)	77.53(5)
N(14)-Fe(2)-N(10)	74.80(5)
N(13)-Fe(2)-N(10)	75.42(5)

Fe(1)-O(1)-Fe(2) 170.53(8)

Evans measurements of spin states of Fe^{IV}=O complexes

Evans measurements were performed using tert-butanol as reference in the form of a 10% v/v solution in CD_3CN. Two separate melting point capillary tubes were filled with the tert-butanol solution and the solution of Fe^{IV}=O complex in CD_3CN and sealed. The capillary tubes were inserted into separate NMR tubes and filled with CD_3CN. The data in the NMR spectra were analyzed using the equation below:

$$X_m = \frac{6}{1000}\frac{1}{c}\frac{\Delta f}{f} \quad (1)$$

$$\mu_{eff} = 798\sqrt{X_m T} = \sqrt{n(n+2)} \quad (2)$$

where X_m is the molar magnetic susceptibility of the transition metal complex, c is the concentration of the complex (mol/L), Δf is the shift in the frequency of the reference compound (Hz), and f is the frequency of the spectrometer (Hz), T is the temperature in Kelvin, and n is the number of unpaired electrons in the metal ion. Figs S40, S41 and S42 confirmed two unpaired electrons (μ_{eff} (spin only) = 2.83 μ_B) for complexes **1b**, **3b** and **4b**.

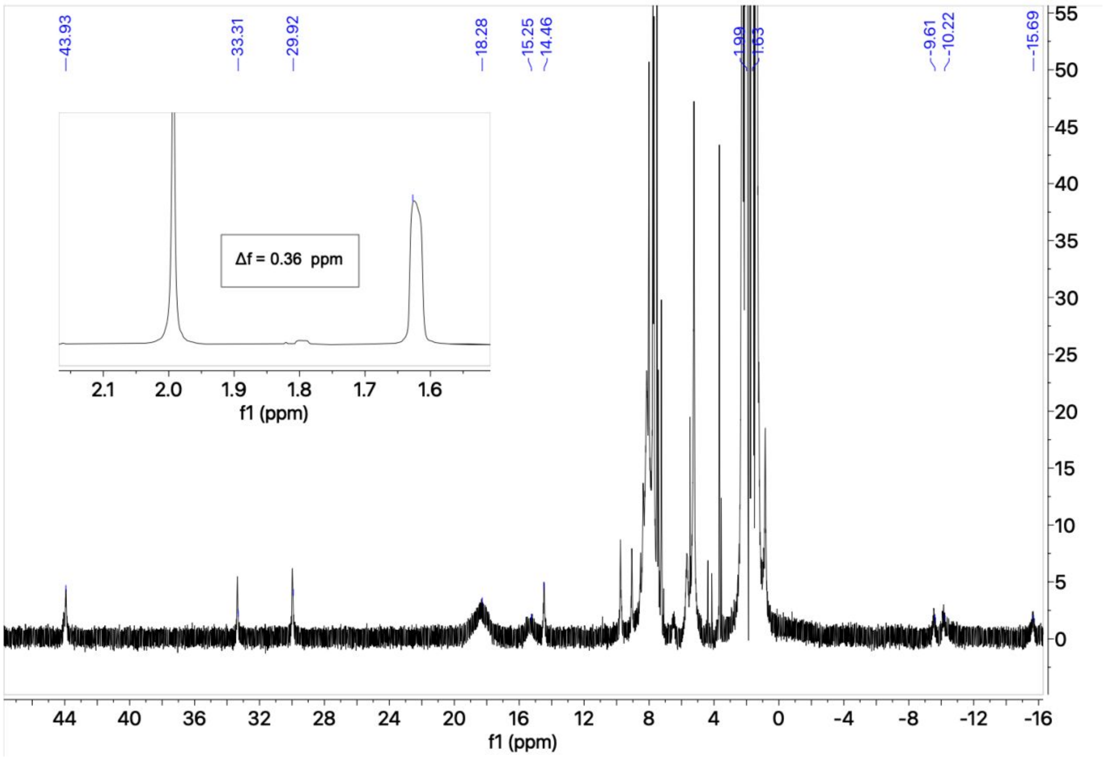

Figure S40. The Evans NMR spectrum of complex **1b**·$(ClO_4)_2$ in CD_3CN measured at 298 K; μ_{eff} = 2.78 μ_B

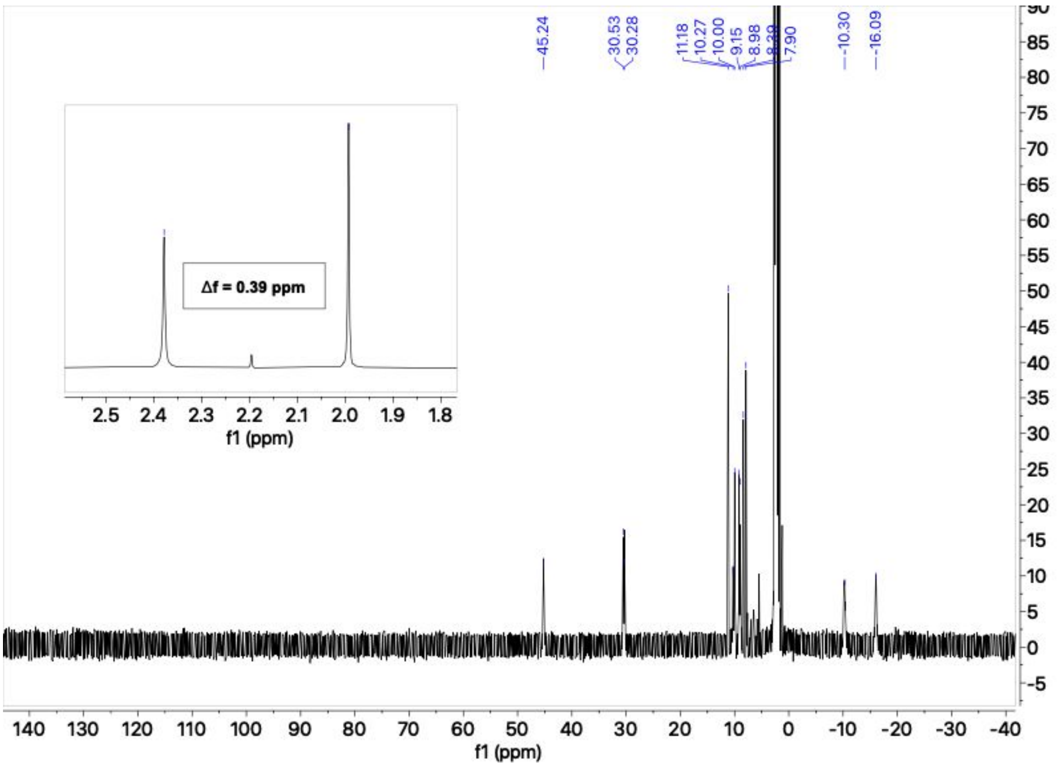

Figure S41. The Evans NMR spectrum of complex **3b**·$(ClO_4)_2$ in CD_3CN measured at 298 K; μ_{eff} = 2.63 μ_B

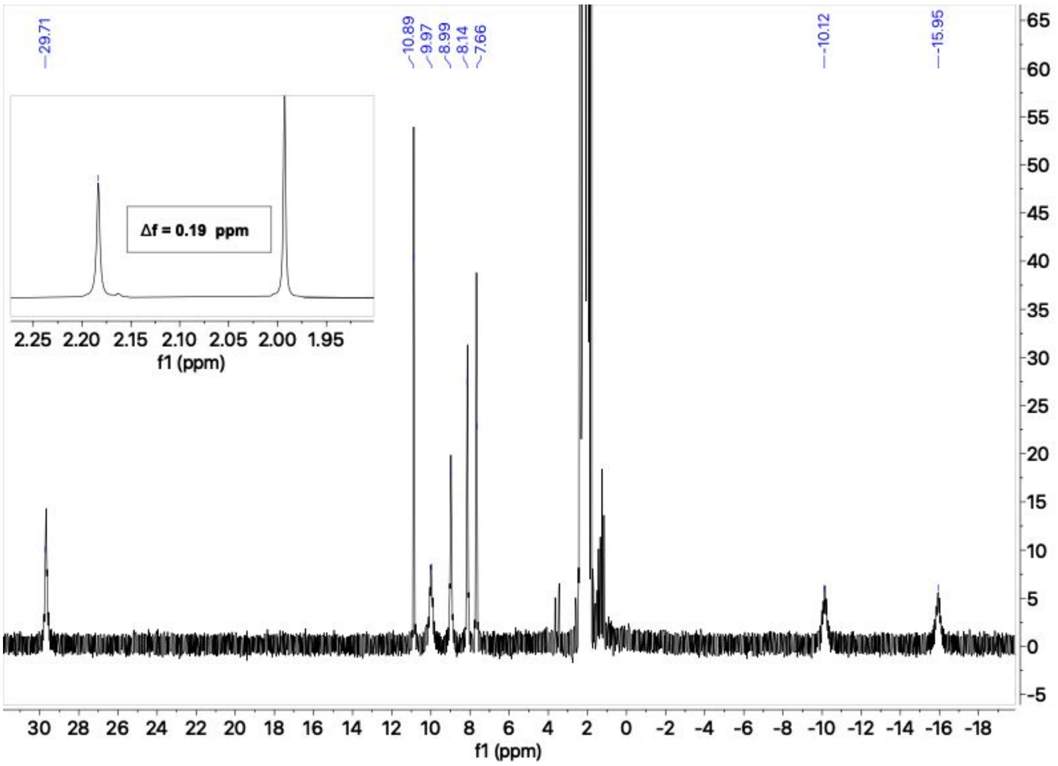

Figure S42. The Evans NMR spectrum of complex **4b**·$(ClO_4)_2$ in CD_3CN measured at 298 K; μ_{eff} = 2.71μ_B

IR spectra of Fe^{II} and Fe^{IV}=O complexes

Samples for infrared spectroscopy were prepared as KBr pellets. CAUTION: Perchlorate salts are potentially explosive and should be handled with care. Complexes **3b**/**4b** display ν(Fe=O) features at 839 cm^{-1} (Figs S47 and S50).

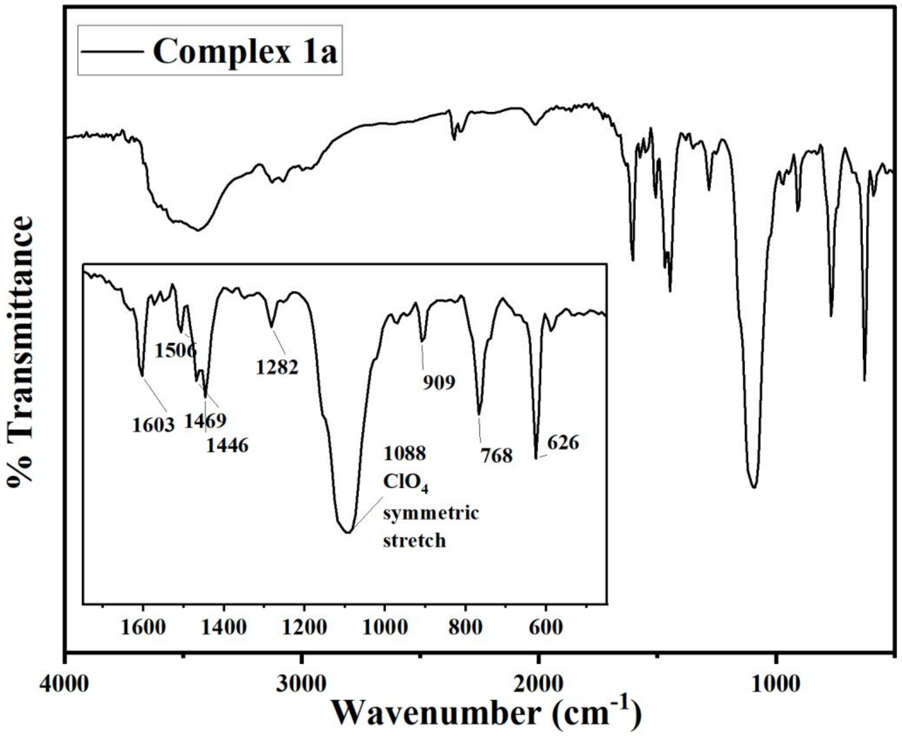

Figure S43. The FTIR spectrum of **1a**·$(ClO_4)_2$ in a KBr pellet.

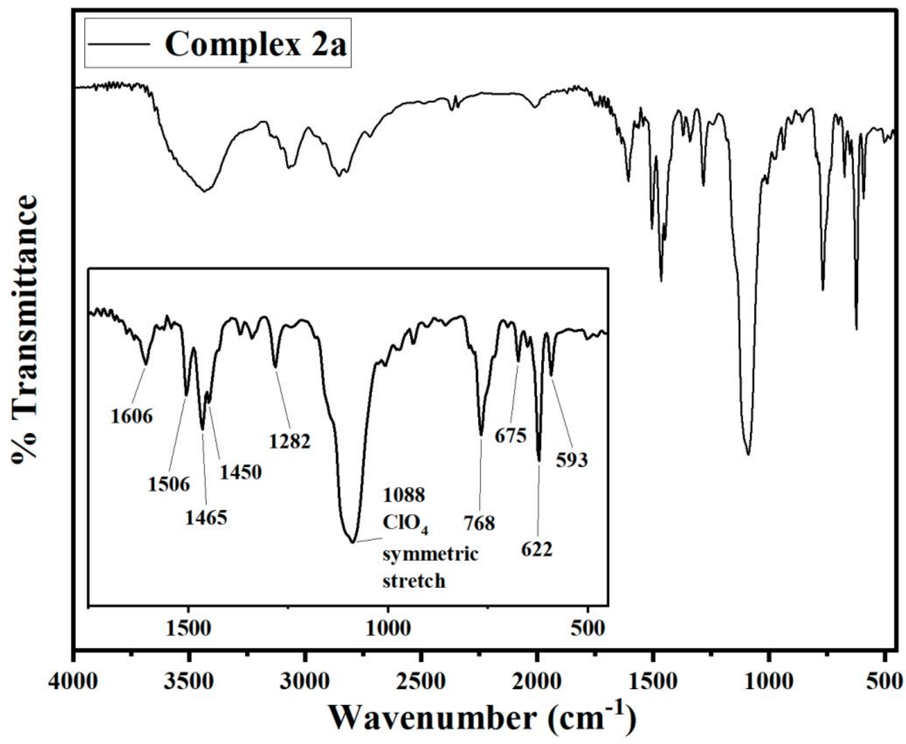

Figure S44. The FTIR spectrum of **2a**·$(ClO_4)_2$ in a KBr pellet.

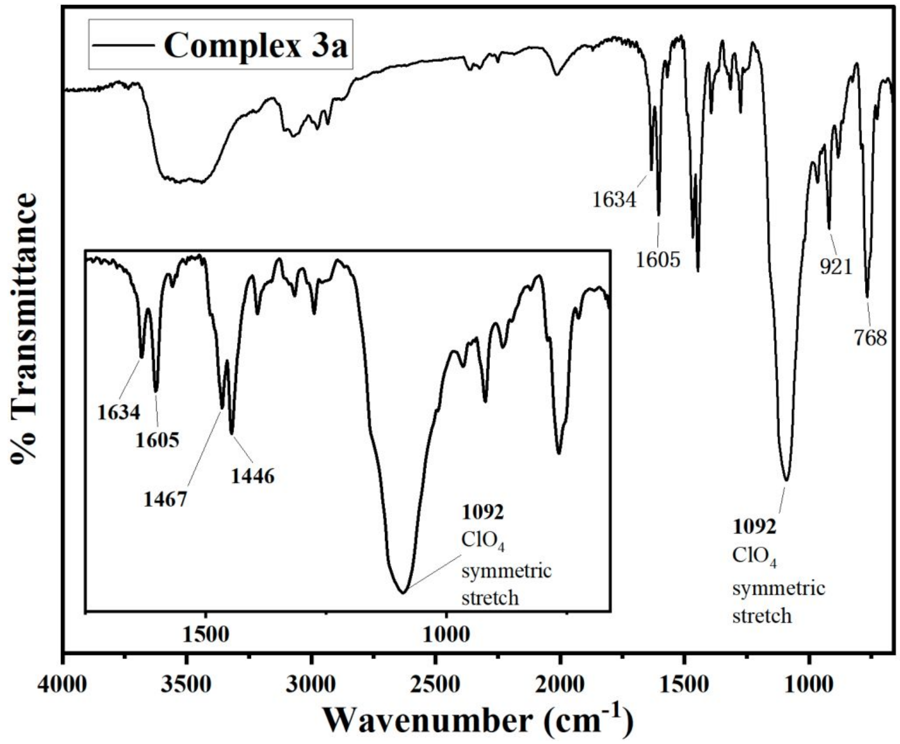

Figure S45. The FTIR spectrum of **3a**·$(ClO_4)_2$ in a KBr pellet.

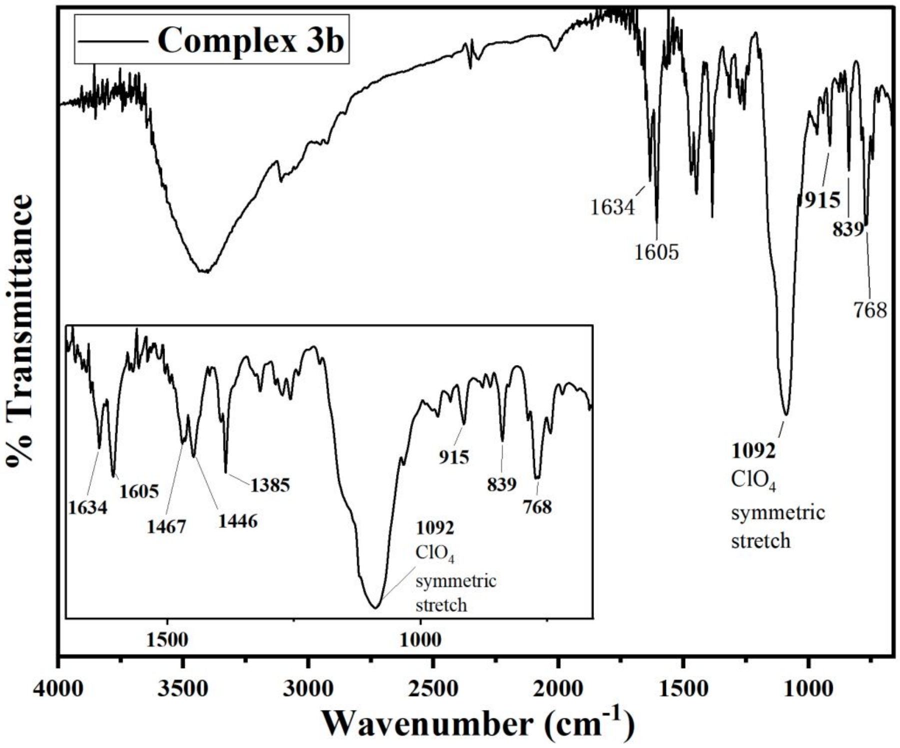

Figure S46. The FTIR spectrum of **3b**·$(ClO_4)_2$ in a KBr pellet.

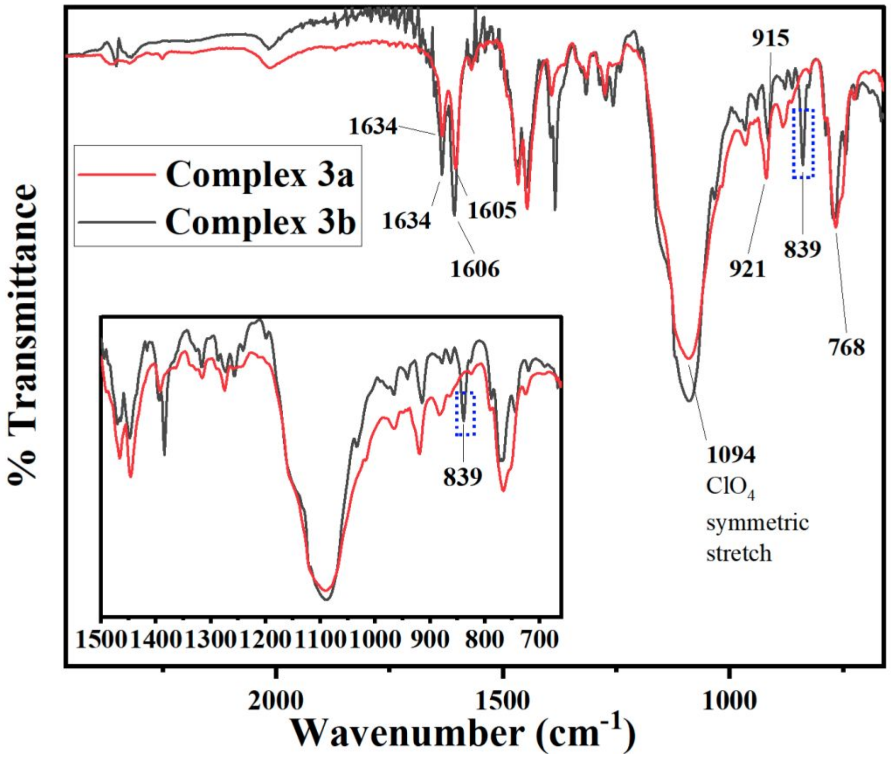

Figure S47. The FTIR spectra of **3a**·$(ClO_4)_2$ and **3b**·$(ClO_4)_2$ in KBr pellets.

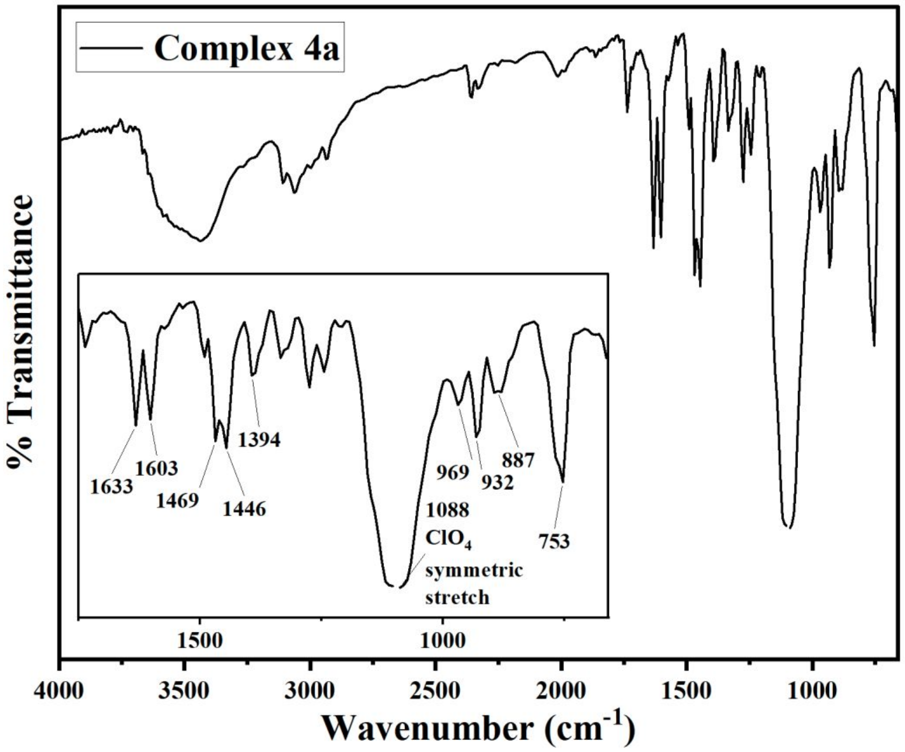

Figure S48. The FTIR spectrum of **4a**·$(ClO_4)_2$ in a KBr pellet.

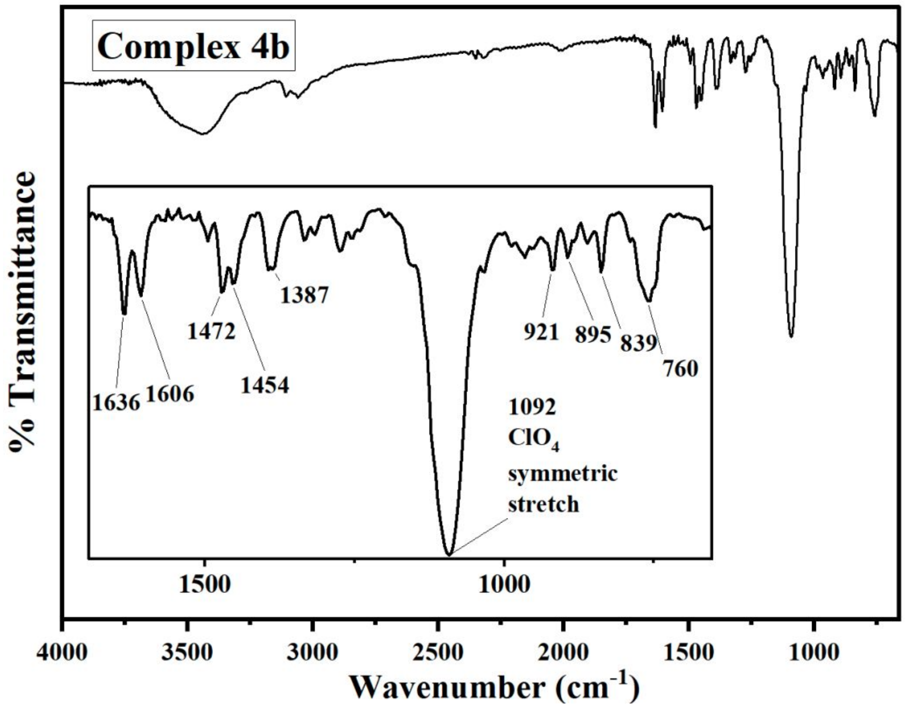

Figure S49. The FTIR spectrum of **4b**·$(ClO_4)_2$ in a KBr pellet.

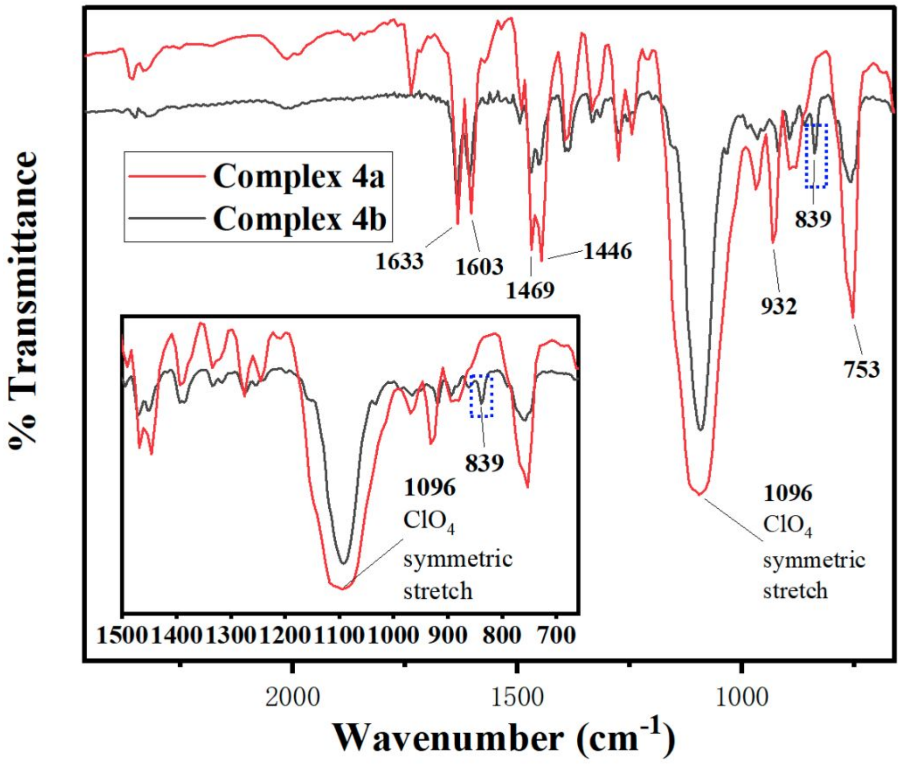

Figure S50. The FTIR spectra of **4a**·$(ClO_4)_2$ and **4b**·$(ClO_4)_2$ in KBr pellets.

DFT modeling of the structures

DFT calculations were performed to obtain the ground state geometry of the iron complexes within the PBE0/def2-TZVP(-f) level of theory. The optimized ground state geometry (Figure S 51) was compared to those obtained in the X-ray analysis, showing a good agreement (Table S9), where the maximum average error found for bond lengths and angles were 3.8% and 5.4%, respectively.

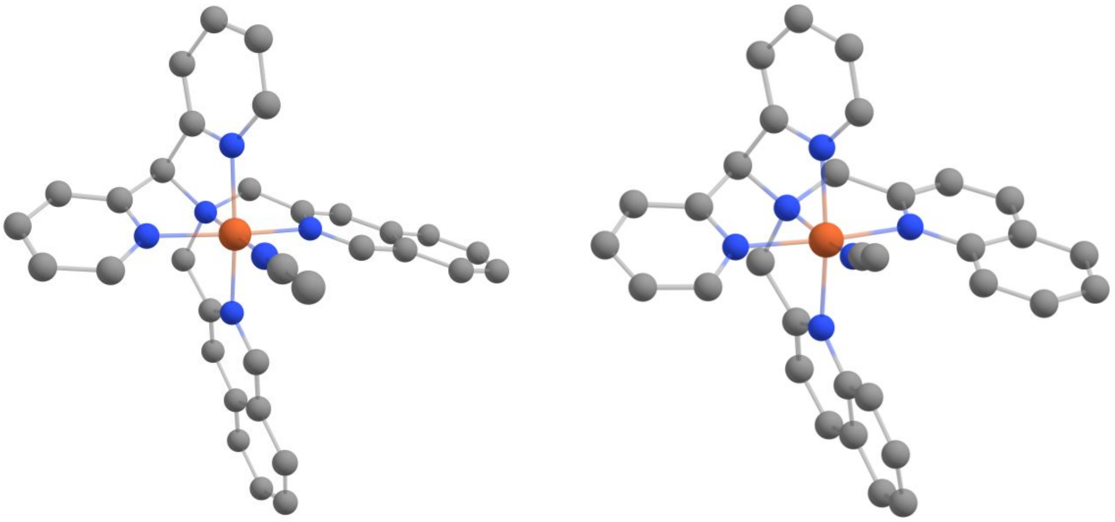

4a $[Fe(N_2Py_2Q)(CH_3CN)]^{2+}$

Figure S51. Optimized ground state geometries within PBE0/Def2-TZVP(-f) level of theory. Hydrogens are omitted for clarity.

Table S9. Selected bond lengths and angles for the calculated **4a** within PBE0/Def2-TZVP(-f) level of theory and some crystallographic data values for comparison. The numbering of atoms is the same as used for the crystallographic data.

	Theoretical	Experimental
N1-Fe	1.9807	1.968(5)
N2-Fe	1.9825	1.969(4)
N3-Fe	1.9783	1.977(5)
N4-Fe	1.9893	1.986(5)
N5-Fe	1.9879	1.984(4)
N6-Fe	1.9215	1.943(6)
N1-Fe-N2	86.16	91.5(2)
N1-Fe-N3	85.18	84.7(2)
N1-Fe-N5	92.15	92.8(2)
N1-Fe-N6	97.76	97.7(2)
N2-Fe-N3	85.04	86.8(2)
N2-Fe-N4	92.07	86.5(2)
N2-Fe-N6	97.71	95.4(2)
N3-Fe-N4	82.26	83.0(2)
N3-Fe-N5	82.48	82.5(2)
N4-Fe-N5	86.89	86.9(2)
N4-Fe-N6	94.80	94.6(2)
N5-Fe-N6	94.79	95.0(2).

Table S10. Selected bond lengths and angles for the calculated $[Fe(N_2Py_2Qn_2)(CH_3CN)]^{2+}$ within PBE0/Def2-TZVP(-f) level of theory and some crystallographic data values for comparison. The numbering of atoms is the same as used for the crystallographic data.

	Theoretical	Experimental
N1-Fe	1.9852	2.010 (5)
N2-Fe	1.9800	2.020 (5)
N3-Fe	1.9937	2.091 (5)
N4-Fe	2.0637	2.018 (5)
N5-Fe	2.0553	2.102 (5)
N6-Fe	1.9446	1.952 (6)
N1-Fe-N2	80.89	80.40 (19)
N1-Fe-N3	90.17	90.73 (18)
N1-Fe-N5	165.44	163.1 (2)
N1-Fe-N6	88.25	90.1 (2)
N2-Fe-N3	83.96	82.89 (18)
N2-Fe-N4	82.12	81.10 (19)
N2-Fe-N6	166.83	166.7 (2)
N3-Fe-N4	166.02	163.98 (19)
N3-Fe-N5	86.57	86.29 (18)
N4-Fe-N5	90.73	91.78 (18)
N4-Fe-N6	90.11	89.1 (2)
N5-Fe-N6	106.26	106.7 (2)
